# Phytoconstituent Profiles Associated with Relevant Antioxidant Potential and Variable Nutritive Effects of the Olive, Sweet Almond, and Black Mulberry Gemmotherapy Extracts

**DOI:** 10.3390/antiox12091717

**Published:** 2023-09-04

**Authors:** Amina Aleya, Emőke Mihok, Bence Pecsenye, Maria Jolji, Attila Kertész, Péter Bársony, Szabolcs Vígh, Zoltán Cziaky, Anna-Beáta Máthé, Ramona Flavia Burtescu, Neli-Kinga Oláh, Andreea-Adriana Neamțu, Violeta Turcuș, Endre Máthé

**Affiliations:** 1 Doctoral School of Animal Science, Faculty of Agricultural and Food Sciences and Environmental Management, University of Debrecen, Böszörményi Str. 128, 4032 Debrecen, Hungary; aleya.amina@agr.unideb.hu (A.A.); mihokemoke@uni.sapientia.ro (E.M.); kertesz@agr.unideb.hu (A.K.); 2 Doctoral School of Nutrition and Food Science, Faculty of Agricultural and Food Sciences and Environmental Management, University of Debrecen, Böszörményi Str. 128, 4032 Debrecen, Hungarymaria.jolji@agr.unideb.hu (M.J.); 3 Institute of Nutrition Science, Faculty of Agricultural and Food Sciences and Environmental Management, University of Debrecen, Böszörményi Str. 128, 4032 Debrecen, Hungary; endre.mathe@agr.unideb.hu; 4 Institute of Animal Science, Biotechnology and Nature Conservation, Faculty of Agricultural and Food Sciences and Environmental Management, University of Debrecen, Böszörményi Str. 128, 4032 Debrecen, Hungary; barsonp@agr.unideb.hu; 5 Agricultural and Molecular Research Institute, University of Nyíregyháza, Sóstói Str. 31, 4400 Nyíregyháza, Hungary; vigh.szabolcs@nye.hu (S.V.); cziaky.zoltan@nye.hu (Z.C.); 6 Doctoral School of Neuroscience, Faculty of Medicine, University of Debrecen, Nagyerdei Str. 94, 4032 Debrecen, Hungary; mathe.anna@med.unideb.hu; 7 PlantExtrakt Ltd., 407059 Cluj, Romania; ramona.burtescu@plantextrakt.ro (R.F.B.);; 8 Department of Pharmaceutical Chemistry, Faculty of Pharmacy, Vasile Goldiș Western University from Arad, L.Rebreanu Str. 86, 310414 Arad, Romania; 9 Department of Life Sciences, Faculty of Medicine, Vasile Goldiș Western University from Arad, L.Rebreanu Str. 86, 310414 Arad, Romania; 10 CE-MONT Mountain Economy Center, Costin C. Kirițescu National Institute of Economic Research, Romanian Academy, Petreni Str. 49, 725700 Suceava, Romania

**Keywords:** *Olea europaea*, *Prunus amygdalus*, *Morus nigra*, *Drosophila melanogaster*, *Cyprinus carpio*, gemmotherapy extract, antihyperglycemic effect, essential amino acid, tryptophane

## Abstract

The extracts of whole plants or specific organs from different plant species are gaining increasing attention for their phytotherapy applications. Accordingly, we prepared standardized gemmotherapy extracts (GTEs) from young shoots/buds of olive (*Olea europaea*), sweet almond (*Prunus amygdalus*), and black mulberry (*Morus nigra*), and analyzed the corresponding phytonutrient profiles. We identified 42, 103, and 109 phytonutrients in the olive, almond, and black mulberry GTEs, respectively, containing amino acids, vitamins, polyphenols, flavonoids, coumarins, alkaloids, iridoids, carboxylic acids, lignans, terpenoids, and others. In order to assess the physiological effects generated by the GTEs, we developed a translational nutrition model based on *Drosophila melanogaster* and *Cyprinus carpio*. The results indicate that GTEs could influence, to a variable extent, viability and ATP synthesis, even though both are dependent on the specific carbohydrate load of the applied diet and the amino acid and polyphenol pools provided by the GTEs. It seems, therefore, likely that the complex chemical composition of the GTEs offers nutritional properties that cannot be separated from the health-promoting mechanisms that ultimately increase viability and survival. Such an approach sets the paves the way for the nutritional genomic descriptions regarding GTE-associated health-promoting effects.

## 1. Introduction

Fruits and vegetables are known for their exceptional nutritional properties. The favorable macro- and micro-nutrient composition makes them especially suitable for human and animal consumption. Moreover, their rich phytonutrient or bioactive compound content could confer beneficial effects to consumers by aiding in homeostasis and increasing the efficiency of the adaptative stress responses. When assessed individually or in mixtures, the biological effects generated by phytonutrients are concentration-dependent; therefore, the toxicity/viability evaluation should be based on an assessment of a broader concentration range, allowing definition of the eventual hormetic response too [1]. Furthermore, the biological effects attributed to phytonutrient mixtures do not equal those of single phytonutrients studied individually. Gemmotherapy extracts (GTEs) are produced from plant organs such as buds and/or young shoots, and are rich in phytonutrients that need careful evaluation to allow for synergic, additive, and/or antagonistic effects to appear at the same level as the multi-compound matrices. 

Our research is intended to shed light on the phytoconstituent and nutritional properties of gemmotherapy extracts obtained from young shoots of olive (*Olea europaea*)—denoted further as O, fresh buds of sweet almond (*Prunus amygdalus* var. *dulcis*)—denoted further as SA, and black mulberry (*Morus nigra*)—denoted further as BM. These represent plant species whose other parts (mainly fruits and leaves) have already been suggested to have multiple health-promoting effects in the literature.

The olive belongs to the *Oleaceae* family, and is mostly found in Mediterranean countries, Sfax (south of Tunisia), and Jordan, as it is well adapted to arid conditions [2]. Starting from ancient Egypt, Greek and Rome, the cultivation of olive trees supported the production of olive oil; additionally, from the 16th century, soap production raised the cultivation needs, continuing to grow during the 19th and 20th centuries. The beneficial health effects of olive oil and leaf extract have been intensively studied, meaning that the anti-inflammatory and anti-microbial properties have been proven through substantial scientific evidence [3]. 

The almond is a fruit tree species that belongs to the *Rosaceae* family and has two varieties: wild (or bitter) almond and sweet almond. The wild or bitter almond presents a high content of amygdalin that breaks down during chewing to release benzaldehyde and hydrogen cyanide, with the latter being naturally toxic. Oppositely to hydrogen cyanide, the benzaldehyde is nontoxic and is responsible for the much appreciated “pure almond” and/or cherry flavor [4]. The sweet almond originates from a horticultural selection devoid of amygdalin, bearing edible seeds. Currently, California is the largest production region for sweet almond, accounting for nearly 80% of the world’s supply, but its popularity is also increasing in the Mediterranean region. Recently, almond consumption (in the form of seeds and flowers) started to rise due to its exceptional nutrient composition rich in proteins, unsaturated fatty acids, dietary fibers, and vitamin E [5]. 

The black mulberry is a deciduous tree that belongs to the *Moraceae* family. It is speculated to originate from Western Asia and to have been cultivated since the Iron Age [6]; currently, it continues to gain popularity in the Mediterranean area, America, and some regions in Africa [7]. Increasing attention dedicated to phytonutrients and their impact on human health has given the black mulberry a spotlight in nutritional research [8], since the fruits are rich in phenolic compounds, flavanols, anthocyanins (black and red fruits), and alkaloids, with a wide range of pharmacological properties [9,10,11,12]. 

In the current study, we report the phytonutrient profile of three GTEs—specifically, for olive, sweet almond, and black mulberry plant species. The qualitatively and quantitatively described bioactive compounds prove the complex composition of each GTE, and, alongside their antioxidant potential, they might generate multiple health-promoting physiological effects. Moreover, the analyzed macronutrient content and the ATP-generating potential have set light on the concentration-dependent nutritive features of the GTEs. For this purpose, a translational approach has been defined, encompassing fruit fly (*Drosophila melanogaster*)- and carp (*Cyprinus carpio*)-based models. Careful evaluation of the life cycle-specific developmental stages using fruit fly and carp denoted different nutritive/nutritional properties and no toxic effects associated with the studied GTEs. These observations suggest that the GTEs’ specific health-promoting effects could be related not only to their phytoconstituent profile, but also to their ATP-generating properties. 

## 2. Materials and Methods

### 2.1. Preparation of GTEs

Young shoots of olive trees and buds of sweet almond were harvested in June and April 2020, respectively, in Calabria, the southern part of Italy, from an organic farm. The buds of black mulberry were collected in April 2020 from an organic culture farm from Așchileu Mare, Cluj, Romania, of SC PlantExtrakt SRL. The vegetal materials were further monitored to meet the quality standards at the laboratories of SC PlantExtrakt SRL QC, and voucher specimens were retained. 

The GTEs were always prepared from freshly harvested vegetal materials that were preserved in a 1:1 mixture of 96% (*v*/*v*) ethanol and glycerol, with a plant-to-solvent ratio of 1:0.5. Samples were retained before preservation from each vegetal species and kept at 4 °C until arriving to Quality Control Laboratory. The moisture analysis was performed using the abovementioned samples. Based on the determined moisture content, the solvent quantity was computed to achieve a ratio of dry plant-to-solvent of 1:20. The solvent volume used initially was deduced at this stage. The solvent was a 1:1 mixture of 96% (*v*/*v*) ethanol and glycerol. The vegetal material was crushed into the preservation solvent, the calculated solvent was added, and the obtained plant–solvent mixture was mixed periodically for 20 days, 2 × 20 min/day. Next, the solid and the liquid parts of the mixture were separated, and the extracted solid plant material was further pressed to increase the yield of extraction. The two extracted solutions were mixed, forming the concentrated GTEs that were used in further studies [13].

### 2.2. Analysis of Phytonutrient Profiles of GTEs by UHPLC–ESI-MS

The experiments were carried out with a Dionex Ultimate 3000RS ultrahigh pressure liquid chromatography (UHPLC) system equipped with a Thermo Scientific Accucore C18 column, with L./I.D. of 100/2.1 mm (particle size of 2.6 μm), that was coupled to a Thermo Q Exactive Orbitrap mass spectrometer (MS) combined with an electrospray ionization source (ESI), having an accuracy of 5 ppm. The data acquisition and further analysis were carried out as described by Neamtu et al. [14].

### 2.3. Spectrophotometric Determination of Total Polyphenol Content (TPC) of GTEs

The TPC was determined using 0.1 mL of a GTE to which 0.5 mL phosphotungstenic reagent was added, and the solutions were diluted to 25 mL with 15% sodium carbonate solution. The samples were incubated for 2 min, then the absorbance was measured at 715 nm; the blank consisted of 0.1 mL extract diluted to 25 mL with 15% sodium carbonate solution and was subtracted from the measurement. The absorbance was measured using a Cintra 101 UV–Vis spectrophotometer (GBC, Keysborough, Australia). The same protocol was applied for the gallic acid solutions in a concentration range of 22–88 µg/mL. The calibration curve (for detailed analyses, see Supplementary Materials Figure S1) correlation factor was 0.9717. The TPC of the GTE was expressed in gallic acid equivalents (GAE)/g dry weight plant material. All reagents were of analytical grade, purchased from Merck (Darmstadt, Germany). All analyses were performed in triplicate and the results were statistically evaluated using Excel (Microsoft Office).

### 2.4. Spectrophotometric Determination of Total Flavonoid Content (TFC) of GTEs

The TFC was determined by a colorimetric method adapted from the Romanian Pharmacopoea [15].

In a 25 mL volumetric flask, 1 mL of the GTE was mixed with 5 mL of a sodium acetate solution (100 g/L) and 3 mL of an aluminum chloride solution (25 g/L), then brought to 25 mL final volume with methanol. The mixture was shaken and left to stand for 15 min at room temperature before the measurements were carried out. The absorbance was recorded at 430 nm, using a Cintra 101 UV–Vis spectrophotometer (GBC, Keysborough, Australia). The blank was 1 mL GTE, 8 mL water, and methanol to 25 mL total volume. Rutoside was used as standard, and solutions in the concentration range 4–20 µg/mL were used in order to build the calibration curve (for detailed analyses, see Supplementary Materials Figure S2). The calibration curve had a correlation factor of 0.9970. The TFC of GTEs was expressed in rutoside equivalent (RE)/mL. All reagents were of analytical grade, purchased from Merck (Darmstadt, Germany). All analyses were performed in triplicate and the results were statistically evaluated using Excel (Microsoft Office).

### 2.5. The Determination of Antioxidant Potential of GTEs by Spectrophotometric Methods

The antioxidant potential was determined through DPPH, FRAP, and Xanthine oxidase inhibition assays, using a Cintra 101 UV–Vis spectrophotometer (GBC, Keysborough, Australia).

#### 2.5.1. DPPH Assay

In the DPPH assay, the samples were prepared using 4, 8, and 12 µL/mL O GTE, and 8, 16 and 24 µL/mL SM and BM GTE, which were mixed with 5 mL of DPPH reagent and incubated for 30 min at 40 °C. The absorbance of the samples was measured at 517 nm, and the blank containing methanol was subtracted. The control used was 5 mL methanol and 5 mL DPPH reagent [16]. The % inhibition of each sample was calculated using the following formula:

$$\%\;inhibition=\left(Absorbance\;of\;control-Absorbance\;of\;sample\right)\times 100/(Absorbance\;of\;control)$$

The calibration curves of % inhibition as a function of the concentration, and the IC_50_ was also calculated; all are presented in the Supplementary Materials (for detailed analyses, see Supplementary Materials Figures S3–S5).

#### 2.5.2. FRAP Assay

The FRAP assay samples consisted of 0.02 mL GTE, brought to 0.2 mL with methanol, followed by mixing with 0.6 mL of water and 6 mL of FRAP reagent. The samples were incubated for 5 min and then measured at 593 nm, against a blank prepared from 0.8 mL water and 6 mL of FRAP reagent. For the Trolox calibration, a concentration range of 12.5–50.0 μg/mL was used. The antioxidant potential of GTEs was expressed in μM Trolox equivalent/100 mL extract [16].

#### 2.5.3. Xanthine Oxidase Assay

The xanthine oxidase inhibition assay used samples prepared from 0.15 mL of GTE, to which 3.9 mL of phosphate buffer pH = 7.4 and 0.6 mL of xanthine oxidase 0.2 IU/mL were added. The obtained samples were incubated at 25 °C for 10 min, then 4.5 mL of 0.15 mM xanthine was added and further incubated for 30 min at 25 °C. A control was prepared using 1.5 mL of methanol substituting the GTE, while the blank contained methanol replacing the GTE and xanthine oxidase enzyme. The absorbance of the samples was measured at 293 nm and the blank was subtracted. The % inhibition was calculated according to the formula described for the DPPH assay [16].

### 2.6. Quantitative Analysis of GTE Specific Selected Polyphenols by UHPLC–ESI-MS

A Shimadzu Nexera I LC-MS-8045 (Kyoto, Japan) UHPLC system, equipped with a quaternary pump and autosampler connected, respectively, to an ESI probe and quadrupole rod mass spectrometer, was used in the quantitative determination of selected polyphenols. The separation was carried out on a Luna C18 reversed phase column (150 mm × 4.6 mm × 3 μm, 100 Å), from Phenomenex (Torrance, CA, USA). The column was maintained at 40 °C. The mobile phase (for detailed analyses, see Supplementary Materials Table S1) was a gradient made from methanol purchased from Merck (Darmstadt, Germany) and ultra-purified water prepared using Simplicity Ultra Pure Water Purification System from Merck Millipore (Billerica, MA, USA). As an organic modifier, formic acid purchased from Merck (Darmstadt, Germany) was used. The methanol and the formic acid were of LC/MS grade. The applied flow rate was 0.5 mL/min. The total time of the analysis was 35 min. The detection was performed on a quadrupole rod mass spectrometer operated with electrospray ionization (ESI), both in negative and positive multiple reaction-monitoring (MRM) ion mode (Table 2). The interface temperature was set at 300 °C. For vaporization and as a drying gas, nitrogen was used at 35 psi and 10 L/min, respectively. The capillary potential was set at +3000 V.

The polyphenol standards, together with the calibration curves equations, their correlation factors, and the limits of detection and quantification, are presented in Table 1. The evaluation of the standards’ specific concentrations was carried out using 1 μL of sample. The identifications were performed by comparing the MS spectra and their transitions between the separated polyphenols and standards. The identification and quantification were made based on the main transition from the MS spectra for each compound. For quantification, the calibration curves were also determined (see the equations in Table 1). Table 2 presents the retention times and specific MS spectral data for the standards analyzed. In the Supplementary Materials section, we show the chromatograms (for detailed analyses, see Supplementary Materials Figures S6–S17) and the MS spectra of the standards (for detailed analyses, see Supplementary Materials Figures S18–S29). 

### 2.7. Determination of the GTE-Specific Macronutrient Content

The protein content was assessed by the Kjeldahl method. Total carbohydrate content was determined by the phenol–sulfuric acid method [17], which can detect all classes of carbohydrates, including mono-, di-, oligo-, and polysaccharides. The Luff–Schoorl method was used for determining the reducing sugars [18].

### 2.8. The Drosophila Melanogaster Stocks, Culture Media, and Viability Experiments

In all *Drosophila melanogaster* experiments, the w^m4h^ (white mottled 4) strain was used, which had been obtained from the Bloomington Stock Center. The flies were raised employing three types of dietary conditions: (1) zero nutrient—denoted as 0N; (2) normal media—denoted as NM; and (3) high-sugar media—denoted as HS. The 0N media was prepared using 1 g of carbon powder mixed with 1 g of agar (VWR, No. 20767.298) in 100 mL of water, boiled for 10 s, then cooled to 45 °C, and aliquoted into vials containing 3 mL. The 0N resembles a minimal larval media [19] but does not contain any nutrients. For the other culture media (NM and HS), 70 g of yeast paste was mixed with 1.2 L of water until homogeneity, then 51.35 g (for NM) or 513.45 g (for HS) of sucrose and 30 g of wheat flour were added and brought to boil. Then, 10 g of agar powder was added after thorough mixing. The boiling continued for over 30 min and the culture media reached a final volume of 1 L. Next, the culture media was cooled in a water bath to 50 °C, and 1 g of NIPAGIN purchased from ThermoFisher was mixed in thoroughly; then, 4 mL of culture media were aliquoted into each culture vial. Whenever appropriate, next to the culture media, a given volume of GTE was added and mixed thoroughly. The GTEs were set at five different concentrations: 11%, 20%, 33.3%, 42.8%, and 50% (by mixing 0.5 mL, 1 mL, 2 mL, 3 mL, and 4 mL of extracts with 4 mL of culture media). All the fruit fly experiments were performed at 25 °C.

The collection of 0–2 h *Drosophila melanogaster* embryos:

About two hundred 5-day old female and male w^m4h^ *Drosophila melanogaster* individuals were introduced inside an embryo collection cage placed over a plate containing 0N media supplemented with yeast paste. After 48 h, with replacement every 2 h of the egg collecting plates, 0–2 h-old embryos could be obtained. The embryos were removed with fine forceps under the microscope from the plate, and introduced into a vial with the corresponding 0N, NM, or HS media supplemented with GTEs. A total of 50 embryos were placed into each vial, and 5 vials were set for each considered concentration. The experiments were carried out in triplicate and averaged.

Monitoring the viability during *Drosophila melanogaster* development:

The experiments were carried out at 25 °C and constant humidity. The number of third instar larvae and adults were monitored daily until no more hatched adults were found. The experiments were repeated three times, and included a wider GTE-specific concentration range to obtain a more profound picture of the viability effect. We should also emphasize that the GTE-specific viability tests were based on flies with the same genotype and age, and all the experiments were performed in parallel, so that the observed results would be fully comparable. 

### 2.9. The Carp (Cyprinus carpio)-Based Larval Viability Tests 

The experiments were set up in the Fish Laboratory Unit of the Institute of Animal Science, Biotechnology, and Nature Conservation. After successful artificial propagation of common carp, the fertilized eggs were placed in Zuger glasses with an independent mechanical and biological filter being connected to a recirculation system. In here, the larvae started to hatch after 48 h, so that the viable and non-feeding fries were transferred to a fry-rearing unit that was again connected to a recirculation system.

Then, the herd was kept for 48 h until their first breath, so that the larva could fill up their swim bladder. Next, about 200 larvae were placed in the units of a modular aquarium system, serving as the location of the experiment; the total water volume was about 800 L. This system consisted of 12 aquariums, each having a useful volume of 20 L, while the buffer tank had a volume of 320 L. We placed 200 non-feeding larvae per aquarium, based on a random block arrangement. In this aquarium system, the mechanical filtration was carried out with a 20 L volume ceramic medium with a useful surface area of 40 m^2^, and the biological filtration employed a 50 L volume and 20 µm particle size sponge system. The JEBAO AIR PUMP compressor supplied the oxygen, ensuring the desired saturation level. In addition, 500 W Aqua L GOLD 500 aquarist heating elements were used to maintain the appropriate temperature. The temperature (22.0 ± 0.5 °C) and the dissolved oxygen concentration (79.3 ± 0.6%) were also monitored daily using a HACH HQ30d measuring device, and they remained constant for the duration of the experiments. 

In the experiment, the fish larval groups were fed ad libitum. At the end of each day, the excess feed and excrement were vacuumed off the aquariums. The experiment consisted of four treatments, as shown in Table 3, while the mentioned experimental set up was repeated three times.

The control groups were fed with the brine shrimp (*Artemia salina*) type of live food for the entire duration of the experiment. In the remaining aquariums, the carp larvae groups were fed with the studied GTE-specific food. During the time of the experiments, the fish larvae were assessed for their length and ATP content. At the end of the experiment, the viable individuals were counted and the survival rate was determined.

Development of the GTE-based feed for fish larvae:

In order to develop a fishmeal suitable for carp larvae, Fibersol-2 water-soluble dietary fiber was mixed with the given GTE, with 0.2 mL of GTE added to 1 g of fiber; this was mixed until a homogeneous consistency was reached, and the mixture was dried for 2–3 days in an incubator at 35 °C. Finally, the GTE-Fibresol-2 dried mix was ground to obtain a uniform grain size, and stored at room temperature in closed recipients, protected from light until usage.

### 2.10. Determination of the ATP Content of Fish Larvae

For every sample, 10 fish larvae were selected from the corresponding aquariums and placed in a 1.5 mL Eppendorf tube containing 250 µL PBS. By tapping a few times on the Eppendorf tube, the fish larvae were rinsed, and then the PBS was removed with a Pasteur pipette. Immediately after, 50 µL of PBS was added into the Eppendorf tube and the contained larvae were smashed with the Micro-Vial Homogenizer System (Wilmad LabGlass Motor & Adapter, BP-7005-000, by ATS Life Sciences Wilmad, Vineland, NJ, USA) using sterilized pestles. The obtained extract was immediately centrifuged at 7000 rpm at 4 °C.

The supernatant was pipetted into an ATP test tube (Hygiena, UltraSnap, No.US 2020), and the ATP was measured using a luminometer (Hygiena, EnSURE V.2). The results were converted from RLU to pg ATP using the following formula: m = (RLU × 0.507)/2.

## 3. Results

### 3.1. Comprehensive Analysis of the Phytonutrient Profiles of GTEs and Their Putative Health-Promoting Effects

**Figure 1:**
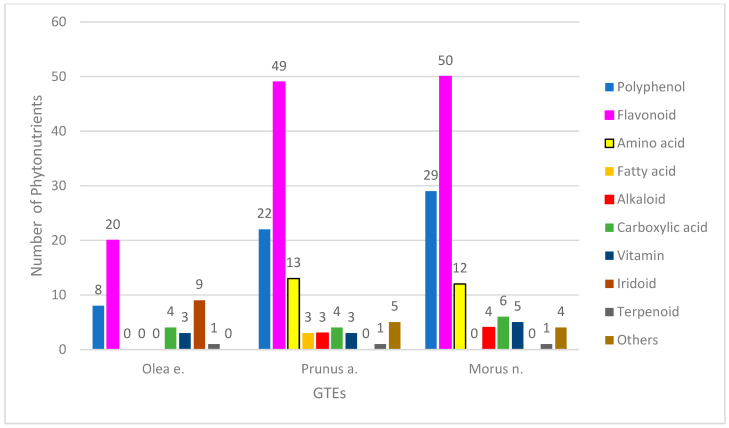
Phytonutrient profiles of GTEs.

The phytonutrient profiles of the GTEs were determined through a UHPLC–ESI-MS qualitative analysis. We were able to identify 45 chemical constituents in the olive GTE (O), 103 in the sweet almond GTW (SA), and 111 in the black mulberry GTE (BM), that corresponded to the following bioactive compound categories: polyphenols (hereafter, this refers only to the non-flavonoid compounds), flavonoids, coumarins, iridoids, alkaloids, amino or carboxylic acids, lignans, terpenoids, and vitamins (Figure 1).

Regarding the O-GTE profile—i.e., olive GTE—the highest numbers of compounds were observed to be flavonoids and iridoids, while the non-flavonoid polyphenols, coumarins, carboxylic acids, and vitamins represented much fewer chemical compounds (for detailed analyses, see Supplementary Materials Table S2). The alkaloid and terpenoid classes contained one single representative. The polyphenol content of olive oils was extensively studied, and, from the eight phenolics (tyrosyl, hydroxytyrosyl (HT), oleuropein, pinoresinol, caffeic, ferulic, vanillic, and p-coumaric acid) that were previously described [20], in our O-GTE, we could confirm only the presence of HT and oleuropein. It was previously shown by other research groups that, in some adipocytes, the HT could stimulate biosynthesis of mitochondria and increased expression of the electron transport chain complexes, including ATP synthase. Furthermore, it triggered the 5′AMP-activated protein kinase (AMPK) activity, increasing fatty acid oxidation so that such generated effects could explain the HT role in preventing diabetes mellitus [21]. The oleuropein confers stability to olive oils, and features anti-inflammatory and anti-cancer properties [22].

Other polyphenols like chlorogenic acid and verbascoside were also detected in our O-GTE analysis, and these have been shown in the literature to exert antioxidant, anti-inflammatory, and neuroprotective properties, together with beneficial effects on carbohydrate and lipid metabolism [23,24]. The O-GTE contained coumarins such as esculetin, scopoletin, and dihydroxycoumarin, that, when studied individually, showed antioxidant, anti-inflammatory, anticancer, antidiabetic, and cardiovascular protective pharmacological effects [25]. Kynurenic acid, a carboxylic acid determined in the O-GTE, was demonstrated to be an important kynurenine pathway metabolite with anti-neuroinflammatory and anti-cardiomyopathy features, which has proven necessary to maintain normal brain and cardiac function [26,27]. The 12-hydroxyjasmonic acid, another carboxylic acid found in the O-GTE, is a plant hormone involved in defense against pathogens [28], while the ginkgolic acid, another O-GTE component, was shown to inhibit both in vitro and in vivo SUMOylation, meaning it might have anti-cancer and anti-neurodegenerative effects [29]. The iridoids (oleoside, neonuzhenide, nuzhenide, oleuropein, lingstoride) form an important and specific phytonutrient category in the case of the O-GTE, since their neuroprotective, hepatoprotective, anti-inflammatory, antitumor, hypoglycemic, and hypolipidemic implication have all been demonstrated [30]. Among vitamins detected in the O-GTE, nicotinic acid (B3) and nicotinamide adenine are relevant for three coenzymes: nicotinamide adenine dinucleotide (NAD^+^), a reduced form of NAD^+^ (NADH), and flavin adenine dinucleotide (FAD), all implicated in ATP synthesis, redox potential, and inflammation regulation [31]. 

Interestingly, in the O-GTE, the flavonoids include about 20 phytonutrients such as quercetin, taxifolin, katuranin, luteolin, isoquercetin, rutin, cosmosiin, ligstroside, naringenin, apigenin, isorhamnetin, chrysoeriol, and pinocembrin (for detailed analyses, see Supplementary Materials Table S2). The presence of iridoids and flavonoids in young and mature olive leaf extracts were compared, and differences indicated that oleuropein and flavonoid aglycones were exceling in young leaves, while the glucosylated forms of luteolin were more prevalent in the mature leaves [32]. Our study on the young shoots denoted as O-GTE corroborates the previous observations, since the presence of oleuropein and many quercetin derivates could be confirmed in addition to the six glycosylated forms of luteolin (for detailed analyses, see Supplementary Materials Table S2). Two lignans and a terpenoid were also identified in the O-GTE, and both of them were suggested in the literature to have antioxidant, antitumor, anti-coronary heart disease, and estrogenic/antiestrogenic properties [33,34]. Taken together, our data strongly suggest the complexity of the phytonutrient profile of the O-GTE, comprising several compounds with many potentially health-promoting effects. 

Sweet almond (SA) seeds are considered to be an excellent source of nutrients, containing about 50% lipids, 25% proteins, 20% carbohydrates, and many phytonutrients with health-promoting effects, especially against the cardio-metabolic conditions [35]. Similar to the SA seeds, the SA-GTE, as revealed by the UPLC–ESI-MS analysis, contained a relatively high number of phytonutrients such as polyphenols (including both flavonoids and non-flavonoids), amino acids, carboxylic acids, and fatty acids (Figure 1) (for detailed analyses, see Supplementary Materials Table S3). The flavonoid class contained 49 compounds and seemed to be the most relevant, based on the high number of phytonutrient representatives. Among flavonoids, there were flavanols (quercetin, kaempferol, myricetin, narcissin, isorhamnetin), flavones (triacetin), and flavanones (naringenin, eriodyctiol), while, among the non-flavonoid polyphenols, there were hydroxycinnamic acids (chlorogenic acid, caffeic acid, coumaric acid, ferulic acid). Among the SA-GTE-specific carboxylic acids, the malic and citric acids are important metabolites, while the presence of quinic acid holds promise, as it was previously demonstrated to facilitate the synthesis of tryptophan and nicotinamide in the gastrointestinal tract, enhancing DNA repair and exhibiting anti-inflammatory effects [36]. The SA-GTE also contained 8 essential and 5 non-essential amino acids. Among the SA-GTE-identified alkaloids, choline is an essential nutrient with an important function in phospholipid, neurotransmitter (acetylcholine), and trimethylglycine synthesis, thus taking part in several important physiological processes [37]. Chelidonine, another alkaloid found in the SA-GTE, was shown to possess pro-apoptotic and anti-metastatic properties [38], while the third alkaloid, berberine, was demonstrated to reduce inflammation and insulin resistance by beneficially affecting glucose–lipid metabolism [39]. The vitamins detected in the SA-GTE were identical to those identified in case of O-GTE. The presence of fatty acids such as α-linoleic (ALA, *omega*-3 PUFA), linoleic (LA, *omega*-6, PUFA), and palmitoleic acids (*omega*-7 MUFA) in the SA-GTE are surprising, as we could not identify them in any of the other analyzed GTEs. LA and ALA were shown to have neuroprotective and neuroplastic properties in animal models for cardio- and neurodegenerative conditions [40,41,42]. LA can be used for the biosynthesis of prostaglandins and cellular membranes. The cis- and trans-isomers of the palmitoleic acid can have contrasting physiological effects such as downregulation of muscle-specific genes during myoblast differentiation, or antioxidant/anti-inflammatory properties [43]. Furthermore, in SA-GTE, we could detect 2-oxindole, known to readily react with electrophiles, while its derivatives would have many pharmacological activities [44]. The aldehyde indole-4-carbaldehyde, found in the SA-GTE, was also identified in the brown algae species *Sargassum thunbergii* and was shown to inhibit adipogenesis through the activation of the AMPK pathway [45]. Lastly, the identification of eugenol in the SA-GTE is puzzling, since, despite its antimicrobial and anti-inflammatory properties, there have also been some concerns about its toxicity [46]. We have also identified benzyl-glucoside and benzyl-primeveroside in SA-GTE; their occurrence in some other species of the *Prunus* genus was demonstrated earlier [47].

The BM-GTE phytonutrient profile, as revealed by UHPLC–ESI-MS, featured both non-flavonoid and flavonoid polyphenols—most numerous of the compounds identified—followed, in descending order, by amino and carboxylic acids, vitamins, alkaloids, and some miscellaneous compounds (Figure 1; for detailed analyses, see Supplementary Materials Table S4). Interestingly, amongst the 50 flavonoids detected, there were flavanols (quercetin, kaempferol, myricetin, narcissin, isorhamnetin), flavones (luteolin, apigenin triacetin), flavanones (naringenin, eriodyctiol), and isoflavones. Among the 29 non-flavonoid polyphenols, there were hydroxybenzoic acids, hydroxycinnamic acids (chlorogenic acid, caffeic acid, coumaric acid, ferulic acid), and stilbenes (resveratrol). The presence of albafuran A/B of 2-arylbenzofuran flavonoids, featuring antifungal properties, was confirmed for BM-GTE in our study [48]. Similarly, another benzofuran, chalcomoracin, has also been identified in BM-GTE, and was shown in the literature to possess antibacterial and anti-cancer properties [49,50]. Other 2-arylbenzofuran flavonoid derivates detected were mornigrol E and F. We could also demonstrate, in BM-GTE, the occurrence of flavonoids such as kuwanon A, B, C, E, and G that were found initially in the white mulberry (*Morus alba*) leaves, and were afterwards shown to possess multiple health-promoting benefits, including anti-inflammatory and anti-cancer properties [51,52,53,54]. Our study has also revealed the presence of 12 amino acids in the BM-GTE, of which lysine, arginine, threonine, isoleucine, leucine, phenylalanine, and tryptophan are essential, while proline, asparagine, aspartic acid, and tyrosine are non-essential (for detailed analyses, see Supplementary Materials Table S4). The presence of the amino acid citrulline in BM-GTE is highly relevant, knowing that its supplementation could augment arginine bioavailability, nitric oxide production, exercise performance, and recovery [55]. Among the carboxylic acids found in BM-GTE, malic, citric, and quinic acids were already reported for the SA-GTE. However, carboxylic compounds such as jasmonic, hydroxyjasmonic, and tuberonic acids are specific to the BM-GTE, and they are all related, as they are odoriferous components of plant-based essential oils. All vitamins seen in the BM-GTE correspond to B2-5 types, and, together with the chelidonine alkaloid, they have been detected in the case of the SA-GTE analysis. The other BM-GTE-specific alkaloids, 1-deoxynojirimycin (moranoline) and *O*-hexosyl-1-deoxynojirimycin, are considered alpha-glucosidase inhibitors, having antidiabetic effects [56]. In addition, a combination of 1-deoxynojirimycin and 1,4-dideoxy-1,4-imino-d-arabinitol (another alkaloid found in the BM-GTE) was proven to inhibit glycogenolysis in the hippocampal region, so that the astrocyte glycogen reserves are preserved; this could be beneficial for delaying or preventing depression [57]. Amongst the miscellaneous compounds identified in the BM-GTE, ethyl gallate is a plant metabolite with antioxidant, anti-diabetic, anti-obesity, anti-inflammatory, and antiviral effects [58,59,60]. The presence of lumichrome in the BM-GTE is supposed to arise from the photolysis of riboflavin, while the latter has also been detected in the assessed plant extract. In addition to SA-GTE, the presence of eugenol has been confirmed in the BM-GTE; therefore, its toxic effects should also be considered. 

### 3.2. Comparative Analysis of GTEs Related TPC and TFC

Polyphenols and flavonoids are considered important phytonutrients that generate many health-promoting effects. Therefore, we determined the TPC and TFC content of GTEs.

Our data indicate that the SA-GTE is the richest source for polyphenols, since it contains approximately twice and three times more polyphenols than the BM-GTE and O-GTE, as revealed by the TPC analysis (Table 4). 

Similarly, in the case of TFC (measured in mg RE/mL units), SA-GTE exceeds the flavonoid content of the O-GTE by 2.5 times and of the BM-GTE by 3.6 times. However, regarding the proportion of flavonoids among the polyphenols found in the O-GTE and SA-GTE, the values were 86% and 73.7%, respectively, meaning that both GTEs could also be considered an outstanding source of flavonoids. The flavonoids of the BM-GTE represent about 40% of polyphenols, indicating that non-flavonoid polyphenols represent significant constituents of the GTE. Thus, the TPC and TFC data show some similarity between the O-GTE and SA-GTE, while the BM-GTE seems to feature a more balanced polyphenol content. Altogether, the studied GTEs are valuable sources of flavonoids and polyphenols.

### 3.3. Assessment of the Quantitative Polyphenol Profile of GTEs

The analysis of the phytonutrient profile of the GTEs reveals that they contain a relatively high variety of polyphenolic compounds (Figure 1) and, therefore, we selected a set of well-established antioxidant and anti-inflammatory compounds for quantitative assessment (Table 1 and Table 2). Among the proposed polyphenols for quantification in the GTEs, two of them were of non-flavonoids (caffeic and chlorogenic acids), and ten were flavonoids (apigenin, chrysin, hyperoside, kaempferol, luteolin, luteolin-*7*-*O*-glucosid, naringenin, quercetin, rutoside or rutin, and vitexin). 

The quantitative analysis of the selected polyphenols of the O-GTE is presented in Table 5 (for detailed analyses, see Supplementary Materials Figures S30–S39—chromatograms and Figures S40–S49—MS spectra).

The data clearly indicate that, from all the assessed polyphenols, flavonoids such as luteolin-7-*O*-glucosid predominantly outweigh the others, followed, in decreasing order, by rutoside, hyperoside, chrysin, quercetin, apigenin, luteolin, vitexin, and naringenin. Non-flavonoids are represented by chlorogenic acid, while the absence of caffeic acid from the O-GTE was confirmed through the standard authentication and the UPLC–ESI-MS chromatogram analysis. 

The quantitative analysis of the selected polyphenols of the SA-GTE is presented in Table 5 (for detailed analyses, see Supplementary Materials Figures S50–S57—chromatograms and Figures S58–S65—MS spectra).

The obtained data reveal that, among all the assessed polyphenols, flavonoids such as rutoside and hyperoside overshadow the others (having a representation of about 90%), though non-flavonoids such as chlorogenic and caffeic acid are also present but to a lower extent. Chrysin, kaempherol, anrigenin, and apigenin are also present at lower concentrations. Flavonoids such as luteolin, luteolin-7-*O*-glucosid, and apigenin could not be detected in both types of analyses conducted on the SA-GTE employing HPLC–MS determinations.

The quantitative analysis of the selected polyphenols of the BM-GTE is presented in Table 5 (for detailed analyses, see Supplementary Materials Figures S66–S73—chromatograms and Figures S74–S81—MS spectra).

It is likely that chlorogenic acid and rutoside are the two most abundant polyphenols in the case of BM-GTE, while others such as hyperoside, apigenin, and chrysin are less significantly represented quantitatively. Polyphenols such as luteolin-7-*O*-glucoside, naringenin, and luteolin are barely traceable.

Taken together, the assessed polyphenols show GTE-specific profiles, despite some similarities among them being observable. Moreover, the metabolically mediated antidiabetic and anti-inflammatory effects of the selected polyphenols seem to be a shared feature of the evaluated olive, sweet almond, and black mulberry GTEs. 

The increased TPC and TFC values seen in in case of olive GTE were expected, since, for olive oil, it was already demonstrated that it contains 50 to 1000 mg/kg polyphenols in extracts from different plant parts [20,22].

### 3.4. Assessment of the GTE-Specific Antioxidant Capacity

It is well documented that plant-derived polyphenols can exert antioxidant properties so that they protect against oxidative stress-triggered/-associated inflammation and illnesses [61]. Therefore, testing the antioxidant potential of the plant-specific GTEs could shed light on their free radical scavenging or formation inhibition properties that might explain some of their health benefits [62]. Several methods can be applied in order to assess the antioxidant potential [63], so we used the DPPH, FRAP, and xanthine oxidase inhibition methods (see Materials and Methods), and the obtained results are shown in Table 6. The DPPH acts like a hydrogen radical scavenger, and its reduction level denotes the antioxidant potential of a given GTE. The half-maximal inhibitory concentration (IC50) shows how much GTP is needed to inhibit the DPPH antioxidant activity by half. Therefore, the O-GTE seems to be two times more efficient as an antioxidant compared to the SA-GTE, while, against the BM-GTE, the difference is of an approximate magnitude of three. Furthermore, the BM-GTE looks like the least proficient antioxidant for DPPH when compared to both the SA-GTE and O-GTE.

The FRAP method indicates the reduction power of a ferric to ferrous complex by the antioxidants from the GTE, and this time the O-GTE exerted an exceedingly elevated antioxidant potential as compared to the SA- and BM-GTEs, while the latter looked similar.

Xanthine oxidase catalyzes the hydroxylation of hypoxanthine to xanthine and uric acid, leading to the production of free radicals and the development of hyperuricemia, an eventual medical condition with several associated diseases [64]. Our data indicate the reduced inhibition of xanthine oxidase for all three GTEs, which suggests a reduced antioxidant effect in the context of the regulation of uric acid homeostasis or hyperuricemia-related diseases.

### 3.5. The Nutritive Profile Analysis of GTEs

In order to gain information on the nutritive potential of the analyzed GTEs, their total protein and carbohydrate contents were evaluated (see Section 2—Materials and Methods). The results are shown in Table 7, and indicate that the total protein content of all GTEs seems to be around 1 mg/mL.

Regarding the total protein content, the O- and BM-GTEs have a greater protein content, whereas the SA-GTE contained less protein. The richest carbohydrate level was observed in the O-GTE, which was shortly followed by the SA-GTE, and the BM-GTE displayed the lowest carbohydrate level. No relevant lipid accumulations could be observed in the three GTEs, whereas the presence of protein and carbohydrate types of macronutrients were evident, suggesting that the assessed GTEs might be able to feature additional nutritive effects. 

### 3.6. Assessment of the GTEs Specific Nutritive Properties Using the Drosophila melanogaster-Based Viability Tests at 0N Dietary Condition 

*Drosophila melanogaster* is a versatile model system that is suitable to question the genomic and nutritional dependence of many life-related parameters [65]. In this respect, we have analyzed specific dietary conditions (like 0N, NM, and HS) in combination with the GTEs in question, and individually assessed their effects on the survival of the larvae and the hatching of pupae in the context of the fruit fly lifecycle. We must admit that the nutritional requirements of *Drosophila melanogaster* were intensively studied previously, and, as an example, omitting only a single essential amino acid like arginine or isoleucine shortens lifespan by 30–70% [66]. Therefore, the careful evaluation of individuals obtaining the third instar larval (larval survival rate) and pupal stages (adult hatching rate), including the duration of these developmental stages, can be envisioned as indicators of the nutritional competencies of the applied diet. 

**Figure 2:**
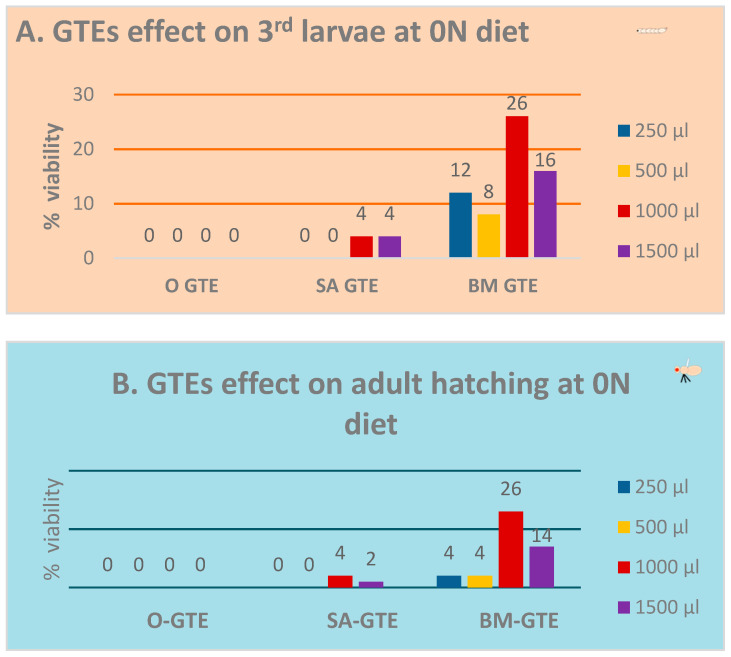
Evaluation of fruit fly viability in the 0N diet. Viability assessment of the larvae (**A**) and pupae (**B**) at the applied concentrations of GTEs.

In the context of the 0N (zero nutrient) diet being supplemented with a given GTE, all the relevant macro- and micronutrients were provided by means of the plant material used to obtain the corresponding GTE. Additionally, increasing amounts of individual GTEs were incorporated into the 0N diet to assess an eventual concentration range regarding the putative nutritive effects. The relevant data of the above-described experiment are shown in Figure 2A,B. Our observations indicate that the O-GTE did not support larval nor pupal development in the 0N diet. Contrary to the O-GTE, both SA- and BM-GTEs were able to promote the viability of larvae and adults to different extents. The SA-GTE generated a relatively reduced viability at around 2–4%, while the BM-GTE enhanced substantially, up to 26%, the survival of larvae and adults in the context of the applied concentrations.

**Figure 3:**
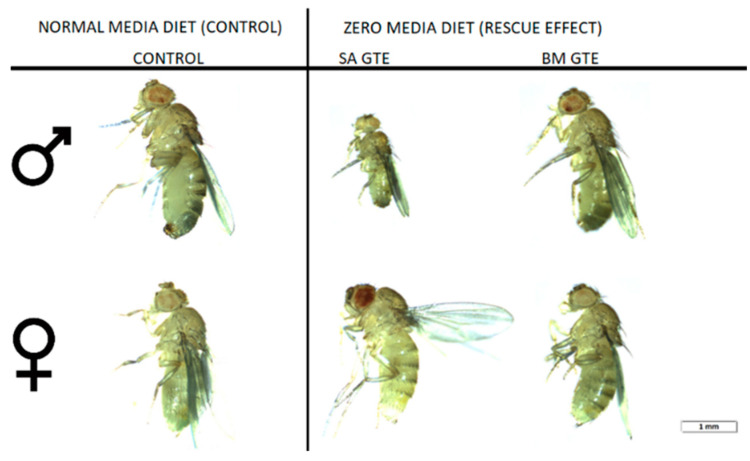
Images of w^m4h^ newly hatched adults raised at NM (control) and 0 M dietary conditions.

**Figure 4:**
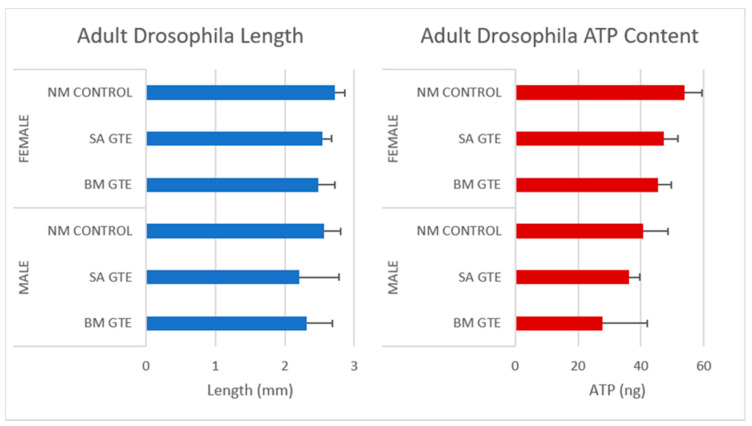
Assessment of w^m4h^ newly hatched adults for their body length (blue) and ATP content (red), raised in NM (control) and 0 M dietary conditions.

Remarkably, when we looked at the body size and ATP content of the SA- and BM-GTE-rescued w^m4h^ genotype and newly hatched (maximum 1-day-old) adult female and male individuals that were obtained with the 0M diet, some relevant differences were observed as compared to the NM diet-raised individuals (Figure 3 and Figure 4). In the case of *Drosophila melanogaster*, the sexual dimorphism featured some specificities like the lengthier size of females in comparison to the males. 

Regarding the body length, both GTEs could generate similar female sizes and had high resemblance in their ATP content. The male sizes and ATP content showed higher degree of variability, which could be due to the limited number of assessed individuals, but it is also possible that the two GTEs could have chemical composition-based dissimilarities that might differently affect the fruit flies’ growth and ATP-generating competencies.

Taken together, the concentration-dependent viability enhancement observed in the case of SA- and BM-GTEs would indicate that, in addition to the identified macronutrients, other components of the plant extracts like, for instance, the free amino acids might be implicated in the generation of the nutritive effects seen for both the SA and BM extracts. However, the observed SA- and BM-GTE-specific nutritive effects are of different strengths, with the BM-GTE being more proficient than the SA-GTE in covering the nutritional costs of *Drosophila melanogaster* development.

### 3.7. The HS Dietary Condition Delays the Development of w^m4h^ Drosophila melanogaster Strain without Affecting Viability

**Figure 5:**
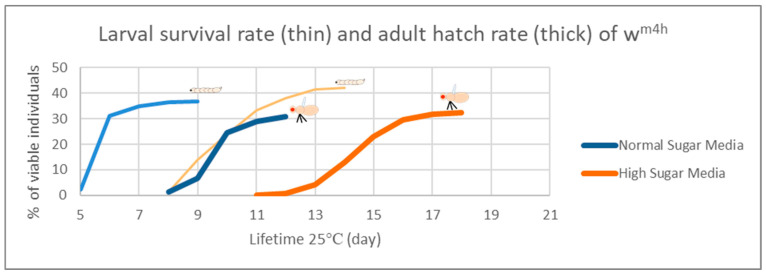
Developmental timing of fruit fly lifecycle on NS versus HS diet. The viability assessment of the larvae and pupae (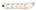) respectively adults (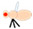) in the context of the duration of development in NM- and HS-dietary conditions. The blue curves are for normal sugar media respectively the orange one for high sugar media.

In the case of the *Drosophila melanogaster*, the HS diets have been shown to induce developmental delays during the life cycle. Therefore, in order to analyze the effects of our HS diet (1.5 M sucrose content) on the viability of third larvae and adult hatching, we also assessed the developmental timing of the studied w^m4h^ strain at 25 °C (Figure 5).

**Figure 6:**
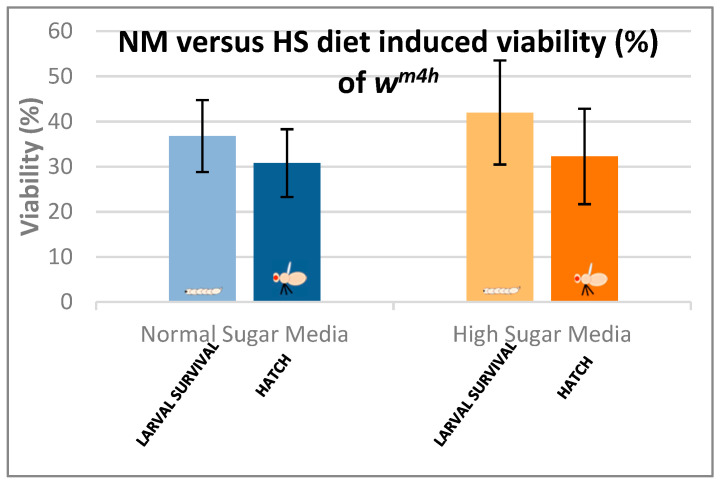
Comparison of larval and adult viability in NM- and HS-dietary conditions.

Figure 5 clearly indicates that our HS-diet induces a developmental delay of about 3 days with respect to the initiation and completion of third larval stage and the adult hatching. It is also interesting that, despite the HS-diet generated developmental delay, only the viability of third larval stage showed a slightly increasing tendency, whereas the adult hatching rate appeared almost identical to the control type of NM-dietary condition (Figure 6). 

These observations suggest that the currently applied HS-diet itself does not exert any substantial lethal effect upon the tested fruit fly strain so that some compensatory mechanisms should eventually overcome the increased carbohydrate intake-induced developmental delay mentioned above.

### 3.8. Evaluation of the Drosophila melanogaster Viability in NM- and HS-Diet Supplemented with GTEs

Next, the GTE-induced viability effects were assessed in combination with the NM- and HS-diets at 25 °C (see Section 2—Materials and Methods), knowing that the HS dietary condition results in some kind of diabetic status [67,68].

**Figure 7:**
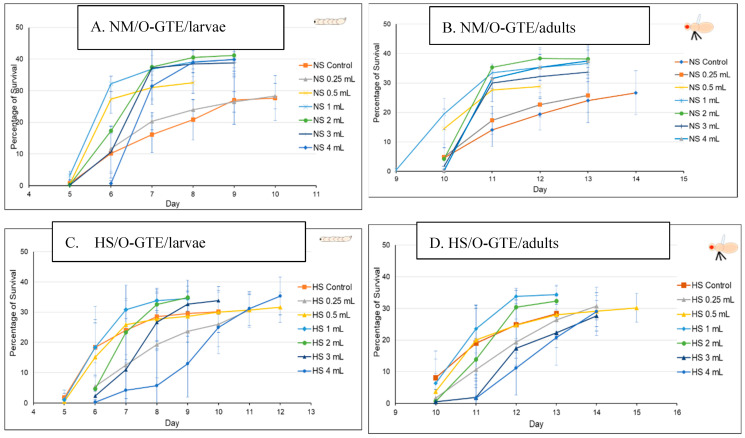
The fruit flies’ viability in NM- and HS-dietary conditions with O-GTE. Where NM means normal media and HS high sugar media.

The O-GTE specific data are shown in Figure 7A–D. All these figures are composed of two graphs, where (A) indicates the pupariation time of the third larvae or the third larval survival, whereas (B) indicates the hatching time of the adults from the pupae, which we would refer to as the adult survival. When the experiments were performed in NM in combination with increasing concentration of O-GTE, we could clearly observe that the survival rates of larvae and adults improved as the concentration of the GTE was raised from about 50% to approximately 80% (Figure 7A,B). The observed viability rise was associated with shorter larval and pupal developmental stages, which would indicate that the O-GTE exerts a significant fortification effect on the viability of fruit flies in normal dietary conditions. 

However, when the NM-diet was replaced with the HS, the O-GTE could increase the viability to about 60% in the case of the third larvae and adults when it was administered at lower concentrations; the high O-GTE levels seemed to increase the developmental delay without enhancing viability (see Figure 7C,D). It should be noted that 0.5 mL of O-GTE appeared to be a concentration that would be almost identical with the control, while 1–2 mL of O-GTE could increase viability without developmental delay. Furthermore, 3–4 mL of O-GTE produced a relevant (approximatively 2 days) larval and pupal delay, while the viability ultimately reached the control levels. Taken together, the fact that the O-GTE increased viability in both NM- and HS-type dietary conditions suggests that the olive gemmotherapy extract must contain important phytonutrients facilitating normal development and could certainly overcome some of the HS-diet associated restrictive effects.

**Figure 8:**
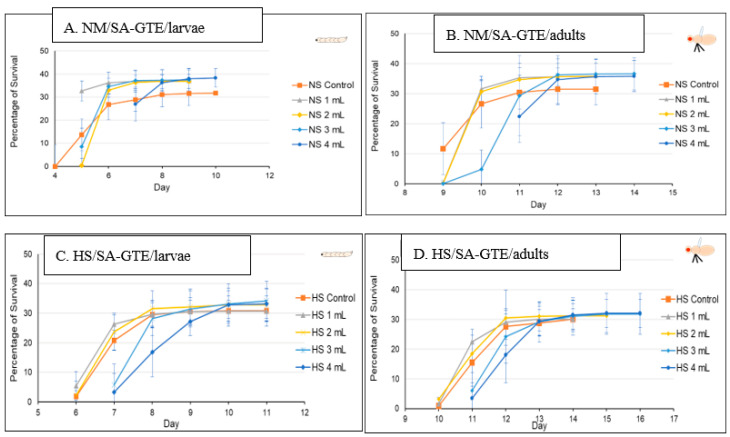
The fruit flies’ viability in NM- and HS-dietary conditions with SA-GTE.

In order to study the putative viability-generating activity of the SA-GTE, we performed the same set of experiments as in the case of the O-GTE. On the NM-diet, the SA-GTE increased the viability of w^m4h^ fruit flies at both the third larval and the adult stages, but no significant concentration-dependent effect was visible among the applied gemmotherapy extract concentrations (Figure 8A,B).

When the experiments with the SA-GTE were performed in the context of the HS-diet, a concentration-dependent antagonistic effect was apparent, with the lower concentrations facilitating faster development; though the higher quantities of SA-GTE seemed to delay larval and pupal stages, neither of them distressed viability, which eventually reached identical levels to the control (Figure 8C,D). Therefore, the SA-GTE did not have much of an enhancing effect on the larval and adult viability in the case of the HS-dietary condition. 

**Figure 9:**
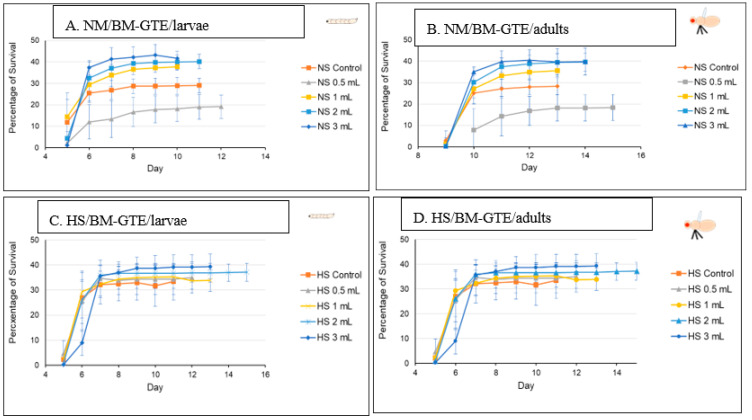
The fruit flies’ viability in NM- and HS-dietary conditions with BM-GTE.

Following the O- and SA-GTEs, and again applying a similar experimental set up, we looked at the BM-GTE-generated effects with respect to fruit fly viability. In NM-diet, we observed that the lowest concentration of BM-GTE significantly inhibited the viability of both larvae and adults as compared to control (Figure 9A,B). Interestingly, at higher BM-GTE concentrations, the augmented survival effects on larvae and adults were evident, suggesting that some phytonutrient(s) must exceed critical concentration(s) in order to efficiently promote viability. 

Moreover, when the above experiments were performed with the HS-diet, all the applied BM-GTE concentrations resulted in enhanced viability, even including the aforementioned inhibitory concentration in the NM-dietary condition (Figure 9C,D). This improved viability generated through the BM-GTE can be envisioned as indirect evidence for the recue effect in the HS-diet-induced Drosophila-specific diabetic condition. Taken together, the above-described GTE-based experiments denote particular concentration-dependent fortification and/or rescue effects regarding the viability of larval and adult fruit flies of the w^m4h^ genotype in NM- and HS-diet conditions, respectively.

### 3.9. Assessing the GTE-Associated Nutritional Effect Using the Carp (Cyprinus carpio) Larvae Model

**Figure 10:**
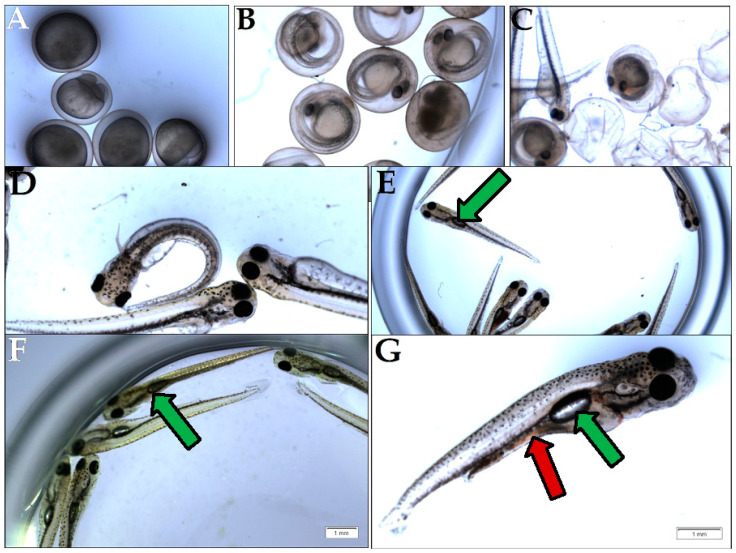
Assessment of carp viability. (**A**) Fertilized eggs during embryogenesis; (**B**) Eggs that completed embryogenesis with visible larvae; (**C**) Larvae at the time of hatching; (**D**) Non-feeding larvae at day 1 after hatching, also called pre-feeding larvae; (**E**) Feeding larvae, day 3 after hatching (green arrow indicates the swim bladder); (**F**) Feeding larvae at day 7 after hatching, fed with GTE; (**G**) Feeding larva at day 7, fed with brine shrimp (red arrow).

In general terms, animal nutrition is seen as an intervention that is meant to supply nutrients to organisms, in order to efficiently produce ATP and cover the energetic needs of life events. Seeing that the studied GTEs are able to differentially influence the viability of the fruit fly, from larval stages onwards, as soon as food intake begins, we decided to assess the GTEs in question using a carp (*Cyprinus carpio*)-based model system. After propagation in the case of the carp species, the embryogenesis proceeds until larvae develop that hatch eventually (Figure 10). The newly hatched larva initially does not feed itself, while, on the second or third day after hatching (depending on the temperature), it starts eating, and, usually, the most suitable fish feed is the living brine shrimp (*Artemia salina*) in artificial circumstances. The feeding larval period starting from the third day is critical because, if larval nutrition is affected, their growth might be restricted, and they would die. It is important to notice that, three days after hatching, the larvae fill their swim bladder with air and start feeding (Figure 10E).

**Figure 11:**
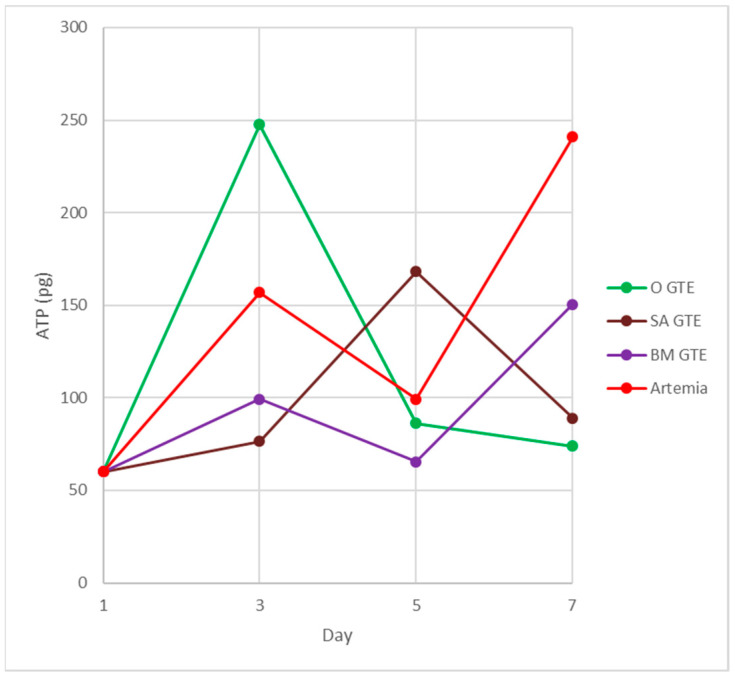
Fish feed-induced ATP synthesis in carp larvae. The colors indicate the fish feed: green—O-GTE; red—control, i.e., brine shrimp (*Artemia salina*); brown—SA-GTE; purple—BM-GTE.

This time point marks the beginning of larval metabolism-derived ATP that is produced at the expense of the consumed food and air-derived oxygen. Our experimental setup compared the brine shrimp and the studied GTE-associated nutritive potential by assessing the ATP yield and body length increment per larva (see Section 2—Materials and Methods). The obtained data indicated that, in the case of the fertilized eggs and the non-feeding larval stages, no obvious differences were observed among brine shrimp and GTE-fed individuals (see Table 8). Moreover, in the case of the feeding larvae 3 days after hatching, the sizes of larvae appeared similar, but obvious differences were evident among the ATP yields (see Table 8 and Figure 11).

At this developmental time point, the highest generated ATP levels were specific to the O-GTE, followed, in decreasing order, by the brine shrimp type of control feed, while the BM- and SA-types of GTEs proved substantially less efficient in generating ATP. It is also interesting that the O-GTE seemed to exceed ATP yield generation by more than 3 times as compared to the SA-GTE, while, compared to the BM-GTE, it featured a 2.5-fold increase and 1.5-fold rise, as compared to the brine shrimp (Figure 11). 

Assessing the feeding larvae 5 days after hatching revealed that the larval body length was almost unchanged (image not included in Figure 10), but some relevant modifications regarding the ATP content were evident (see Table 8 and Figure 11). Remarkably, the ATP content increased only for the SA-GTE, while decreasing ATP concentrations were observed, not just for the brine shrimp type of control, but also for the other two GTEs—olive and black mulberry. 

The analysis of the feeding larvae 7 days after hatching showed the body length of the three GTE-fed larvae were relatively similar and lagged behind the control values. It is also important to emphasize that, on day 7, the ATP concentration increased substantially in the brine shrimp-fed type of control larvae, and also a rising tendency yet diminished ATP content were detected for the BM-GTE-fed larvae. In the case of olive and sweet almond GTE-fed carp larvae, the ATP concentration decreased visibly at day 7, and, remarkably, on day 9 after larvae hatching, only the brine shrimp-fed carp larvae were alive. 

Our observations indicate that, initially, on day 3 after larval hatching, the O-GTE showed a greater ATP-generating potential than the control type of brine shrimp-fed larvae. Later, on day 5, the detected ATP increased only in the case of SA-GTE, while, on day 7, the rising ATP content was only evident for the BM-GTE, but this value represented just 60% of the control-specific ATP content. 

In order to test if the assessed GTEs would have nutritive properties in the case of the carp-specific hatched embryos, we replaced the brine shrimp type of live food with the assessed GTEs and measured the larvae-specific ATP levels (Figure 11).

## 4. Discussion

### 4.1. The Polyphenol Profiles of GTEs Show Some Resemblances and Similar Putative Health-Promoting Effects

We carried out both qualitative and quantitative chemical analyses of the studied GTEs in order to achieve more comprehensive characterizations regarding the composition of the studied plant extracts. Figure 1 shows that the major chemical compound categories identified in the GTEs are polyphenols (both flavonoids and non-flavonoids), coumarins, iridoids, alkaloids, amino or carboxylic acids, lignans, terpenoids, and vitamins. The performed UPLC–ESI-MS studies revealed 45 olive-, 103 sweet almond-, and 111 black mulberry-specific GTE phytoconstituents; for many, their presence is reported exclusively in the present study. 

**Figure 12:**
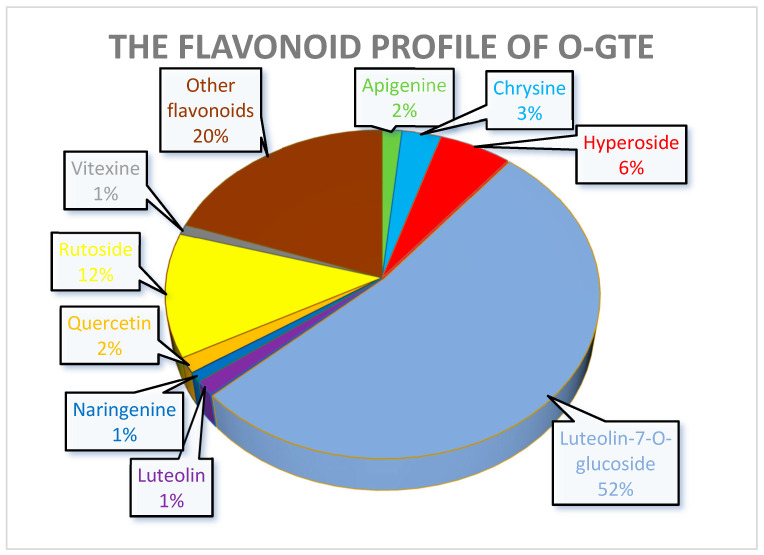
The quantitatively assessed selected polyphenol distribution in O-GTE.

The experiments that were meant to quantify some of the polyphenols in the GTEs also gave interesting insights and allowed us to predict some of the putative health benefits. In the case of the O-GTE, three polyphenols seem to have a significant representation (Figure 12). The increased quantity of luteolin-7-*O*-glucoside suggests that the O-GTE might possesses a substantial anti-inflammatory and anti-proliferative effect [69,70].

Moreover, rutoside, being the second-most abundant O-GTE flavonoid after luteolin-7-*O*-glucoside, was shown by others to feature not just anti-inflammatory, but also antidiabetic effects [71]. Chlorogenic acid being the third-most abundant is expected to substantiate the O-GTE-associated hypoglycemic, hypolipidemic, and anti-inflammatory properties [72,73].

**Figure 13:**
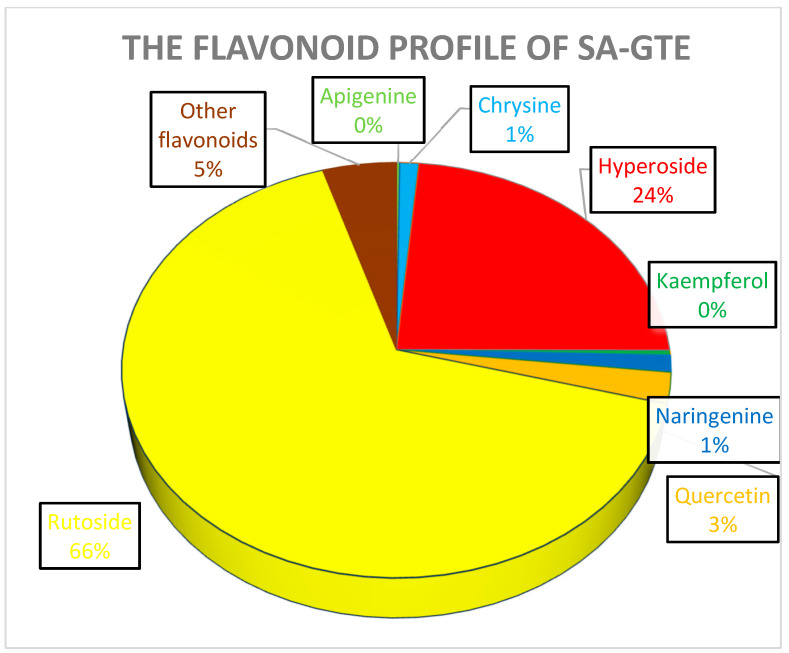
The quantitatively assessed selected polyphenol distribution in SA-GTE.

In the case of SM-GTE, the distribution of the quantified polyphenols is shown in Figure 13 It is worth noticing that the SA-GTE, with its higher rutoside and hyperoside content, is anticipated to possess significant anti-diabetic and anti-inflammatory properties, based on previous studies [74]. 

Rutoside and hyperoside are derivates of quercetin, which is also present at relatively small quantities in the SA-GTE, and all three might be involved in regulating metabolism and anti-inflammatory processes [75]. The substantial chlorogenic and caffeic acid contents are expected to further substantiate the antioxidant, anti-inflammatory, and metabolic regulatory effects of the SA-GTE, as suggested by other studies [76].

**Figure 14:**
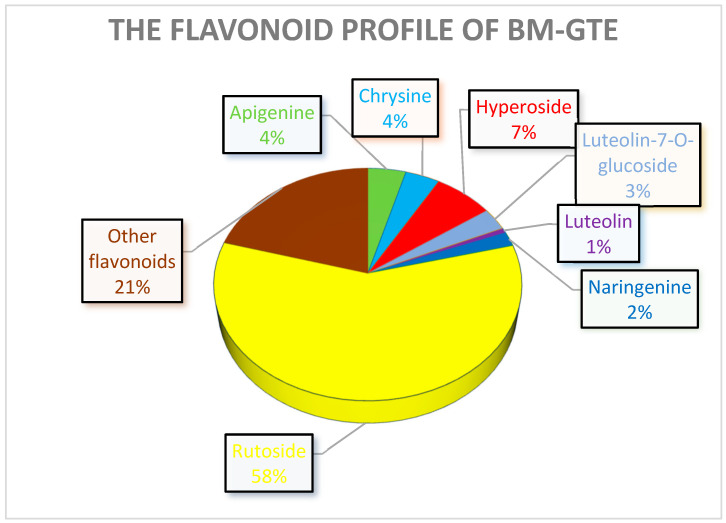
The quantitatively assessed selected polyphenol distribution in BM-GTE.

Quantitative analysis of the selected polyphenols for the BM-GTE showed high contents of chlorogenic acid and rutoside (Figure 14). Chlorogenic acid and rutoside have demonstrated their efficacy in protecting mice against hypoxic conditions [77]. Moreover, in the case of the leaf extract of *Morus alba* (a close relative of BM) being rich in chlorogenic acid and rutoside, it was shown to infer an anti-diabetic effect for type 2 diabetic rats [78]. Despite such beneficial effects, care should be taken, since rutoside and chlorogenic acid also feature slight synergism regarding the inhibition of thyroid peroxidase [79].

Chrysin and apigenin are relevant flavonoids found in BM-GTE, and their combination has been shown to feature anti-inflammatory [80] and antioxidant [81] properties. However, contrasting observations also demonstrated that especially chrysin showed cytotoxicity at very low (2 µM) concentrations [82].

Taken together, our observations indicate that all three GTEs showed significant rutoside contents, while the O-GTE further contained higher amounts of luteolin-7-*O*-glicoside, the SA-GTE had more hyperoside, and the BM-GTE had chlorogenic acid. The abovementioned polyphenol combinations could be used to specifically define quantitative markers for each of the studied GTEs, while, with respect to the predictable the anti-inflammatory and anti-hyperglycemic effects, they could present certain similarities

### 4.2. The Phytochemical Complexity of GTEs Pleads for Insightful Evaluation of the Generated Biological Effects

Interestingly, comparing the viability of the fruit fly larvae that were raised under NM- or HS-diets, we could observe some relevant differences, though the HS-diet seemed to induce about a 3-day delay in the developmental time (see Figure 5 and Figure 6). However, when such experiments included the GTEs, the concentration dependency of viability was evident to a variable extent, but the HS-diet-specific developmental delay was not affected. Nevertheless, we could observe that the O-GTE increased viability substantially under the NM- and, less intensively, over the mild concentration of HS-type diets (Figure 7). The BM-GTE featured a viability trend that was almost similar to the O-GTE, with the higher BM-GTE concentrations featuring a stronger rescue effect (Figure 9). Interestingly, the SA-GTE displayed a less pronounced viability increase over the NM-diet, while, in the case of the HS-diet, no obvious differences were apparent in comparison with the control (Figure 8). Taken together, the olive and black mulberry GTEs could exceed—while the SA-GTE did not significantly modify—the HS-diet-specific viability threshold, and they all could not compensate for the developmental delay. These observations suggest that the analyzed GTEs should have some phytonutrient profile specificities that might be accountable for the fluctuating viability upon specific dietary conditions. 

Our qualitative and quantitative analyses of the studied GTEs have revealed chemical compositions with plenty of similarities and differences. Some polyphenols like tyrosol, HT, oleuropein, pinoresinol, and iridoids were present only in the O-GTE, but fatty acids like omega 3, 6, and 7 were found exclusively in the SA-GTE. The BM-GTE phytonutrient profile showed resemblance to SA-GTE with the exception of some specific flavonoids like benzofurans, whose exclusivity to mulberry was evident. It was also interesting to notice that nicotinic acid and nicotinamide adenine were present in all the studied GTEs, suggesting that these phytonutrients might support the synthesis of ATP, redox, and inflammation regulation.

We analyzed the TPC and TFC of the GTEs, and then assessed the extracts’ antioxidant capacity in in vitro conditions. It turned out that the SA-GTE showed the highest TPC and TFC values, followed by the BM- and O-GTE-specific TPC values, while, for the TFC, the O-GTE slightly exceeded the BM-GTE (see Table 4). However, when we assessed the antioxidant capacity by the FRAP method, the O-GTE appeared to be the most considerable, and, though the SA-GTE looked greatly reduced, the latter was closely followed by the BM-GTE (see Table 9).

Similarly, the DPPH method indicated that the O-GTE had the highest in vitro antioxidant capacity, followed, in decreasing order, by the SA- and BM-GTEs. It should be mentioned that the FRAP method is based on an electron transfer-based assay, while the DPPH method implies an electron/hydrogen atom transfer-related mixed test, meaning that the two procedures are not fully equivalent [83]. Noticeably, the in vitro antioxidant capacity of the assessed GTEs with the two abovementioned methods showed a similar strength of pattern, with olive in the lead, the sweet almond at the intermediate position, and the black mulberry featuring the most reduced potential. Many studies carried out on plant extracts suggest that the in vitro antioxidant capacity could not be entirely correlated with the in vivo health-promoting effects. However, the TPC and TFC might offer relevant cues about the GTE-generated biological consequences upon consumers, since polyphenols have recently been attributed caloric restriction-mimetic (CRM)-type properties [84,85]. 

Caloric restriction (CR) induces a set of events like the depletion of the cytosolic acetyl coenzyme A (AcCoA), the inhibition of acetyltransferases, the stimulation of protein deacetylation, and the induction of autophagy, with all these being fairly specific and evolutionarily conserved mechanisms among species like yeast (*Saccharomyces cerevisiae*), nematodes (*Caenorhabditis elegans*), flies (*Drosophila melanogaster*), rodents (*Mus musculus*), and non-human primates (*Macaca mulatta*) [86]. Our previous observations suggested that, when the fruit fly larvae were subjected to the HS-diet, the expression of genes like the AcCoA-generating enzyme, the so-called *Acetyl Coenzyme A synthase* (*AcCoAS*), and the acetylation inducer *Acetyl-CoA acetyltransferase1* (*ACAT1*) were significantly increased. Moreover, the *sirtuin1* (an NAD^+^-dependent deacetylase involved in gene-silencing), the *histone deacetylase 4* and *1* (*HDAC4* and *1*), together with the *ATG8a* (inducer of autophagosome formation) were genes whose expression levels appeared greatly reduced when compared to the NM-diet fed controls (E. Máthé, unpublished results). These observations indicate that the HS-diet most likely increases acetylation through the augmented *AcCoAS* and *ACAT1* gene activities, while deacetylation is diminished through the repressed genes like *Sirt1* and *HDAC4* and *1*. Interestingly, it has been demonstrated by others that the reversal of the abovementioned gene activity is associated with the benefic effects of caloric restriction [87], and, moreover, some polyphenols are able to mimic the effects of caloric restriction [85]. Furthermore, polyphenols like resveratrol, quercetin, hydroxytyrosol, and myricetin—all identified in the studied GTEs—have been found by others to act like CRM, which can regulate mitochondrial biogenesis and mitophagy [88]. However, it remains an open question to elucidate if the studied GTEs would possess the competency for inducing caloric restriction-specific cellular mechanisms. 

Quite remarkably, the polyphenol and flavonoid profiles of the assessed GTEs were both qualitatively and quantitatively studied, and the most abundant polyphenol constituents identified in the O-GTE were luteolin-7-*O*-glucoside and rutoside, in the SA-GTE were rutoside and hyperoside, and in BM-GTE were chlorogenic acid and rutoside. Rutoside is the communal flavonoid for all three GTEs, and has been shown to induce an antihyperglycemic effect in rats based on an insulin-mimetic property by activating the insulin signaling pathway, implicating genes like phosphoinositol 3 kinase (PI3K), protein kinase C (PKC), peroxisome proliferator-activated receptor gamma (PPARγ), and glucose transporter (Glut) [71]. It is also quite astonishing that the fruit fly-specific HS-diet would reduce the expression of several genes like PI3K21B, PKC98C, Eip75B, Glut1, and Glut4EF, all being implicated in insulin signaling. Therefore, in order to assess the putative antidiabetic effect(s) of the GTEs, it seems logical to study the expression level of the abovementioned *Drosophila melanogaster* genes. 

Rutin is also known to maintain the intracellular NADPH level by inhibiting aldose reductase, which catalyzes the first step (conversion between alditol and aldose, using NADP as a cofactor) in the polyol pathway that transforms glucose into sorbitol. The increased production of osmotically active sorbitol is responsible for the development of secondary diabetic complications such as retinopathy, nephropathy, and neuropathy [89]. Remarkably, the HS-diet significantly augmented the expression of fruit fly genes like *Aldo-keto reductase 1B* (*Akr1B*), CG9436, CG10863, and CG12766, suggesting that the HS-diet-induced hyperglycemia might increase the flux of glucose via the polyol pathway, while the studied GTEs, through their rutin content, might be able to suppress the pathologically challenging sorbitol synthesis. It is also possible that the GTEs could increase the activity of the sorbitol dehydrogenases that are involved in sorbitol catabolism. Interestingly, *Drosophila melanogaster* has two sorbitol dehydrogenase genes (*Sodh1* and *2*), and their expression levels are increased, indicating that the possible conversion of sorbitol to fructose is active and the formation of advanced glycation end products (AGEs) is predictable. Furthermore, rutin, containing multiple -OH moieties, is expected to possess a relevant in vivo free radical scavenging potential, and, therefore, could reduce hyperglycemia- and neurodegeneration-induced oxidative stress and inflammation [90]. 

Seeing the rutoside (rutin, quercetin-3-rutinoside) health-promoting effects on different model species, and its possible implication in the generation of all three GTEs’ specific viabilities in relation to the HS-diet-subjected *Drosophila* w^m4h^ in vivo test system, these antecedents plead for an intriguing but complex scenario that implies multiple players. Indeed, the highest rutoside content was seen in the BM-GTE, and, presumably, the HS-diet-specific augmented fruit fly (both larvae and adult) viability could be related to this phytonutrient representation. However, the reduced in vitro antioxidant potential of the BM-GTE also suggests that the exerted antihyperglycemic effect, leading to a more pronounced viability, might imply other cellular mechanisms than just its direct involvement in oxidative stress reduction. It is also possible that the combination of chlorogenic acid with rutoside would result in a more pronounced hyperglycemic effect, and the chlorogenic acid involvement in inducing the Nrf2 signaling pathway is indeed known [91]. However, as expected in the fruit fly, the HS-diet slightly diminished the expression of *Keap1* and cap-a-collar (*cnc*; the Nrf2 homologue; E. Máthé, unpublished results) genes, suggesting an affected antioxidant signaling upon increased carbohydrate intake. It is also possible that, in the case of O-GTE, the combination of rutoside with luteolin-7-*O*-glucoside, and, in the case of SA-GTE, the rutoside and hyperoside pairing, would generate different types of cellular efficiencies and/or coping mechanisms in relation to the nutritional intake. Moreover, it is also possible that the GTE-specific polyphenol and flavonoid profiles can unleash multiple interactions based on synergistic, additive, and/or inhibitory effects in the cellular/organismal physiology, as it has been shown that the polyphenols interact with many cellular signaling events [62].

In this respect, phenomena like caloric restriction mimetics, insulin signaling, carbohydrate and lipid metabolisms, and polyol and Nrf2 pathways, together with immunity, must be all addressed, paying attention to the dietary setups in order to explain, in a more comprehensive way, the antidiabetic, anti-inflammatory, and neuroprotective outcomes of the GTEs’ applications. 

### 4.3. The AA Content of GTEs Might Interfere with the Larval and Pupal Viability of Drosophila melanogaster

The life stage-specific nutritional needs of *Drosophila melanogaster* have been addressed through several studies, and, for the larval stage, it is consistently accepted that the essential amino acids are the major growth-limiting factors with respect to diet manipulation [92,93]. Actually, it has been demonstrated that, in the holidic media (HM, regarded as the minimal dietary condition for complete development at 25 °C), the growth of larvae appears as a linear process under stable water content (85–89%), and lasts for 7 days. Interestingly, when the sucrose content is increased (a situation that resembles, to some extent, the currently applied HS-diet), the larval growth rate is not augmented, but when the essential amino acids’ (EAAs’) presence is doubled in the HM, concomitantly, the larval growth rate, together with the larval protein content, increases too. Some EAAs like arginine, histidine, isoleucine, leucine, lysine, methionine, phenylalanine, threonine, tryptophan, and valine were shown to adequately support fruit fly development, egg-laying of the adult females, and lifespan, all on the HM type of diet [66]. Undoubtedly, tryptophan is a very important EAA, as it is involved in the synthesis of proteins and methoxyindoles (serotonin and melatonin), and, through the kynurenine pathway, it regulates NAD^+^ synthesis [94], Zn and redox homeostasis [95], ageing, and lifespan [96]. Furthermore, among the EAAs, the absence of arginine or isoleucine seem to be detrimental, whereas glutamate could be a replacement for all other non-essential AAs. Moreover, the importance of AA bioavailability for the larval and pupal developmental stages has been further emphasized by several relevant developmental genetics studies [97,98,99].

The assessment of the O-, SA-, and BM-GTE-related effects on larval and pupal viability showed specific features with respect to the applied dietary conditions. In the case of the 0N-diet, the O-GTE sustained neither the larval nor the pupal developmental stages, and, as a consequence, no second or third instar larvae were observed crawling in the media at any of the applied extract concentrations (Figure 7A,B). In the case of SA-GTE at the highest concentrations, there were very few visible larvae and hatched individuals, suggesting a diminished viability-supporting effect of the 0N-diet. Astonishingly, the BM-GTE was able to overcome the nutrient-deficient feature of the 0N-diet, so that larval and pupal viability reached a maximum of about 26% at almost the highest extract concentration. It was also interesting to observe no relevant differences concerning the body length and ATP content of the emerged females if the corresponding larvae were raised under NM- or 0N-diet conditions supplemented with either SA- or BM-GTE (Figure 8 and Figure 9). In the case of males, the variability in body length and ATP content was more noticeable. These experiments, together with the seen rescue effects and, especially, the 0N-diet supplementations with the SA- or BM-GTEs, clearly indicate that both extracts contain some nutrients that support larval development. Looking at the phytonutrient profiles of the three assessed GTEs, it seems logical to consider the relevance of AAs and, especially, EAAs. The importance of AAs for the larval and pupal developmental stages of the fruit fly is relatively well-documented [92]; AAs circulating in the whole body, being detected by the fat body together with some neurons/glial cells from the brain, also control protein synthesis and metabolic regulation. Among the EAAs, leucine was shown to promote insulin release and larval growth in fruit flies [100,101], while tryptophan can be used for serotonin, and phenylalanine for dopamine-like neurotransmitter synthesis. Noteworthy among the EAAs, histidine and valine were missing from all the studied GTEs, while the SA-GTE was seen to not contain any tryptophan, and methionine was absent from the BM-GTE (Table 10). Noticeably, there were no EAAs detected in the O-GTE that could explain the inability of this gemmotherapy extract to support larval viability on the 0N-diet. On the other hand, the exclusive presence of tryptophan in the BM-GTE would suggest the implication of such an EAA in the larval/pupal viability of fruit flies. It is equally possible that, next to tryptophan, there might exist other contributing factors like the concentration of individual EAAs to support larval viability at 0N-diet but, due to the limitations of the applied methodologies, they might remain obscure. 

Nevertheless, when it comes to supplying or relocating resources for metabolism in order to produce ATP, the BM-GTE is missing histidine, methionine, and valine, which are of the glucogenic type of EAAs, while the existing leucine and lysine are of the ketogenic type, and isoleucine, phenylalanine, threonine, and tryptophan are of both the glucogenic and ketogenic types. Given this situation, it is highly predictable that the BM-GTE would be able to fuel the Krebs cycle from the pyruvate and acetyl-CoA, while threonine and isoleucine might use the succinyl-CoA as another entry point. It seems, therefore, likely that the BM-GTE, via pyruvate, acetyl-CoA, and succinyl-CoA, would be proficient in reallocating its AA resources in order to support larval/pupal development. Moreover, recently, it has been demonstrated that fruit fly larvae could sense the low-protein diet (as would be the case in our 0N-diet) via the NEAA tyrosine-specific induction of the ATF4 target genes in the fat body [102]. Surprisingly, tyrosine is necessary and sufficient to promote low-protein diet-specific adaptive responses like scaling down protein synthesis and increasing food intake. Therefore, the exclusive occurrence of the EAA tryptophan and an NEAA0like tyrosine from the BM-GTE could be envisioned like a minimal requirement for the larval/pupal viability of the w^m4h^ *Drosophila melanogaster* strain. Nevertheless, it remains an open question if tryptophan can act like a limiting EAA that could prevent protein synthesis in the larvae beyond its BM-GTE-specific concentration.

### 4.4. The Analyzed GTEs Feature Nutritive Properties 

Analysis of plant extract-associated and/or -generated physiological effects in animal organisms is becoming a very interesting and challenging task. Animal models can provide a plethora of valuable and invaluable information regarding the ever-going quest for medical knowledge and the improvement of interventions with respect to human diseases [103]. Undoubtedly, to overcome such limiting situations, different solutions could be envisioned, and we are proposing a translational model system based on two animal species: the fruit fly (*Drosophila melanogaster*), an insect, and the carp (*Cyprinus carpio*), a herbivorous fish. We have chosen to assess, in parallel, GTEs using these two species at their larval development stages because this particular period during their life cycle is predominantly sensitive to and highly dependent on nutrition. The currently reported experimental setup allowed the direct comparison of three GTEs, based on *Drosophila melanogaster* larval individuals with identical genotype and age, while they were further exposed to controlled dietary approaches. This kind of experiment allowed us to test the GTE-generated biological effects with respect to the so-called zero nutrient—0N (no nutrient), normal nutrient—NM, and high sugar—HS diets. The 0N-diet is a kind of negative control, which, if supplemented with a GTE, facilitates progression throughout the larval sages and pupariation, followed by the eventual hatching of adult individuals that, altogether, could be envisioned as direct evidence supporting the nutritive features of the analyzed plant extract. Furthermore, if the GTE increases viability of individuals in NM dietary conditions, that could be regarded again as direct evidence for increasing viability, whereas seeing a kind of rescue effect upon the HS-diet-specific viability or developmental delay might be considered an efficient adaptive stress response, implying anti-diabetic and/or anti-inflammatory mechanisms (Table 11). It is also important to pinpoint that, when significant and reproducible rescue effects are observed, and only then, it is worth engaging in more sophisticated studies to establish the genetic, molecular, and cellular features of the rescue mechanisms.

The assessment of the analyzed GTEs, alongside the NM-diet, clearly revealed their increased viability-related effects in the case of the O-, SA- and BM-GTEs. However, the concentration dependency of these effects was more pronounced in the case of the O- and BM-extracts, while the SA-GTE was less dependent. In the HS-diet, the O-GTE showed a highly variable concentration-dependent viability effect, while the SA-GTE featured variable but slightly increased viability. The BM-GTE showed the strongest viability increase, which was significantly concentration-dependent upon the HS-diet. It is important to admit that the abovementioned viability categories are based on subjective arbitrary considerations, and it would be highly speculative to invoke any regulatory mechanisms explaining the phenomenon. Nevertheless, the abovementioned experiments clearly indicates that the analyzed GTEs all possesses viability-increasing properties that could vary along a relatively wide concentration range. Moreover, the experiments performed on the *Cyprinus carpio* were based on the carefully staged larval assessment of the same GTE-associated nutritive properties. For the study, we recorded the larval length and ATP content, so that we were able to detect some increase with respect to the analyzed parameters. Again, these data suggest that the O-, SA-, and BM-GTEs, through their different nutrient contents, were able to feature some specific nutritive features. Therefore, by combining the fruit fly and carp species, a novel translational experimental model emerged that offers a more comprehensive view on the GTE-generated nutritive effects. We should also admit that no toxic or hormetic properties were identified. 

## 5. Conclusions

The current study excels in its comparative nature and describes the comprehensive analyses of the O-, SA- and BM-GTEs. The extracts’ preparation methodology and the phytonutrient profile survey, together with the GTE-specific viability assessments revealing nutritive features, were all performed in parallel using identical experimental conditions. Through the combination of the *Drosophila melanogaster*- and *Cyprinus carpio*-based larval viability evaluation, a nutritive assessment model system emerged that increased the confidence with respect to the generated experimental results. Moreover, in the case where similarities were detected despite the evolutionary distance between the abovementioned species, we tried to find the connection between the GTE-specific phytonutrient composition and the observed biological phenomenon. The studied GTEs reveal a complex qualitative and quantitative composition, and several new phytonutrients were identified that might back up some of the already described physiological effects associated with the corresponding plant species. The O-, SA-, and BM-GTE-related effects on larval and pupal viability are very much dependent on the applied dietary conditions (0N, NM, and HS), meaning that the GTE-generated effects should be assessed in a larger nutritional context. The EAA and NEAA content of GTEs are of nutritional importance and their relevance should be emphasized. By using NM dietary conditions, all three GTEs increased larval/pupal viability in different proportions, suggesting that their phytonutrient content could be relevant for the detected variability. In our study, TPC and TFC do not seem to fully correlate with the detected larval/pupal viability effects, but the selectively quantified polyphenol and flavonoid representatives could offer some valuable indications regarding the presumptive rescue mechanisms. By quantifying the ATP content of the fruit fly larva/adult and carp fish larva, we could demonstrate the GTE-associated ATP-generating potential, concluding that the BM-GTE appears the most efficient, while the O- and SA-GTEs can feature such a property, depending on dietary conditions.

Taken together, the results in the current paper could be envisioned as an attempt to compel a scenario in order to study the complexity of GTEs’ chemical compositions and their associated physiological effects. However, we also made an attempt to develop a translational model system based on fruit fly and carp species to be able to comprehensively study the nutritional relevance of GTE-generated health-promoting effects.

## Figures and Tables

**Table 1:** Standards used in the UHPLC–ESI-MS quantitative analysis of polyphenols of GTEs.

Name of Standard	Origin	Concentration Range, mg/mL	Calibration Curve Equation	Correlation Factor	Detection Limit, mg/mL	Quantification Limit, mg/mL
Caffeic acid	Phytolab, Vestenbergsgreuth, Germany	0.11–1.10	Area = 4 × 10^7^ × conc[mg/mL] − 319,689	0.9998	3.20	4.80
Chlorogenic acid	Phytolab, Vestenbergsgreuth, Germany	0.13–1.30	Area = 2 × 10^8^ × conc[mg/mL] − 269,699	0.9997	5.00	8.00
Apigenin	Phytolab, Vestenbergsgreuth, Germany	0.10–0.98	Area = 2 × 10^8^ × conc[mg/mL] + 15,916	0.9999	0.20	0.30
Chrysin	Merck, Darmstadt, Germany	0.10–1.00	Area = 1 × 10^8^ × conc[mg/mL] − 82,818	0.9997	3.00	5.00
Hyperoside	Phytolab, Vestenbergsgreuth, Germany	0.012–0.107	Area = 4 × 10^8^ × conc[mg/mL] − 567,182	0.9986	0.60	0.90
Kaempferol	Phytolab, Vestenbergsgreuth, Germany	0.10–1.00	Area = 10^7^ × conc[mg/mL] − 20,574	0.9996	0.80	1.20
Luteolin	Phytolab, Vestenbergsgreuth, Germany	0.01–0.10	Area = 2 × 10^8^ × conc[mg/mL] − 2295.4	0.9977	0.05	0.07
Luteolin-7-*O*-glucoside	Phytolab, Vestenbergsgreuth, Germany	0.07–0.70	Area = 1 × 10^9^ × conc[mg/mL] − 700,317	0.9990	3.00	4.00
Naringenin	Phytolab, Vestenbergsgreuth, Germany	0.16–1.60	Area = 3 × 10 × conc[mg/mL] − 43,443	0.9999	0.60	0.90
Quercetin	Phytolab, Vestenbergsgreuth, Germany	0.09–0.91	Area = 5 × 10^7^ × conc[mg/mL] − 9556	0.9964	0.80	1.10
Rutoside	Phytolab, Vestenbergsgreuth, Germany	0.17–1.70	Area = 2 × 10^8^ × conc[mg/mL] − 191,937	0.9996	4.00	6.00
Vitexin	Phytolab, Vestenbergsgreuth, Germany	0.17–1.70	Area = 3 × 10^8^ × conc[mg/mL] − 10^6^	0.9996	1.30	2.00

**Table 2:** Polyphenol standards used for the UHPLC–ESI-MS quantitative analysis.

Name of Standard	Retention Time, min	*m*/*z*, and Main Transition	MRM	Other Transitions
Caffeic acid	13.8	179.0 > 135.0	Negative	179.0 > 134.0 179.0 > 89.0
Chlorogenic acid	11.9	353.0 > 191.0	Negative	-
Apigenin	28.1	269.0 > 117.0	Negative	-
Chrysin	29.7	253.0 > 143.0	Negative	253.0 > 119.0 253.0 > 107.0
Hyperoside	20.3	463.1 > 300.0	Negative	463.1 > 301.0
Kaempferol	27.9	285.0 > 187.0	Negative	285.0 > 151.0 285.0 > 133.0
Luteolin	26.8	287.0 > 153.0	Positive	-
Luteolin-*7*-*O*-glucosid	19.9	447.0 > 284.9	Negative	-
Naringenin	26.2	271.0 > 119.0	Negative	271.0 > 107.0
Quercetin	25.4	300.9 > 151.0	Negative	300.9 > 121.0
Rutoside	20.2	609.0 > 300.0	Negative	609.0 > 301.0 609.0 > 271.0
Vitexin	18.4	431.0 > 311.0	Negative	-

**Table 3:** Scheme of the carp larval-seeding experiment.

1.	2.	3.	4.
O-GTE	SA-GTE	Control *Artemia s.*	BM-GTE
5.	6.	7.	8.
Control *Artemia s.*	SA-GTE	BM-GTE	O-GTE
9.	10.	11.	12.
BM-GTE	O-GTE	SA-GTE	Control *Artemia s.*

**Table 4:** The TPC and TFC of the assessed GTEs.

Parameter	O-GTE	SA-GTE	BM-GTE
TPC (mg GAE/mL)	3.934 ± 0.1167	11.308 ± 0.5579	5.805 ± 0.1785
TFC (mg RE/mL)	3.387 ± 0.1048	8.334 ± 0.3941	2.343 ± 0.0984
% of flavonoids from total polyphenols	86.10	73.70	40.36

The results are presented as mean ± SD.

**Table 5:** The quantitative polyphenol profile of GTEs.

Name of Selected Standard and Separated Compound	Standards		O-GTE			SA-GTE			BM-GTE		
	Retention Time (min)	Main MS Transition	Retention Time (min)	Main MS Transition	Content (mg/mL)	Retention Time (min)	Main MS Transition	Content (mg/mL)	Retention Time (min)	Main MS Transition	Content (mg/mL)
Caffeic acid	13.8	179.0 > 135.0				13.5	179.0 > 135.0	0.825 ± 0.0211			
Chlorogenic acid	11.9	353.0 > 191.0	11.9	353.0 > 191.0	0.265 ± 0.0052	11.9	353.0 > 191.0	1.390 ± 0.0417	12.0	353.0 > 191.0	3.539 ± 0.0125
Apigenin	28.1	269.0 > 117.0	28.1	269.0 > 117.0	0.055 ± 0.0041	28.2	269.0 > 117.0	0.017 ± 0.0005	28.2	269.0 > 117.0	0.103 ± 0.0051
Chrysin	29.7	253.0 > 143.0	29.7	253.0 > 143.0	0.109 ± 0.0054	29.7	253.0 > 143.0	0.103 ± 0.0051	29.7	253.0 > 143.0	0.093 ± 0.0004
Hyperoside	20.3	463.1 > 300.0	20.4	463.1 > 300.0	0.202 ± 0.0115	20.2	463.1 > 300.0	1.967 ± 0.0621	20.2	463.1 > 300.0	0.162 ± 0.0415
Kaempferol	27.9	285.0 > 187.0				27.9	285.0 > 187.0	0.032 ± 0.0011			
Luteolin	26.8	287.0 > 153.0	26.7	287.0 > 153.0	0.049 ± 0.0026				26.8	287.0 > 153.0	0.017 ± 0.0009
Luteolin-7-*O*-glucoside	19.9	447.0 > 284.9	19.7	447.0 > 284.9	1.777 ± 0.0217				19.8	447.0 > 284.9	0.072 ± 0.0042
Naringenin	26.2	271.0 > 119.0	26.2	271.0 > 119.0	0.032 ± 0.0017	26.2	271.0 > 119.0	0.011 ± 0.0007	26.3	271.0 > 119.0	0.046 ± 0.0025
Quercetin	25.4	300.9 > 151.0	25.4	300.9 > 151.0	0.052 ± 0.0029	25.4	300.9 > 151.0	0.201 ± 0.0092			
Rutoside	20.2	609.0 > 300.0	20.2	609.0 > 300.0	0.416 ± 0.0231	20.2	609.0 > 300.0	5.506 ± 0.1174	20.2	609.0 > 300.0	1.367 ± 0.0583
Vitexin	18.4	431.0 > 311.0	18.4	431.0 > 311.0	0.034 ± 0.0015						

The results are presented as mean ± SD.

**Table 6:** Evaluation of GTE-specific antioxidant potential.

GTE Sample	DPPH Antioxidant Potential (IC_50_, μL/mL)	FRAP Antioxidant Potential (μM TE/100 mL)	Xanthine Oxidase Inhibition (0.12 IU with 0.015 mL Extract, %)
O	5.35 ± 0.127	1011 ± 7.2	9.91 ± 0.050
SA	10.00 ± 0.249	275 ± 5.5	9.92 ± 0.051
BM	14.16 ± 0.175	205 ± 5.1	9.92 ± 0.051

The results are the mean ± SD.

**Table 7:** Evaluation of GTE specific macronutrient content.

Nutrients (*w*/*w*%)	GTE		
	O	SA	BM
Total protein	1.11	0.860	1.05
Total carbohydrate ^1^	0.719	0.612	0.180
Total carbohydrate ^2^	0.568	0.387	0.096

The results are the mean of three experimental replicates. Total carbohydrate was determined by the ^1^ phenol–sulfuric acid method and ^2^ by the Luff–Schoorl method.

**Table 8:** The nutritional effect of GTEs on carp larvae model.

Feed	Eggs (Day 0)		Non-Feeding Larvae (Day 1)		Feeding Larvae (Day 3)		Feeding Larvae (Day 5)	Feeding Larvae (Day 7)	
	Length (mm)	ATP (ng)	Length (mm)	ATP (pg)	Length (mm)	ATP (pg)	ATP (pg)	Length (mm)	ATP (pg)
O-GTE	1.95 ± 0.05	77.29	5.84 ± 0.10	51.71	7.21 ± 0.09	247.67	86.19	7.77 ± 0.16	74.02
SA-GTE						76.56	168.07		88.98
BM-GTE						99.37	65.403	8.74 ± 0.09	150.58
Artemia						156.92	99.372		240.83

Note: Artemia represents the brine shrimp type of control food.

**Table 9:** Some GTE-specific recorded parameters with values rendered in decreasing order.

GTE/Parameters	TPC	TFC	FRAP	DPPH
O	3	2	1	1
SA	1	1	2	2
BM	2	3	3	3

**Table 10:** Catabolic classification of AAs: (glucogenic), (ketogenic), (glucogenic/ketogenic), (*) limiting AA.

AA		GTE	
		SA	BM
EAA	Arginine—Arg	+	+
	Histidine—His	−	−
	Isoleucine—Ile	+	+
	Leucine—Leu	+	+
	Lysine *—Lys	+	+
	Methionine *—Met	+	−
	Phenylalanine—Phe	+	+
	Threonine *—Thr	+	+
	Tryptophan *—Trp	−	+
	Valine—Val	−	−
NEAA	Alanine—Ala	−	−
	Asparagine—Asn	+	+
	Aspartate (aspartic acid)—Asp	+	+
	Cysteine—Cys	−	−
	Glutamate (glutamic acid)—Glu	+	−
	Glutamine—Gln	−	−
	Glycine—Gly	−	−
	Proline—Pro	+	+
	Serine—Ser	+	−
	Tyrosine—Tyr	−	+
	Citrulline	−	+
	γ-aminobutyric acid —GABA	+	−

Where the meaning are: − absent; + present in the corresponding GTEs.

**Table 11:** The summary of the assessed GTE-associated viability effects. (↑) increased effect, (↑/=) variable but slightly increased effect, (↑↓) highly variable effect.

GTE	Diet Type	Relative Viability		
		3rd Larva Prepupa 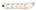	Hatched Adult 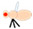	Conc. Dependent Effect
O	0N	lethal	lethal	None
	NM	↑	↑	strong
	HS	↑↓	↑↓	strong
SA	0N	weak	weak	weak
	NM	↑	↑	weak
	HS	↑/=	↑/=	weak
BM	0N	strong	strong	strong
	NM	↑	↑	strong
	HS	↑	↑	strong

## Data Availability

Not applicable.

## Supplementary Materials

The following supporting information can be downloaded at: https://www.mdpi.com/article/10.3390/antiox12091717/s1, Figure S1: Calibration curve for the spectrophotometric determination of total polyphenol content (TPC) of GTEs; Figure S2: Calibration curve for the spectrophotometric determination of total flavonoid content (TFC) of GTEs; Figure S3: Calibration curve for the DPPH assay of the O-GTE (olive); Figure S4: Calibration curve for the DPPH assay of the SA-GTE (sweet almond); Figure S5: Calibration curve for the DPPH assay of the BM-GTE (black mulberry); Figure S6: Chromatogram of caffeic acid obtained in the quantitative analysis of GTE specific selected polyphenols by UHPLC-ESI-MS; Figure S7: Chromatogram of chlorogenic acid obtained in the quantitative analysis of GTE specific selected polyphenols by UHPLC-ESI-MS; Figure S8: Chromatogram of apigenin obtained in the quantitative analysis of GTE specific selected polyphenols by UHPLC-ESI-MS; Figure S9: Chromatogram of chrysin obtained in the quantitative analysis of GTE specific selected polyphenols by UHPLC-ESI-MS; Figure S10: Chromatogram of hyperoside obtained in the quantitative analysis of GTE specific selected polyphenols by UHPLC-ESI-MS; Figure S11: Chromatogram of kaempferol obtained in the quantitative analysis of GTE specific selected polyphenols by UHPLC-ESI-MS; Figure S12: Chromatogram of luteolin obtained in the quantitative analysis of GTE specific selected polyphenols by UHPLC-ESI-MS; Figure S13: Chromatogram of luteolin-7-*O*-glucoside obtained in the quantitative analysis of GTE specific selected polyphenols by UHPLC-ESI-MS; Figure S14: Chromatogram of naringenin obtained in the quantitative analysis of GTE specific selected polyphenols by UHPLC-ESI-MS; Figure S15: Chromatogram of quercetin obtained in the quantitative analysis of GTE specific selected polyphenols by UHPLC-ESI-MS; Figure S16: Chromatogram of rutoside obtained in the quantitative analysis of GTE specific selected polyphenols by UHPLC-ESI-MS; Figure S17: Chromatogram of vitexin obtained in the quantitative analysis of GTE specific selected polyphenols by UHPLC-ESI-MS; Figure S18: MS spectrum of caffeic acid obtained in the quantitative analysis of GTE specific selected polyphenols by UHPLC-ESI-MS; Figure S19: MS spectrum of chlorogenic acid obtained in the quantitative analysis of GTE specific selected polyphenols by UHPLC-ESI-MS; Figure S20: MS spectrum of apigenin obtained in the quantitative analysis of GTE specific selected polyphenols by UHPLC-ESI-MS; Figure S21: MS spectrum of chrysin obtained in the quantitative analysis of GTE specific selected polyphenols by UHPLC-ESI-MS; Figure S22: MS spectrum of hyperoside obtained in the quantitative analysis of GTE specific selected polyphenols by UHPLC-ESI-MS; Figure S23: MS spectrum of kaempferol obtained in the quantitative analysis of GTE specific selected polyphenols by UHPLC-ESI-MS; Figure S24: MS spectrum of luteolin obtained in the quantitative analysis of GTE specific selected polyphenols by UHPLC-ESI-MS; Figure S25: MS spectrum of luteolin-7-*O*-glucoside obtained in the quantitative analysis of GTE specific selected polyphenols by UHPLC-ESI-MS; Figure S26: MS spectrum of naringenin obtained in the quantitative analysis of GTE specific selected polyphenols by UHPLC-ESI-MS; Figure S27: MS spectrum of quercetin obtained in the quantitative analysis of GTE specific selected polyphenols by UHPLC-ESI-MS; Figure S28: MS spectrum of rutoside obtained in the quantitative analysis of GTE specific selected polyphenols by UHPLC-ESI-MS; Figure S29: MS spectrum of vitexin obtained in the quantitative analysis of GTE specific selected polyphenols by UHPLC-ESI-MS; Figure S30: Chromatogram of chlorogenic acid obtained in the quantitative analysis of selected polyphenols of the O-GTE (olive); Figure S31: Chromatogram of apigenin obtained in the quantitative analysis of selected polyphenols of the O-GTE (olive); Figure S32: Chromatogram of chrysin obtained in the quantitative analysis of selected polyphenols of the O-GTE (olive); Figure S33: Chromatogram of hyperoside obtained in the quantitative analysis of selected polyphenols of the O-GTE (olive); Figure S34: Chromatogram of luteolin obtained in the quantitative analysis of selected polyphenols of the O-GTE (olive); Figure S35: Chromatogram of luteolin-7-*O*-glucoside obtained in the quantitative analysis of selected polyphenols of the O-GTE (olive); Figure S36: Chromatogram of narigenin obtained in the quantitative analysis of selected polyphenols of the O-GTE (olive); Figure S37: Chromatogram of quercetin obtained in the quantitative analysis of selected polyphenols of the O-GTE (olive); Figure S38: Chromatogram of rutoside obtained in the quantitative analysis of selected polyphenols of the O-GTE (olive); Figure S39: Chromatogram of vitexin obtained in the quantitative analysis of selected polyphenols of the O-GTE (olive); Figure S40: MS spectrum of chlorogenic acid obtained in the quantitative analysis of selected polyphenols of the O-GTE (olive); Figure S41: MS spectrum of apigenin obtained in the quantitative analysis of selected polyphenols of the O-GTE (olive); Figure S42: MS spectrum of chrysin obtained in the quantitative analysis of selected polyphenols of the O-GTE (olive); Figure S43: MS spectrum of hyperoside obtained in the quantitative analysis of selected polyphenols of the O-GTE (olive); Figure S44: MS spectrum of luteolin obtained in the quantitative analysis of selected polyphenols of the O-GTE (olive); Figure S45: MS spectrum of luteolin-7-*O*-glucoside obtained in the quantitative analysis of selected polyphenols of the O-GTE (olive); Figure S46: MS spectrum of naringenin obtained in the quantitative analysis of selected polyphenols of the O-GTE (olive); Figure S47: MS spectrum of quercetin obtained in the quantitative analysis of selected polyphenols of the O-GTE (olive); Figure S48: MS spectrum of rutoside obtained in the quantitative analysis of selected polyphenols of the O-GTE (olive); Figure S49: MS spectrum of vitexin obtained in the quantitative analysis of selected polyphenols of the O-GTE (olive); Figure S50: Chromatogram of chlorogenic acid obtained in the quantitative analysis of selected polyphenols of the SA-GTE (sweet almond); Figure S51: Chromatogram of caffeic acid obtained in the quantitative analysis of selected polyphenols of the SA-GTE (sweet almond); Figure S52: Chromatogram of chrysin obtained in the quantitative analysis of selected polyphenols of the SA-GTE (sweet almond); Figure S53: Chromatogram of hyperoside obtained in the quantitative analysis of selected polyphenols of the SA-GTE (sweet almond); Figure S54: Chromatogram of naringenin obtained in the quantitative analysis of selected polyphenols of the SA-GTE (sweet almond); Figure S55: Chromatogram of quercetin obtained in the quantitative analysis of selected polyphenols of the SA-GTE (sweet almond); Figure S56: Chromatogram of rutoside obtained in the quantitative analysis of selected polyphenols of the SA-GTE (sweet almond); Figure S57: Chromatogram of kaempferol obtained in the quantitative analysis of selected polyphenols of the SA-GTE (sweet almond); Figure S58: MS spectrum of chlorogenic acid obtained in the quantitative analysis of selected polyphenols of the SA-GTE (sweet almond); Figure S59: MS spectrum of caffeic acid obtained in the quantitative analysis of selected polyphenols of the SA-GTE (sweet almond); Figure S60: MS spectrum of chrysin obtained in the quantitative analysis of selected polyphenols of the SA-GTE (sweet almond); Figure S61: MS spectrum of hyperoside obtained in the quantitative analysis of selected polyphenols of the SA-GTE (sweet almond); Figure S62: MS spectrum of naringenin obtained in the quantitative analysis of selected polyphenols of the SA-GTE (sweet almond); Figure S63: MS spectrum of quercetin obtained in the quantitative analysis of selected polyphenols of the SA-GTE (sweet almond); Figure S64: MS spectrum of rutoside obtained in the quantitative analysis of selected polyphenols of the SA-GTE (sweet almond); Figure S65: MS spectrum of kaempferol obtained in the quantitative analysis of selected polyphenols of the SA-GTE (sweet almond); Figure S66: Chromatogram of chlorogenic acid obtained in the quantitative analysis of selected polyphenols of the BM-GTE (black mulberry); Figure S67: Chromatogram of apigenin obtained in the quantitative analysis of selected polyphenols of the BM-GTE (black mulberry); Figure S68: Chromatogram of chrysin obtained in the quantitative analysis of selected polyphenols of the BM-GTE (black mulberry); Figure S69: Chromatogram of hyperoside obtained in the quantitative analysis of selected polyphenols of the BM-GTE (black mulberry); Figure S70: Chromatogram of neringenin obtained in the quantitative analysis of selected polyphenols of the BM-GTE (black mulberry); Figure S71: Chromatogram of rutoside obtained in the quantitative analysis of selected polyphenols of the BM-GTE (black mulberry); Figure S72: Chromatogram of luteolin obtained in the quantitative analysis of selected polyphenols of the BM-GTE (black mulberry); Figure S73: Chromatogram of luteolin-7-*O*-glucoside obtained in the quantitative analysis of selected polyphenols of the BM-GTE (black mulberry); Figure S74: MS spectrum of chlorogenic acid obtained in the quantitative analysis of selected polyphenols of the BM-GTE (black mulberry); Figure S75: MS spectrum of apigenin obtained in the quantitative analysis of selected polyphenols of the BM-GTE (black mulberry); Figure S76: MS spectrum of chrysin obtained in the quantitative analysis of selected polyphenols of the BM-GTE (black mulberry); Figure S77: MS spectrum of hyperoside obtained in the quantitative analysis of selected polyphenols of the BM-GTE (black mulberry); Figure S78: MS spectrum of naringenin obtained in the quantitative analysis of selected polyphenols of the BM-GTE (black mulberry); Figure S79: MS spectrum of rutoside obtained in the quantitative analysis of selected polyphenols of the BM-GTE (black mulberry); Figure S80: MS spectrum of luteolin obtained in the quantitative analysis of selected polyphenols of the BM-GTE (black mulberry); Figure S81: MS spectrum of luteolin-7-*O*-glucoside obtained in the quantitative analysis of selected polyphenols of the BM-GTE (black mulberry); Table S1: Protocol of the quantitative analysis of GTE specific selected polyphenols by UHPLC-ESI-MS; Table S2: The phytonutrient profile of O-GTE (olive); Table S3: The phytonutrient profile of SA-GTE (sweet almond); Table S4: The phytonutrient profile of BM-GTE (black mulberry).
