# Supplementary material for: Phytoconstituent Profiles Associated with Relevant Antioxidant Potential and Variable Nutritive Effects of the Olive, Sweet Almond, and Black Mulberry Gemmotherapy Extracts
Source: Antioxidants (Basel). 2023 Sep 4;12(9):1717. doi: 10.3390/antiox12091717 (PMC10525884; doi:10.3390/antiox12091717)
Supplement: Supplementary file 1 [file antioxidants-12-01717-s001.zip › antioxidants-2534459-supplementary.pdf]

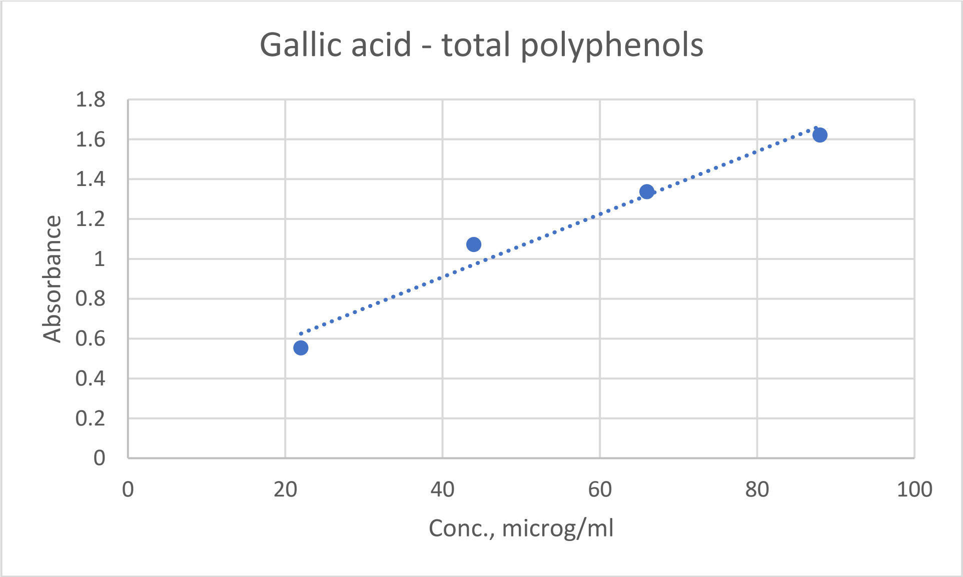

Figure S1. Calibration curve for the spectrophotometric determination of Total Polyphenol Content (TPC) of GTEs.

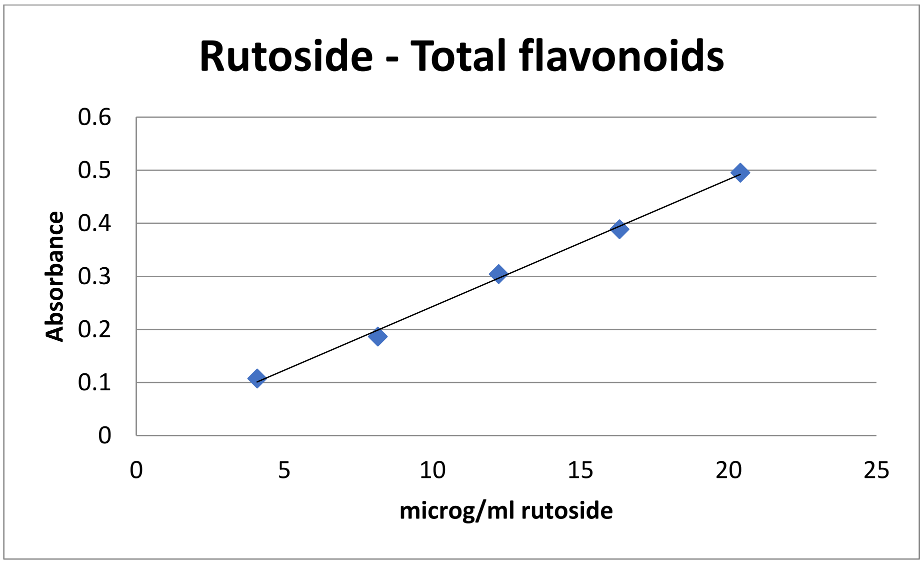

Figure S2. Calibration curve for the spectrophotometric determination of Total Flavonoid Content (TFC) of GTEs

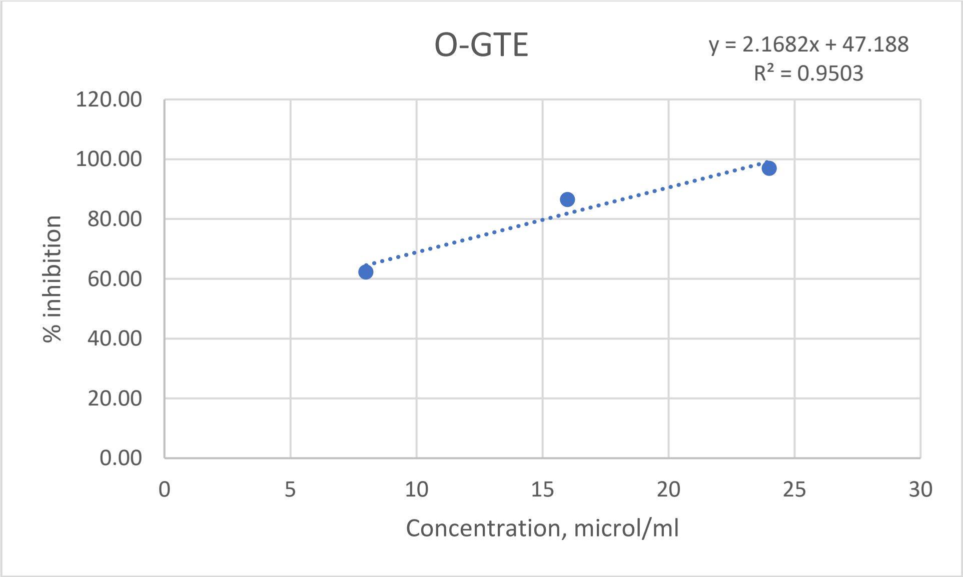

Figure S3. Calibration curve for the DPPH assay of the O-GTE (olive)

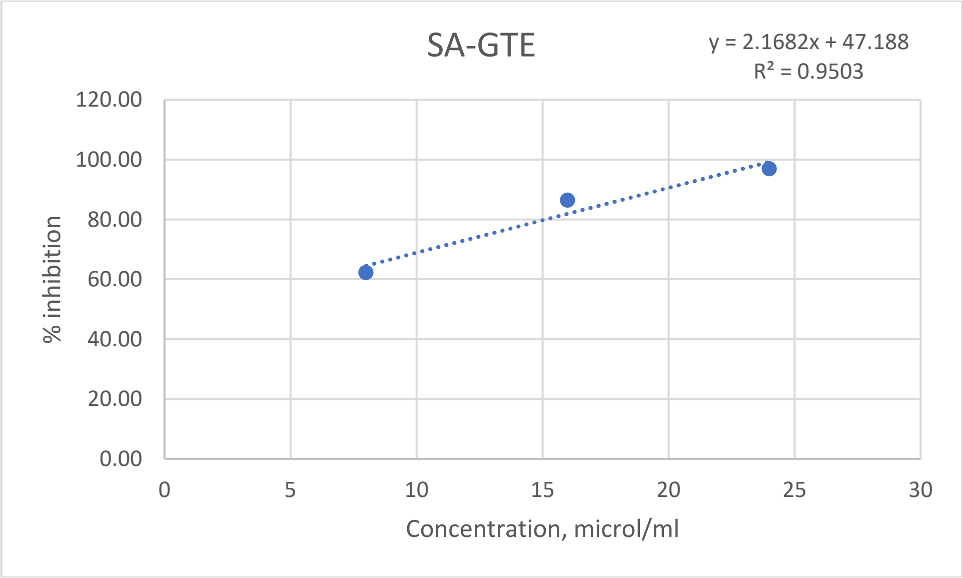

Figure S4. Calibration curve for the DPPH assay of the SA-GTE (sweet almond)

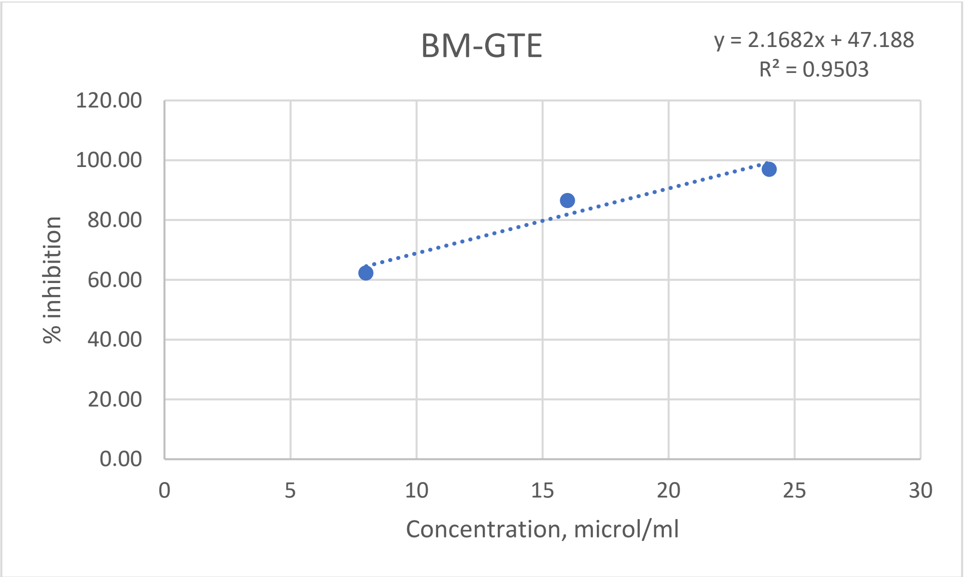

Figure S5. Calibration curve for the DPPH assay of the BM-GTE (black mulberry)

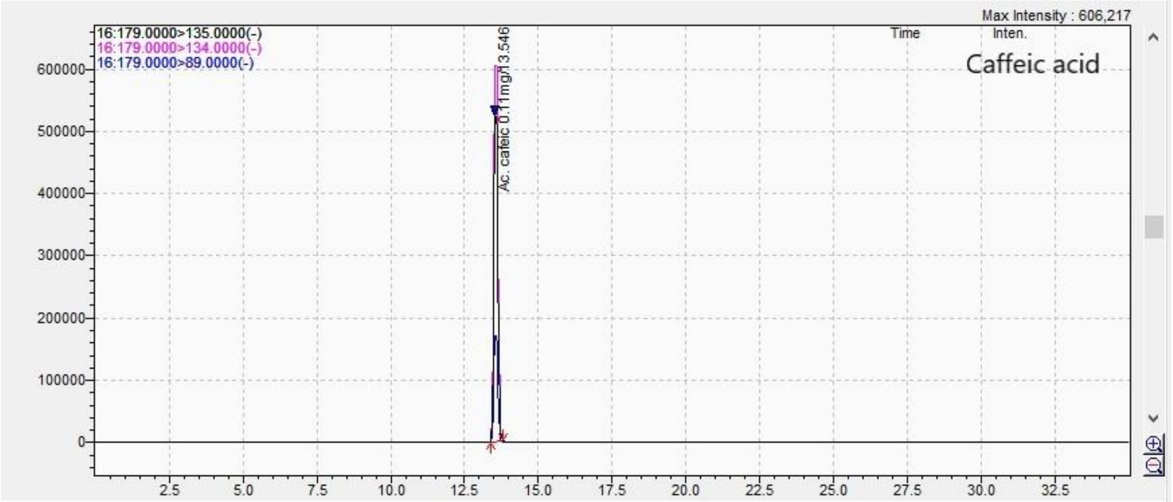

Figure S6. Chromatogram of caffeic acid obtained in the quantitative analysis of GTE specific selected polyphenols by UHPLC-ESI-MS.

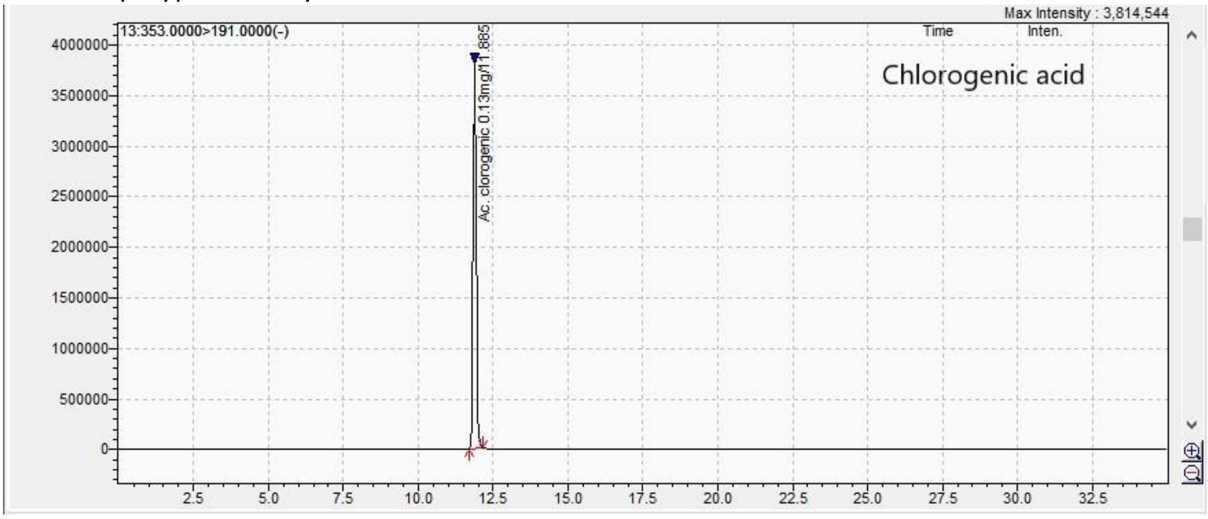

Figure S7. Chromatogram of chlorogenic acid obtained in the quantitative analysis of GTE specific selected polyphenols by UHPLC-ESI-MS.

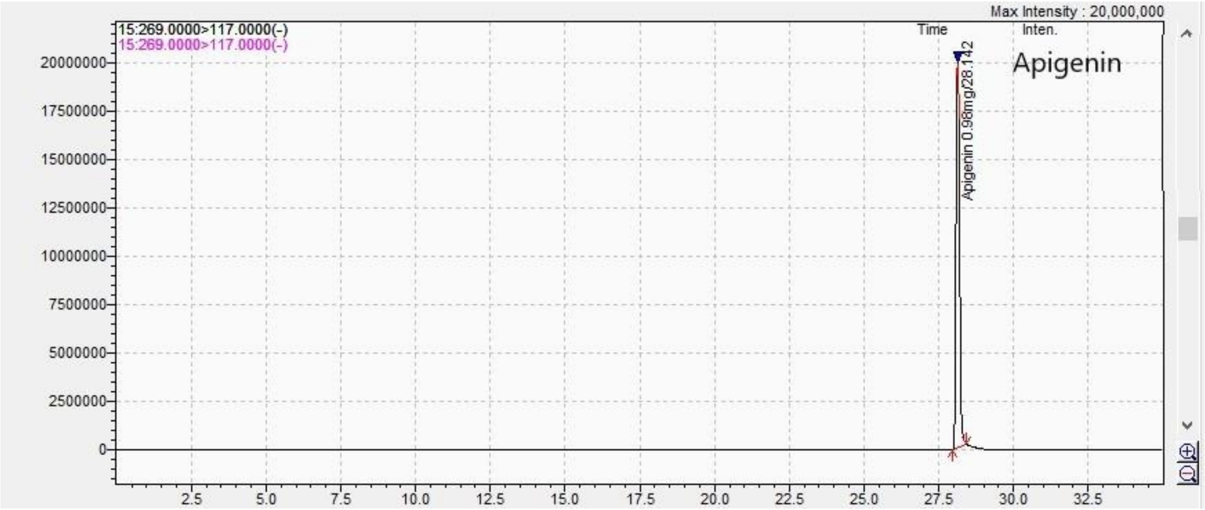

Figure S8. Chromatogram of apigenin obtained in the quantitative analysis of GTE specific selected polyphenols by UHPLC-ESI-MS.

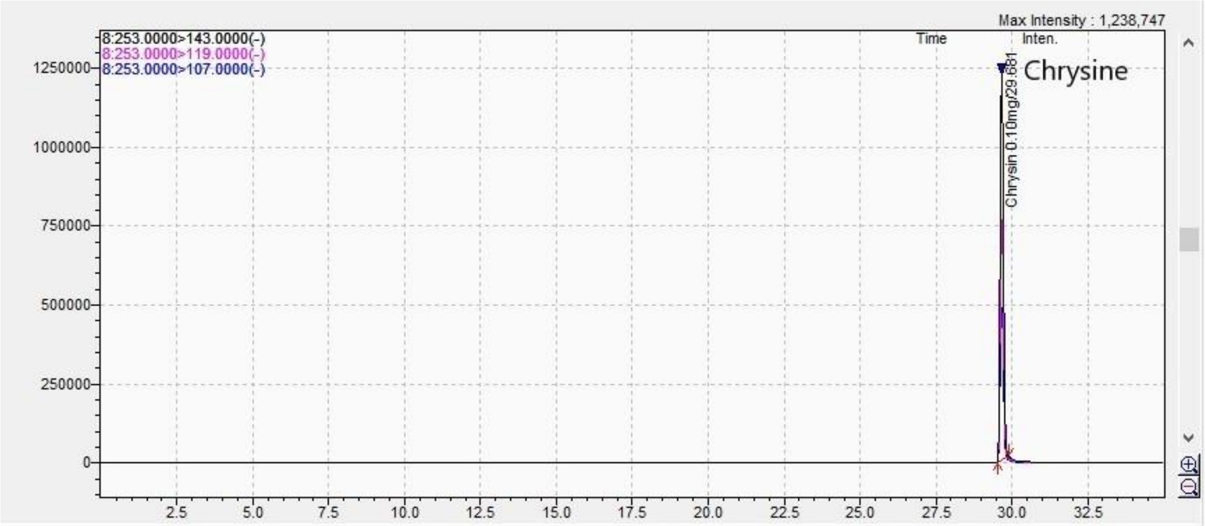

Figure S9. Chromatogram of chrysin obtained in the quantitative analysis of GTE specific selected polyphenols by UHPLC-ESI-MS.

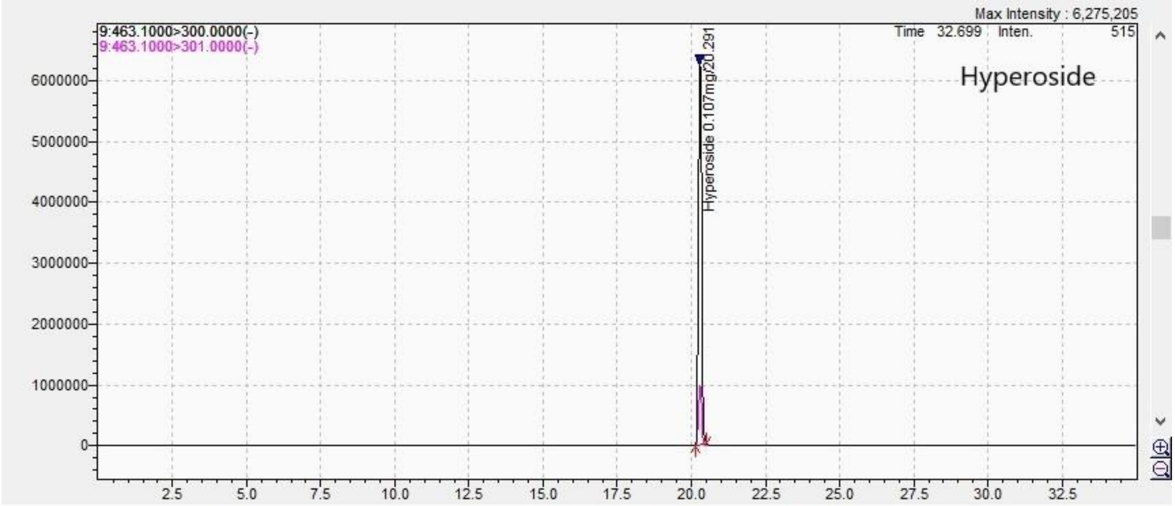

Figure S10. Chromatogram of hyperoside obtained in the quantitative analysis of GTE specific selected polyphenols by UHPLC-ESI-MS.

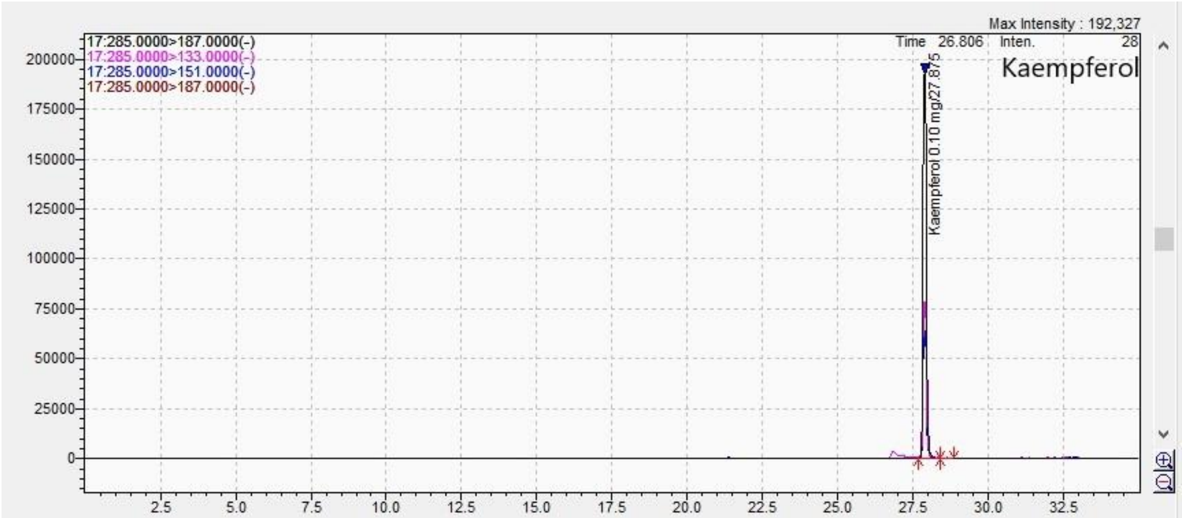

Figure S11. Chromatogram of kaempferol obtained in the quantitative analysis of GTE specific selected polyphenols by UHPLC-ESI-MS.

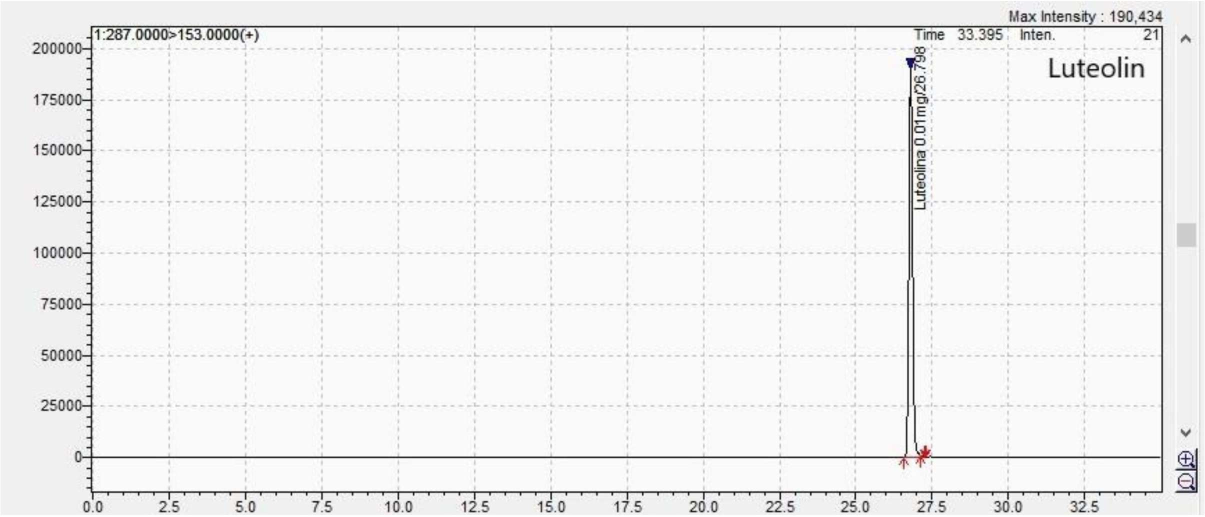

Figure S12. Chromatogram of luteolin obtained in the quantitative analysis of GTE specific selected polyphenols by UHPLC-ESI-MS.

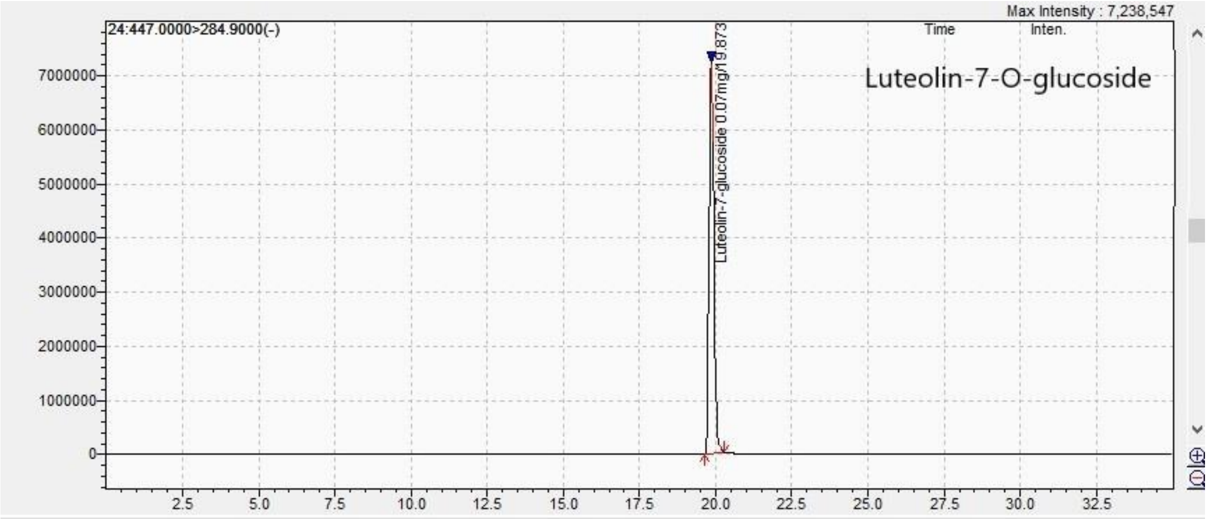

Figure S13. Chromatogram of luteolin-*7-o*-glucoside obtained in the quantitative analysis of GTE specific selected polyphenols by UHPLC-ESI-MS.

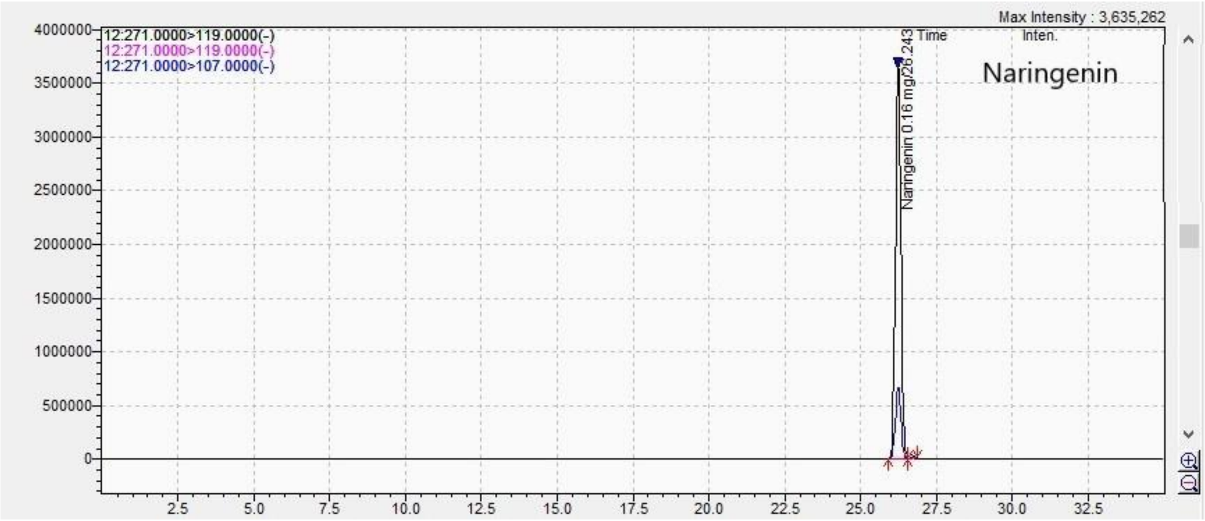

Figure S14. Chromatogram of naringenin obtained in the quantitative analysis of GTE specific selected polyphenols by UHPLC-ESI-MS.

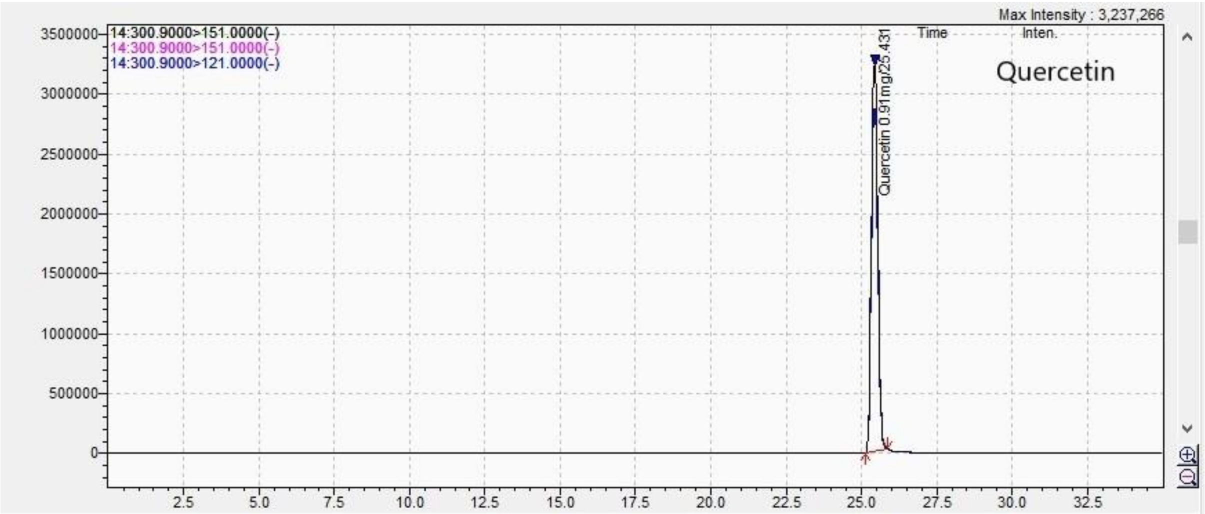

Figure S15. Chromatogram of quercetin obtained in the quantitative analysis of GTE specific selected polyphenols by UHPLC-ESI-MS.

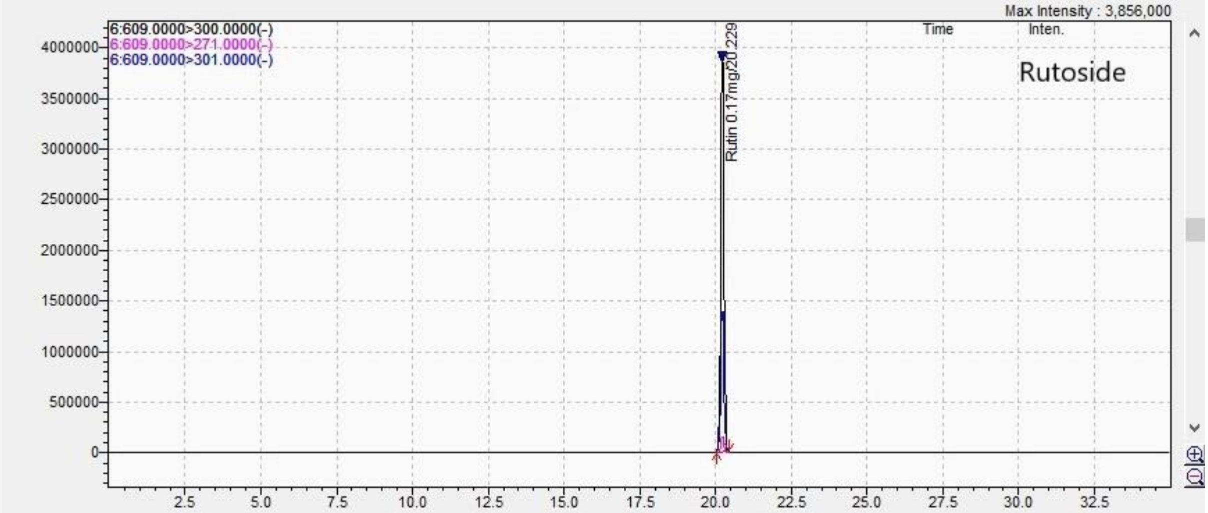

Figure S16. Chromatogram of rutoside obtained in the quantitative analysis of GTE specific selected polyphenols by UHPLC-ESI-MS.

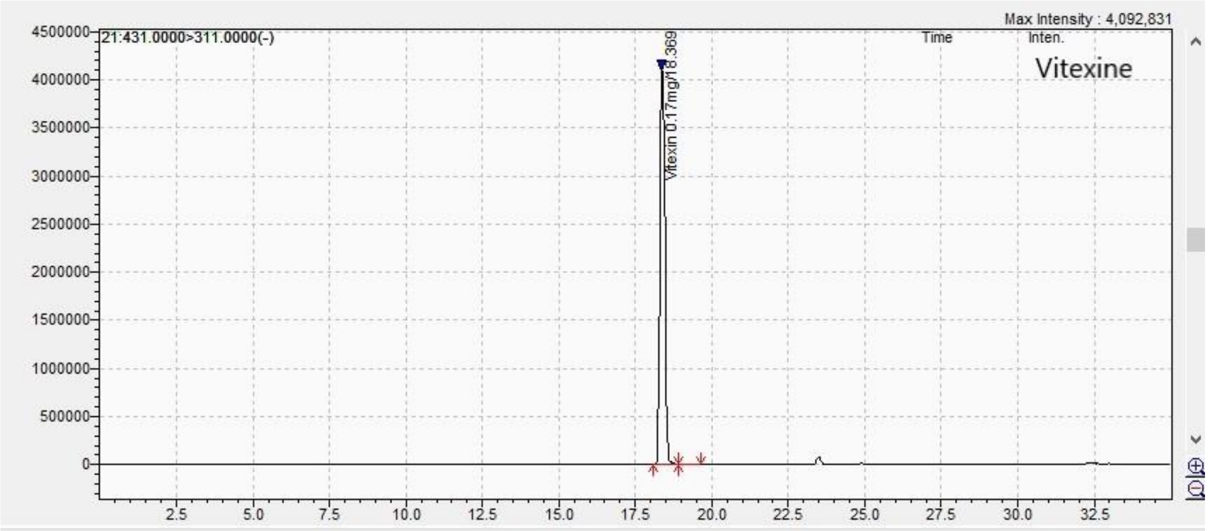

Figure S17. Chromatogram of vitexin obtained in the quantitative analysis of GTE specific selected polyphenols by UHPLC-ESI-MS.

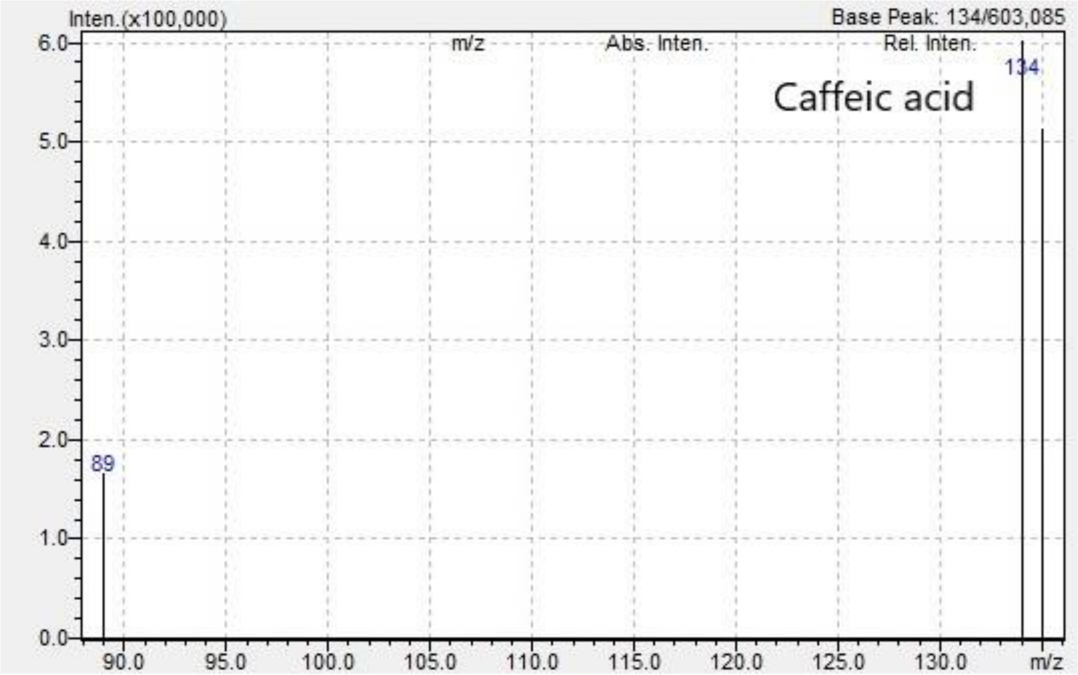

Figure S18. MS spectrum of caffeic acid obtained in the quantitative analysis of GTE specific selected polyphenols by UHPLC-ESI-MS

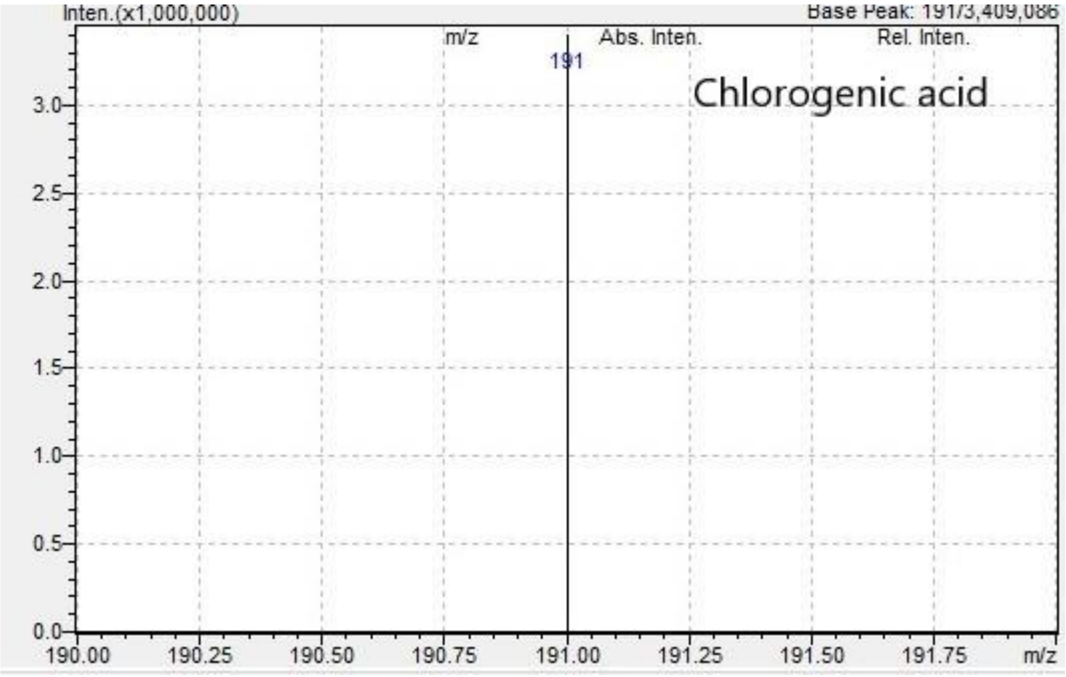

Figure S19. MS spectrum of chlorogenic acid obtained in the quantitative analysis of GTE specific selected polyphenols by UHPLC-ESI-MS

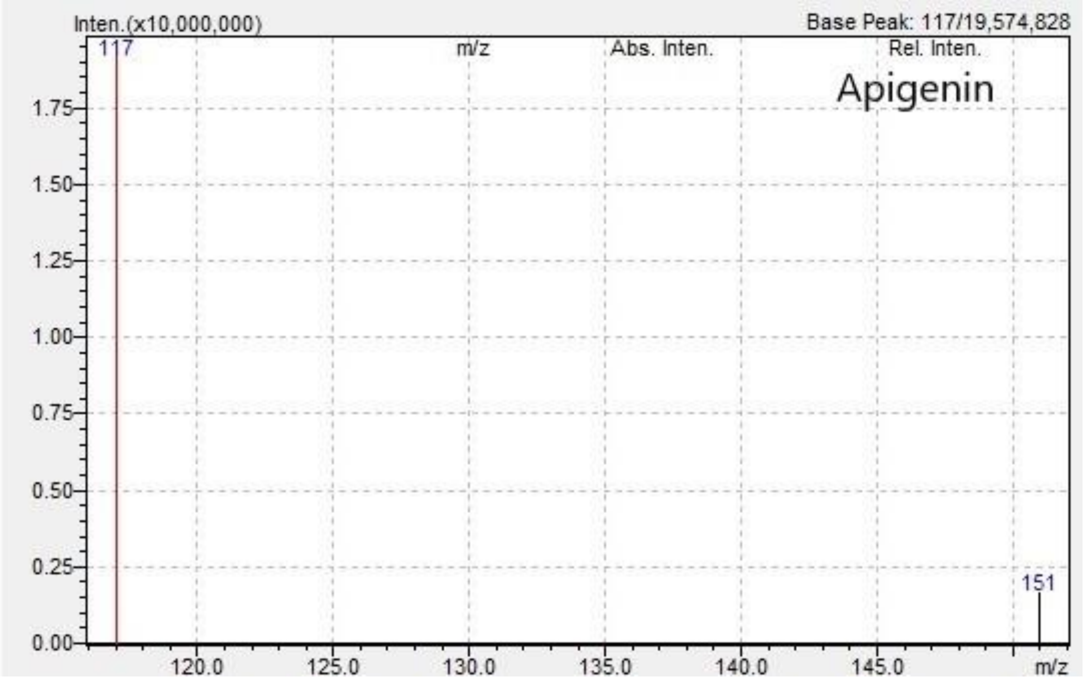

Figure S20. MS spectrum of apigenin obtained in the quantitative analysis of GTE specific selected polyphenols by UHPLC-ESI-MS

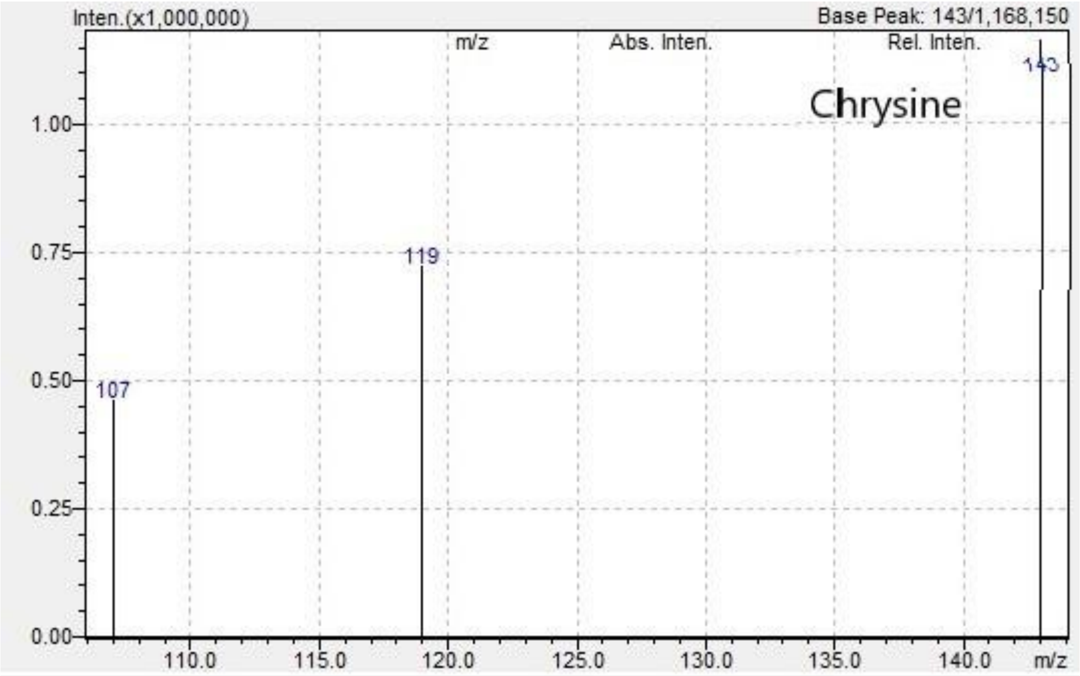

Figure S21. MS spectrum of chrysin obtained in the quantitative analysis of GTE specific selected polyphenols by UHPLC-ESI-MS

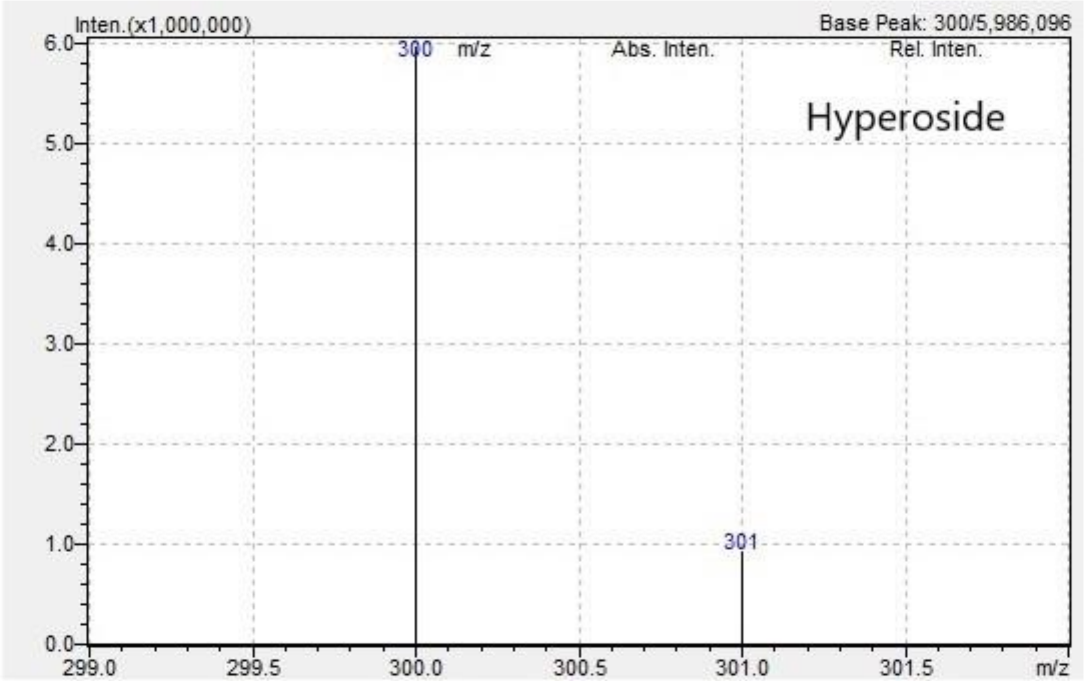

Figure S22. MS spectrum of hyperoside obtained in the quantitative analysis of GTE specific selected polyphenols by UHPLC-ESI-MS

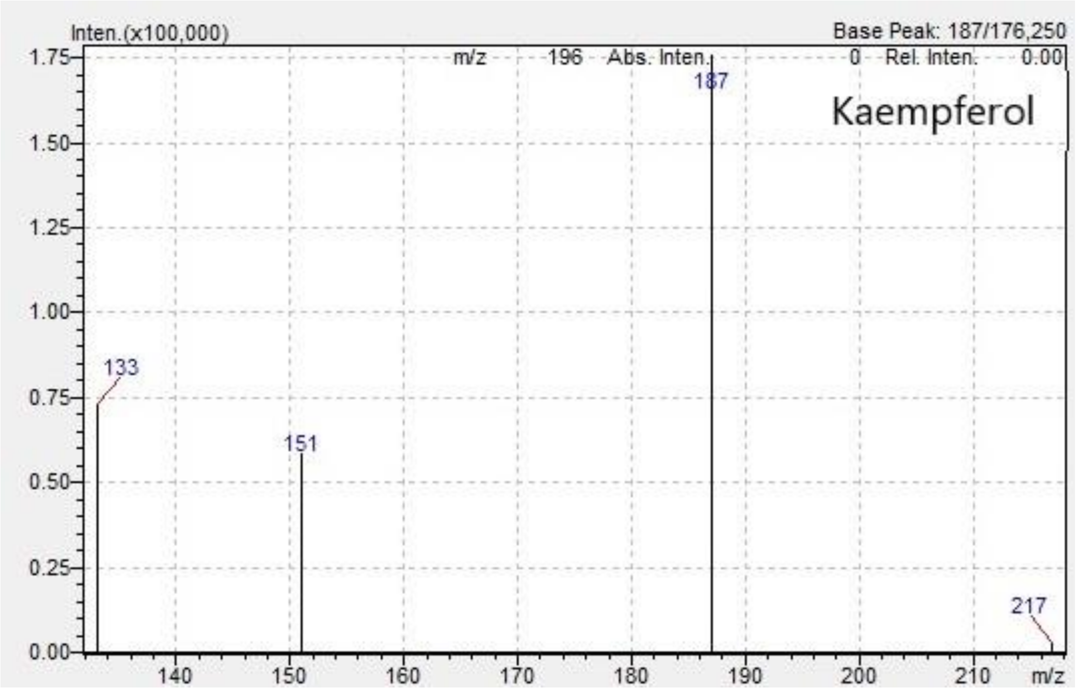

Figure S23. MS spectrum of kaempferol obtained in the quantitative analysis of GTE specific selected polyphenols by UHPLC-ESI-MS

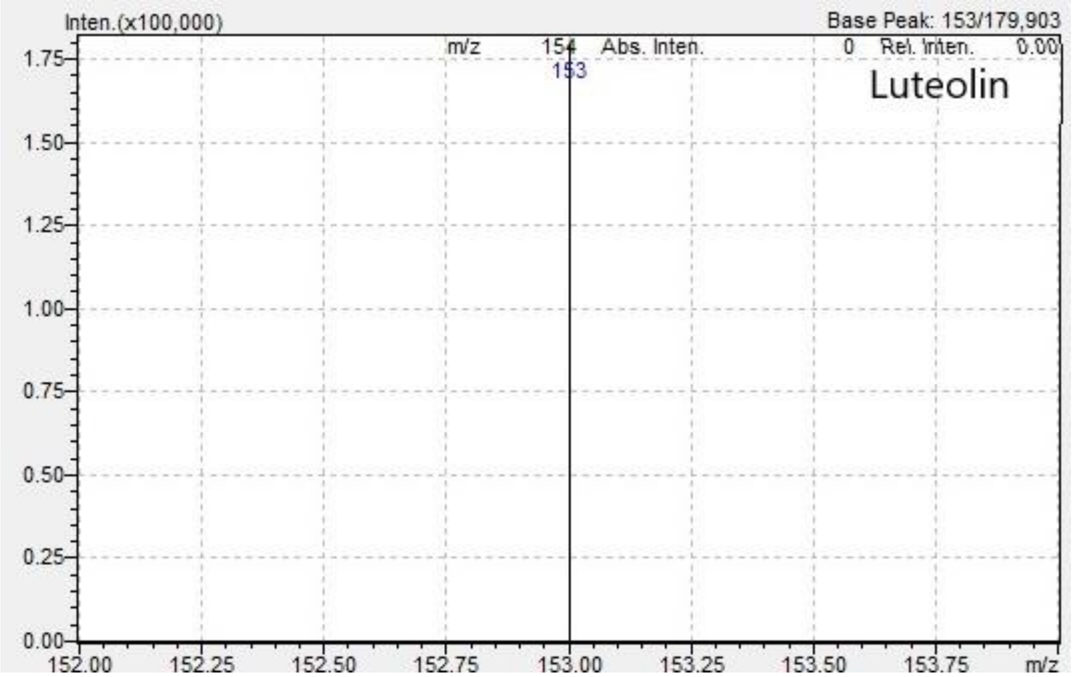

Figure S24. MS spectrum of luteolin obtained in the quantitative analysis of GTE specific selected polyphenols by UHPLC-ESI-MS

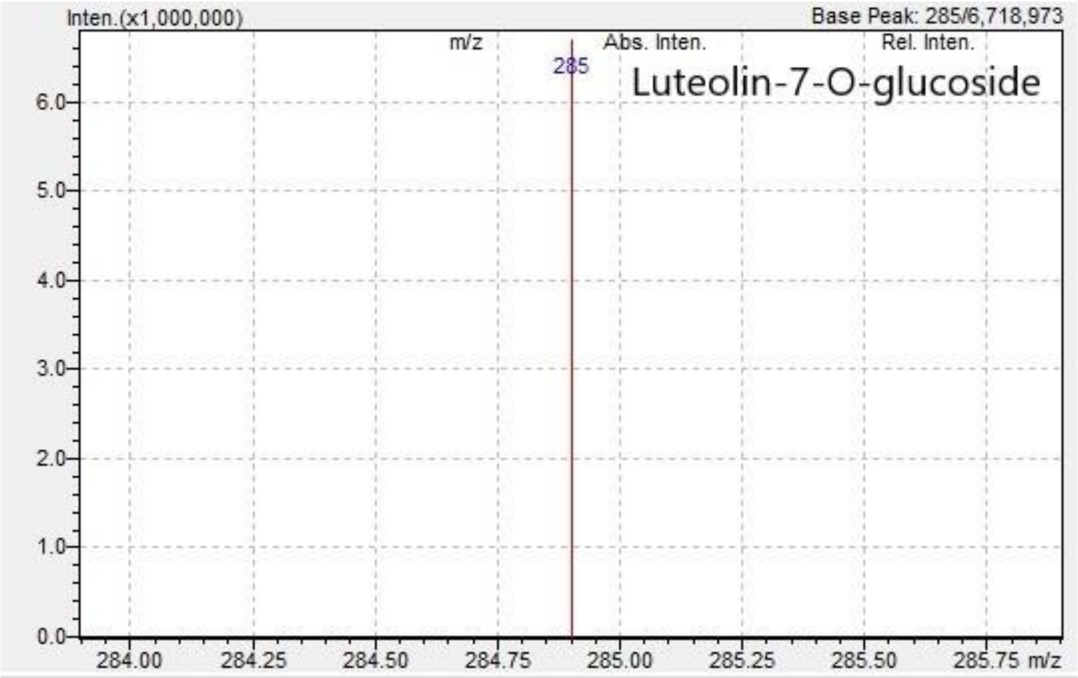

Figure S25. MS spectrum of luteolin-*7-O*-glucoside obtained in the quantitative analysis of GTE specific selected polyphenols by UHPLC-ESI-MS

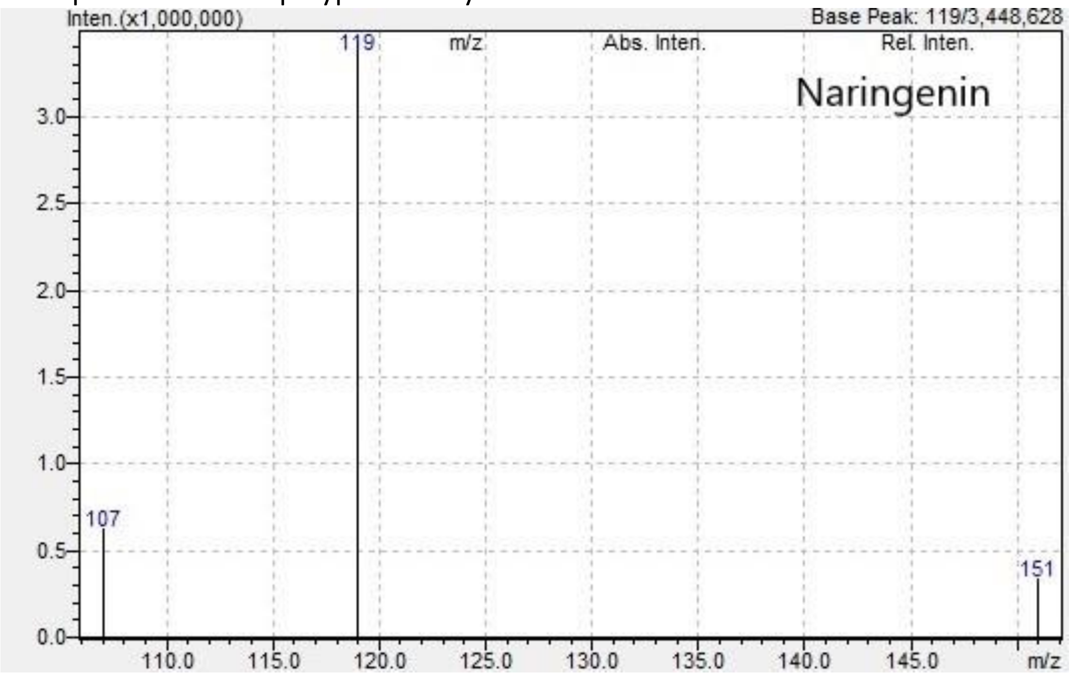

Figure S26. MS spectrum of naringenin obtained in the quantitative analysis of GTE specific selected polyphenols by UHPLC-ESI-MS

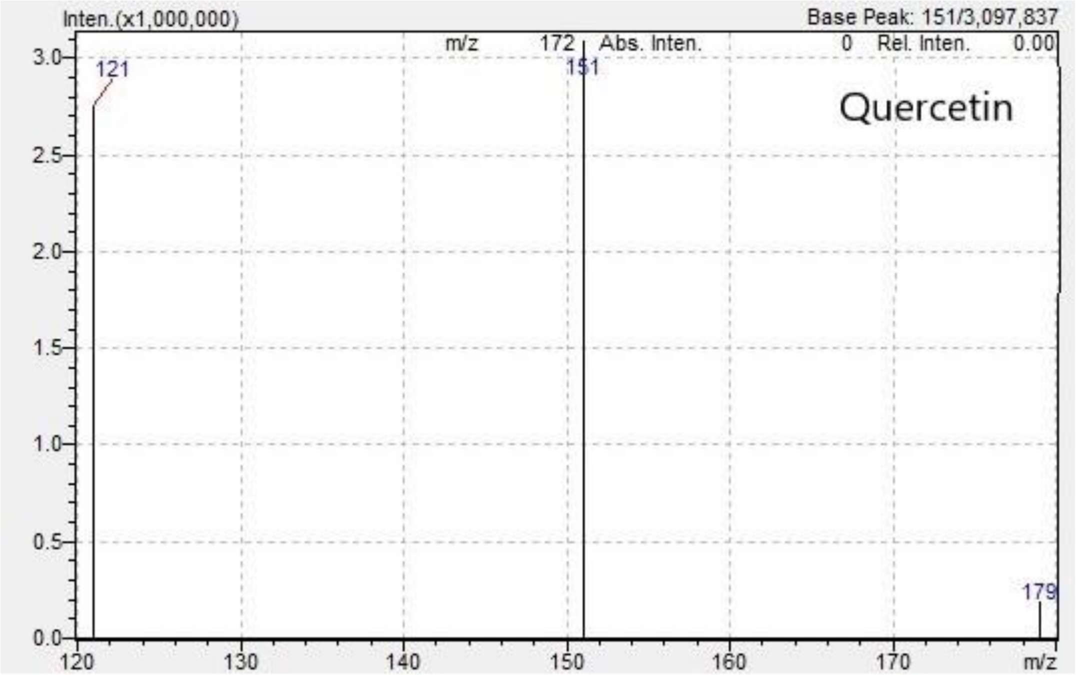

Figure S27. MS spectrum of quercetin obtained in the quantitative analysis of GTE specific selected polyphenols by UHPLC-ESI-MS

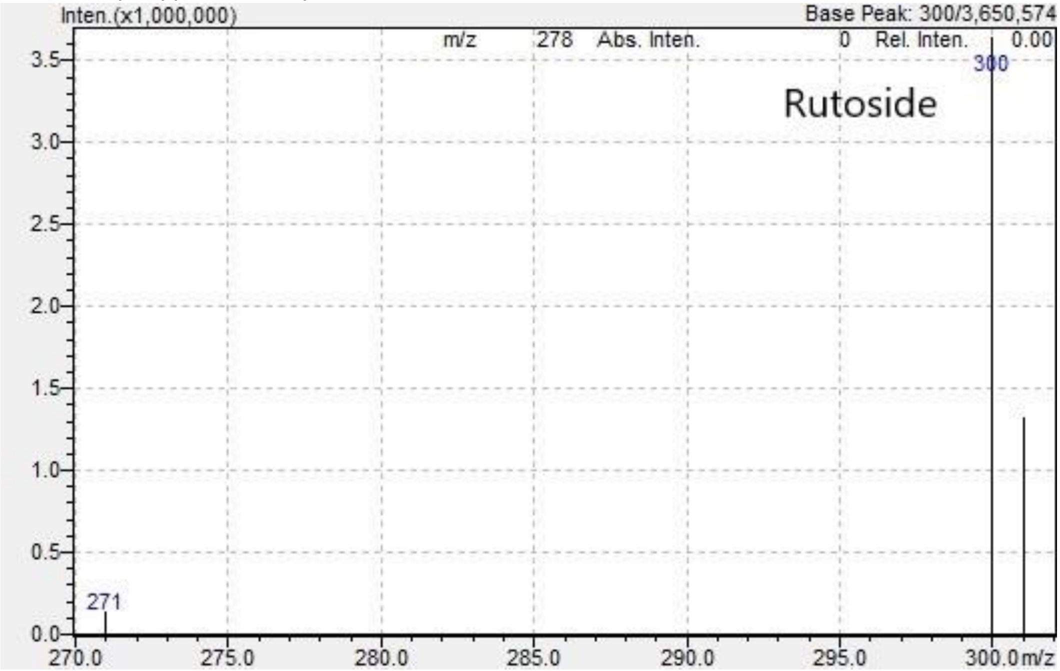

Figure S28. MS spectrum of rutoside obtained in the quantitative analysis of GTE specific selected polyphenols by UHPLC-ESI-MS

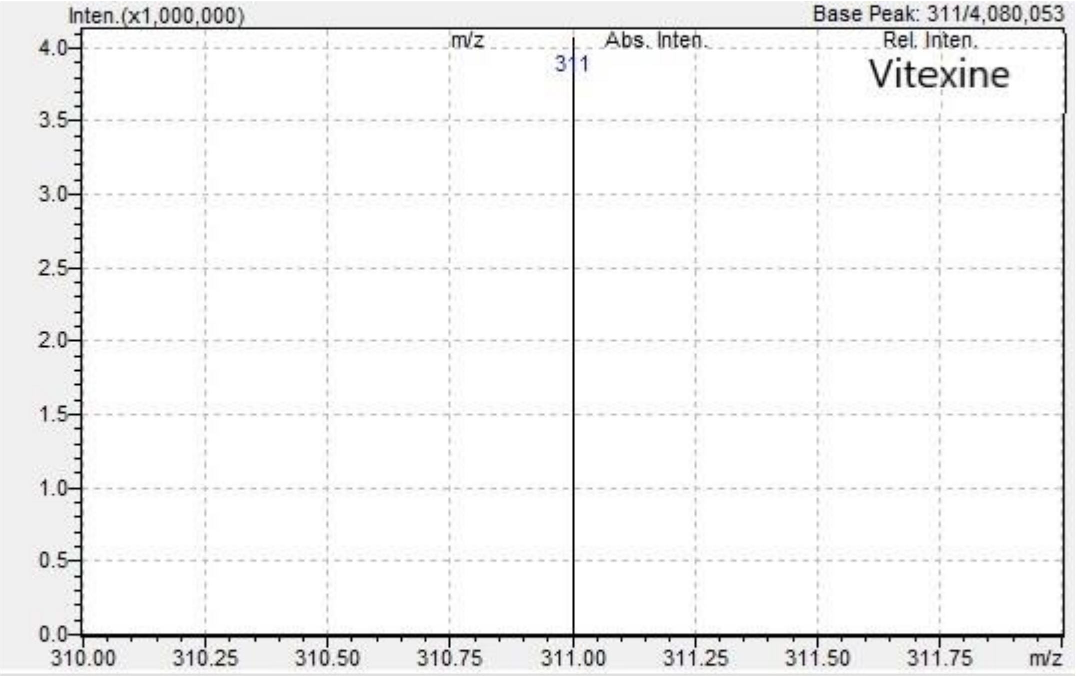

Figure S29. MS spectrum of vitexin obtained in the quantitative analysis of GTE specific selected polyphenols by UHPLC-ESI-MS

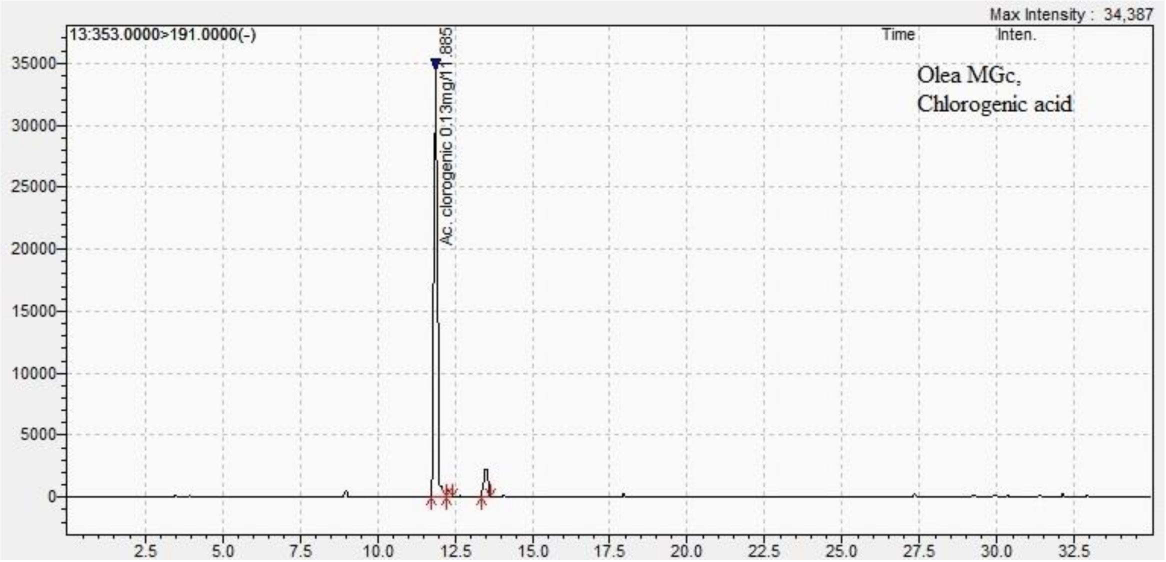

Figure S30. Chromatogram of chlorogenic acid obtained in the quantitative analysis of selected polyphenols of the O-GTE (olive)

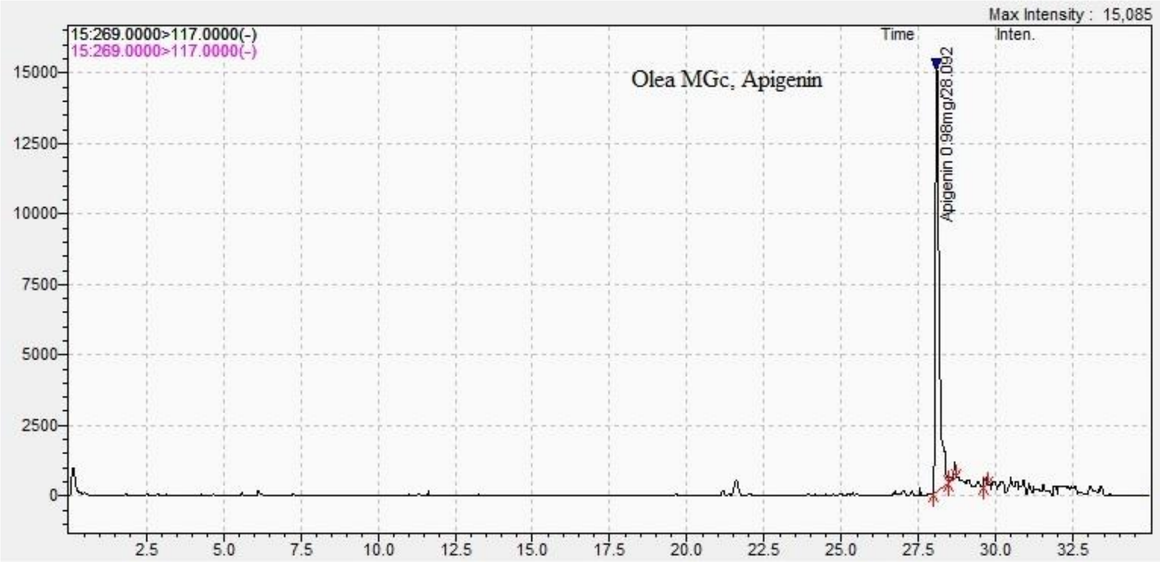

Figure S31. Chromatogram of apigenin obtained in the quantitative analysis of selected polyphenols of the O-GTE (olive)

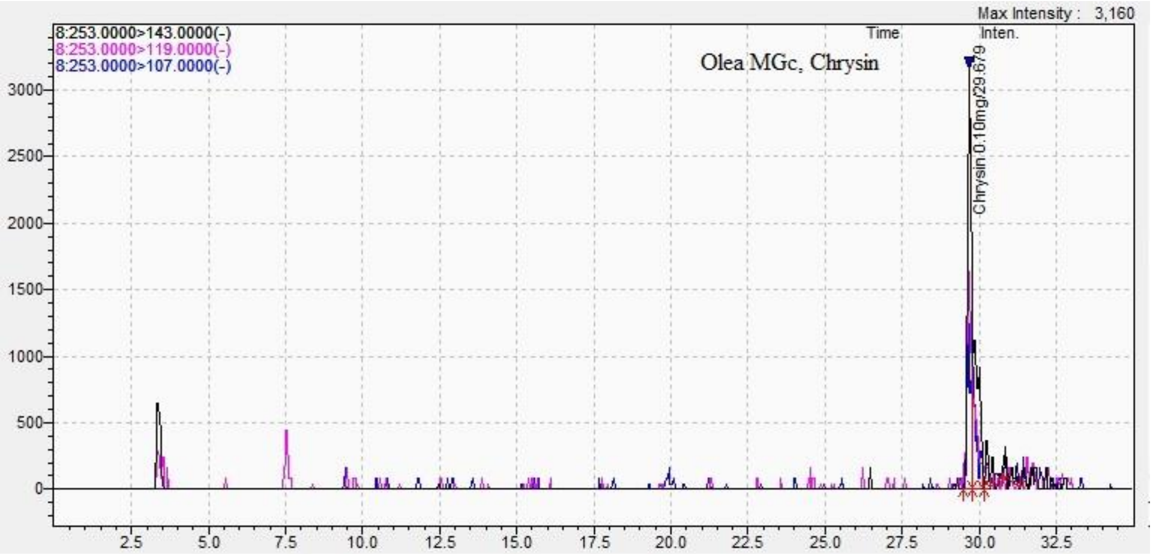

Figure S32. Chromatogram of chrysin obtained in the quantitative analysis of selected polyphenols of the O-GTE (olive)

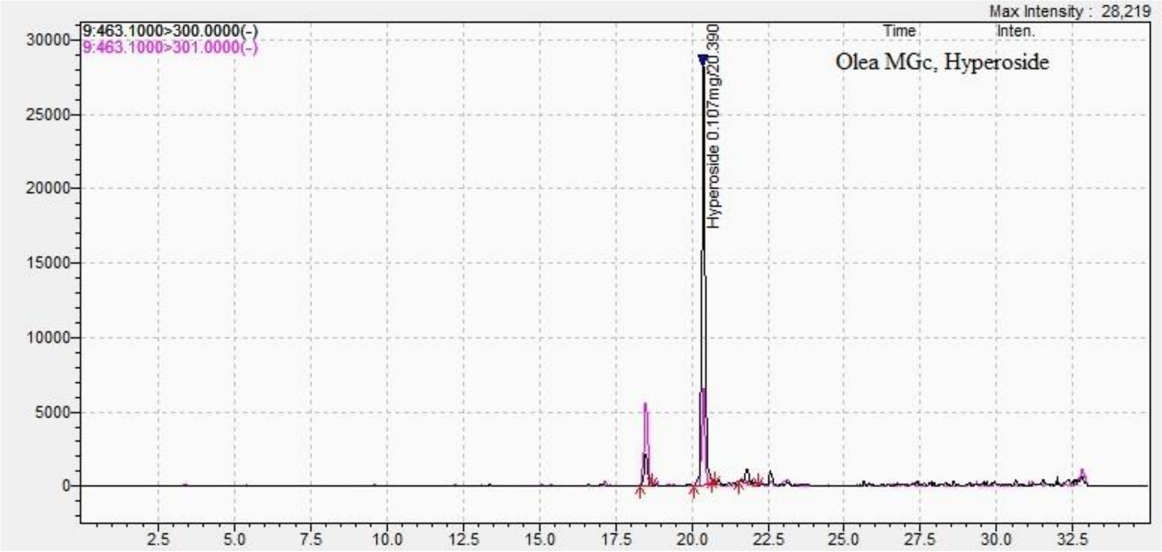

Figure S33. Chromatogram of hyperoside obtained in the quantitative analysis of selected polyphenols of the O-GTE (olive)

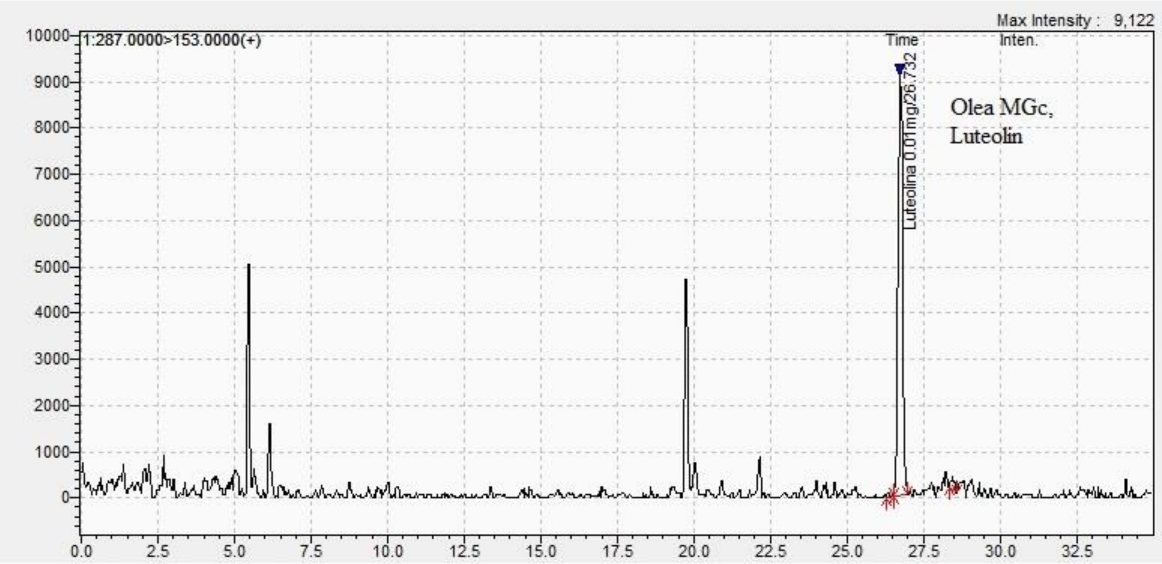

Figure S34. Chromatogram of luteolin obtained in the quantitative analysis of selected polyphenols of the O-GTE (olive)

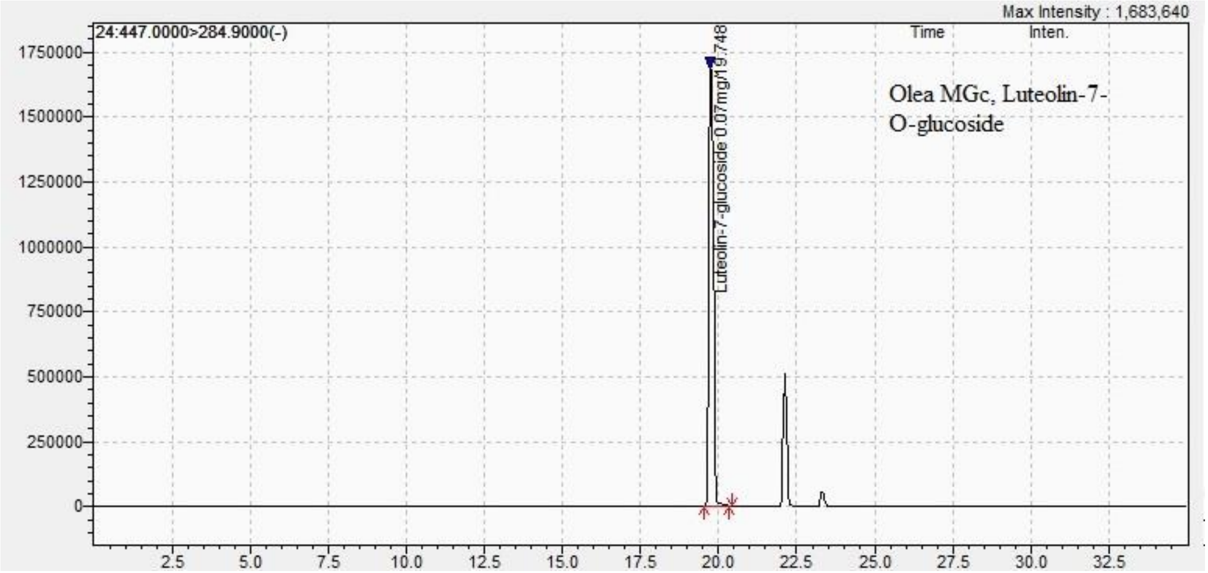

Figure S35. Chromatogram of luteolin-*7-O*-glucoside obtained in the quantitative analysis of selected polyphenols of the O-GTE (olive)

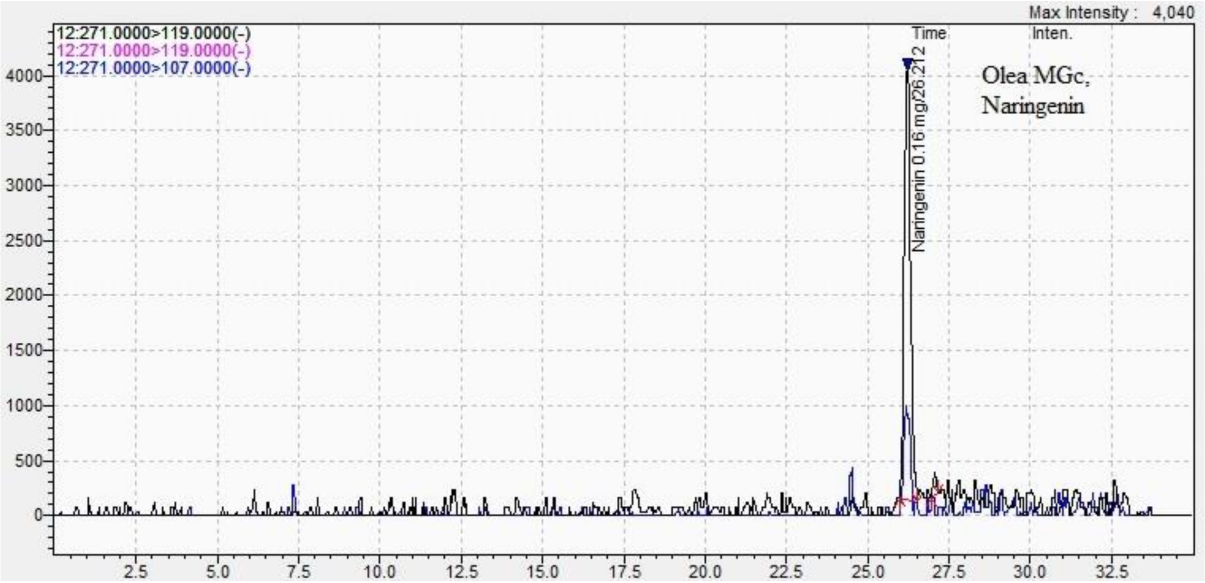

Figure S36. Chromatogram of naringenin obtained in the quantitative analysis of selected polyphenols of the O-GTE (olive)

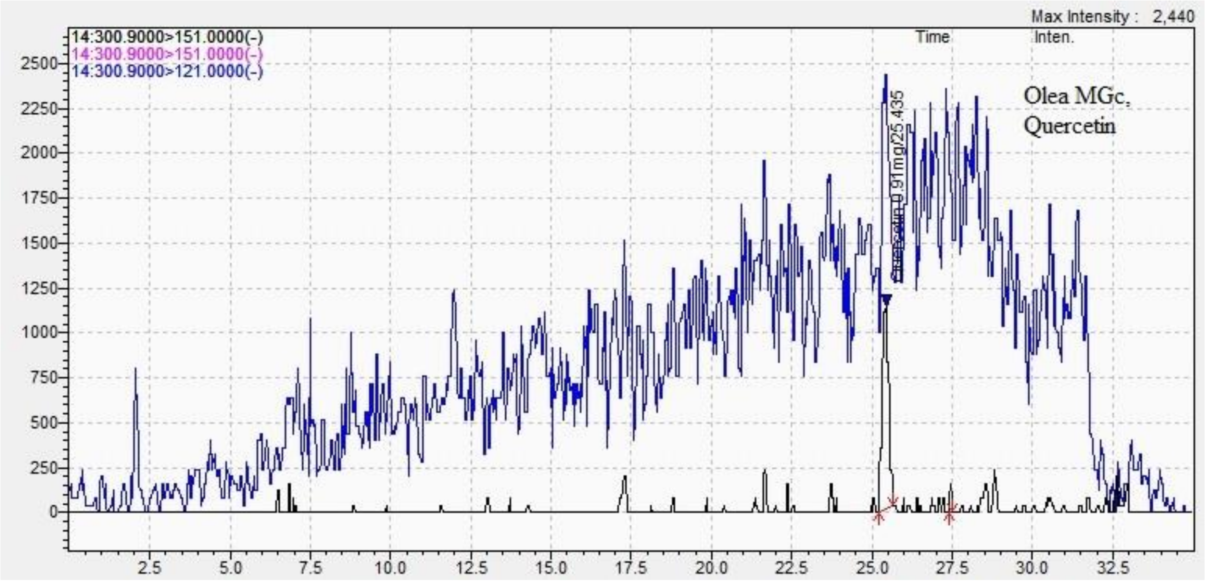

Figure S37. Chromatogram of quercetin obtained in the quantitative analysis of selected polyphenols of the O-GTE (olive)

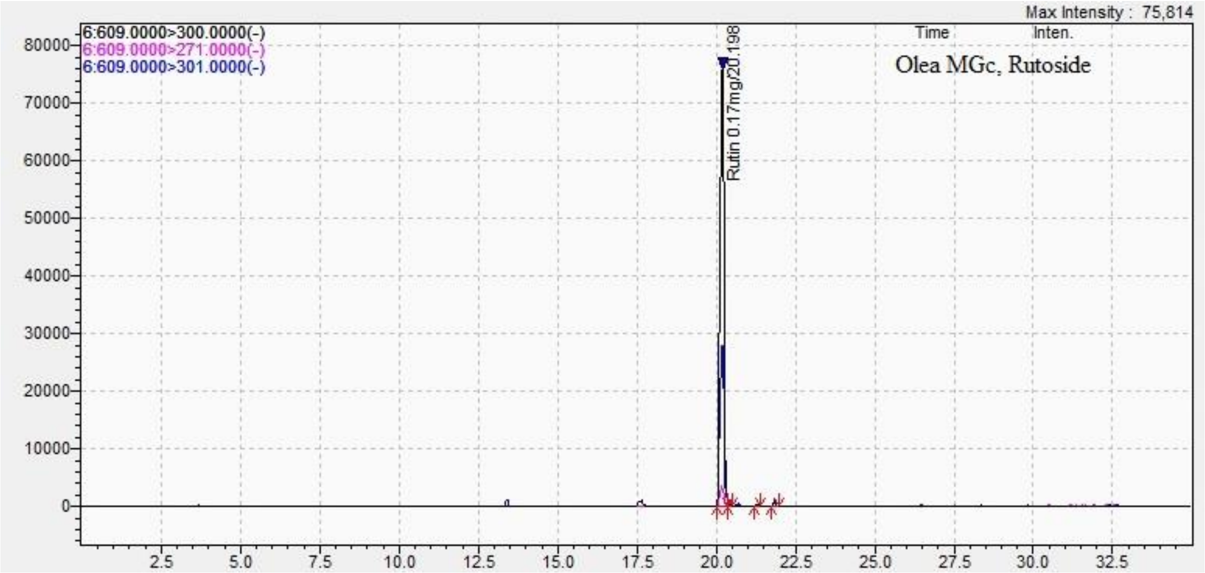

Figure S38. Chromatogram of rutoside obtained in the quantitative analysis of selected polyphenols of the O-GTE (olive)

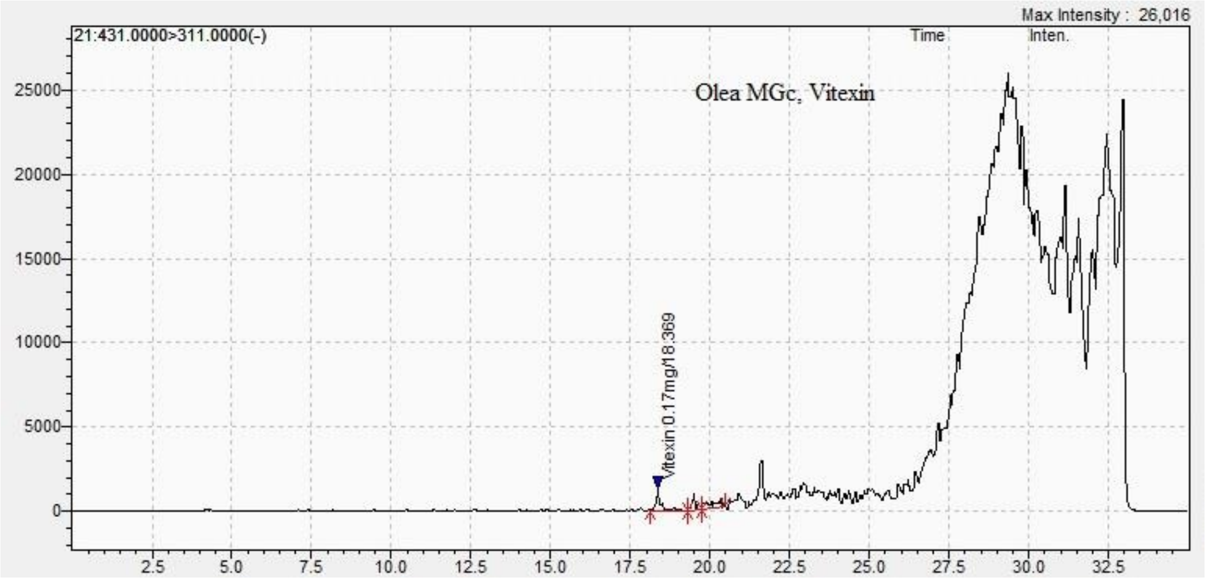

Figure S39. Chromatogram of vitexin obtained in the quantitative analysis of selected polyphenols of the O-GTE (olive)

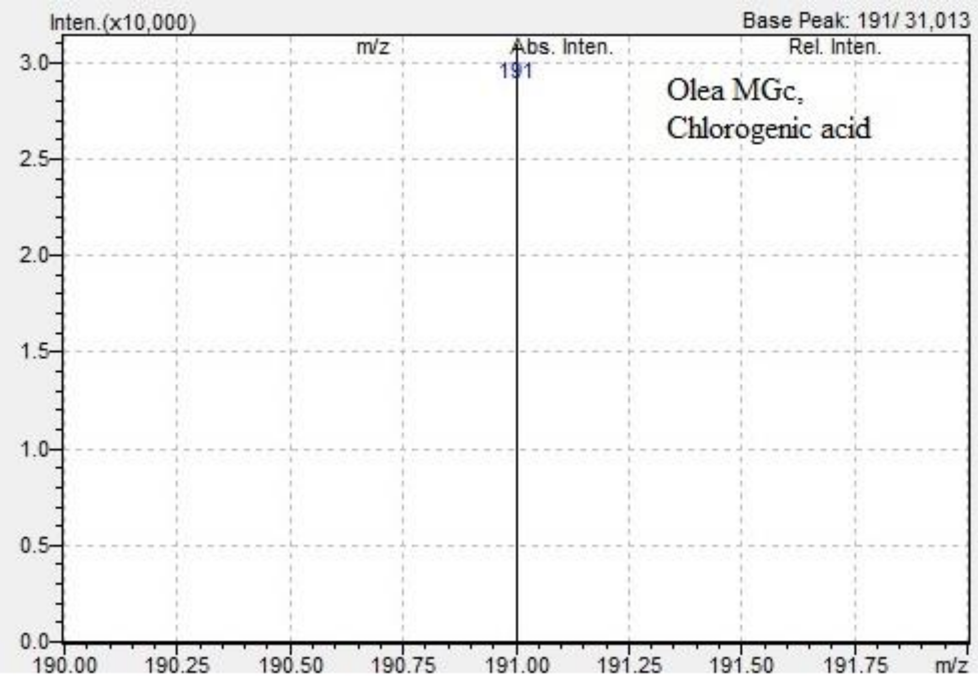

Figure S40. MS spectrum of chlorogenic acid obtained in the quantitative analysis of selected polyphenols of the O-GTE (olive)

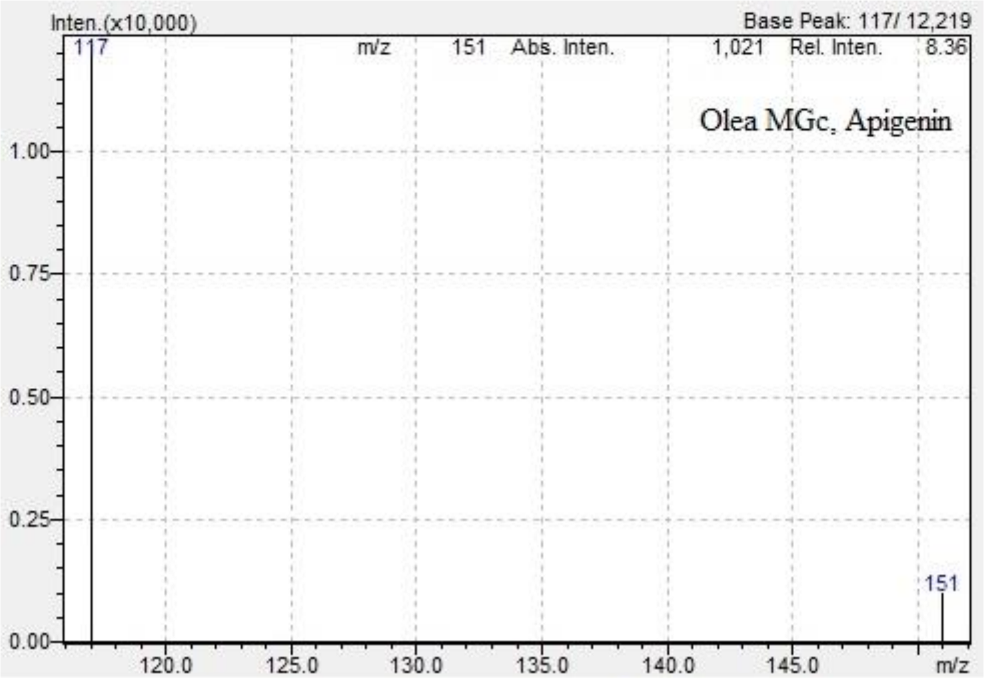

Figure S41. MS spectrum of apigenin obtained in the quantitative analysis of selected polyphenols of the O-GTE (olive)

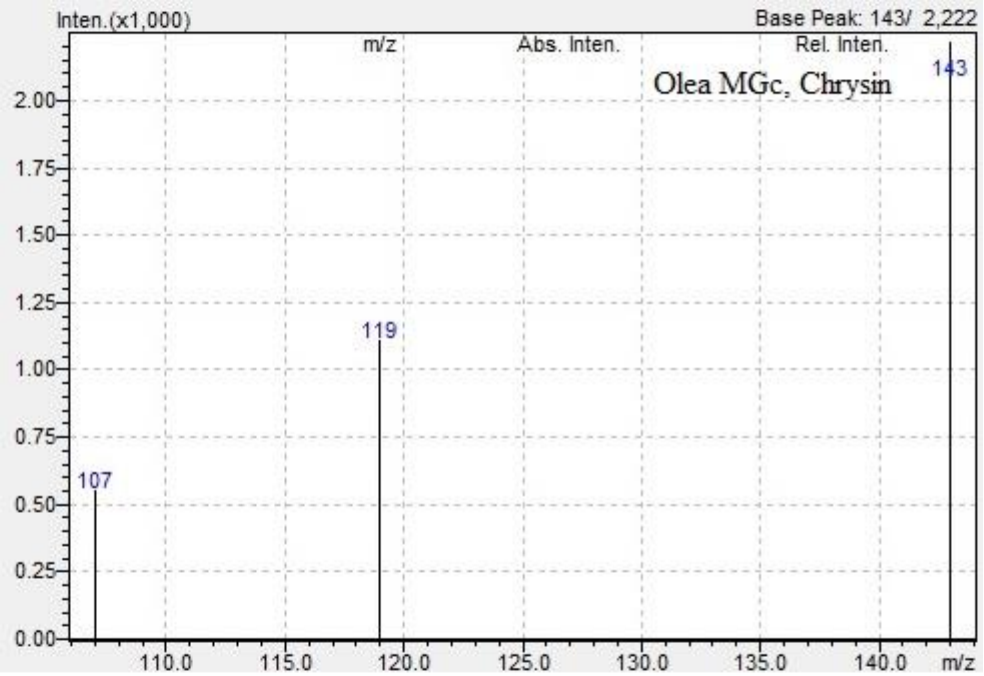

Figure S42. MS spectrum of chrysin obtained in the quantitative analysis of selected polyphenols of the O-GTE (olive)

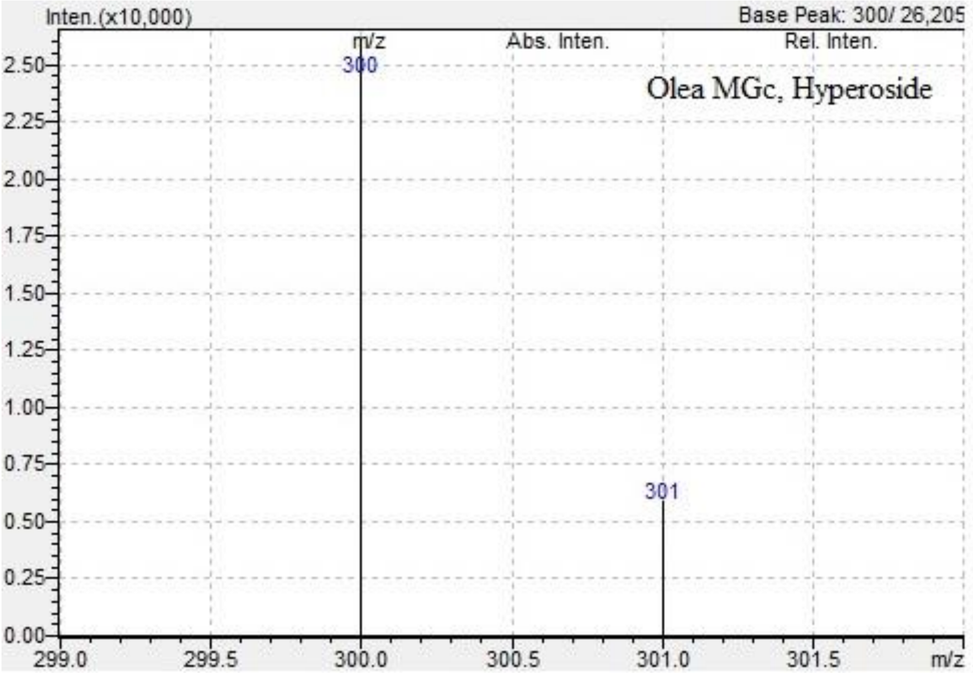

Figure S43. MS spectrum of hyperoside obtained in the quantitative analysis of selected polyphenols of the O-GTE (olive)

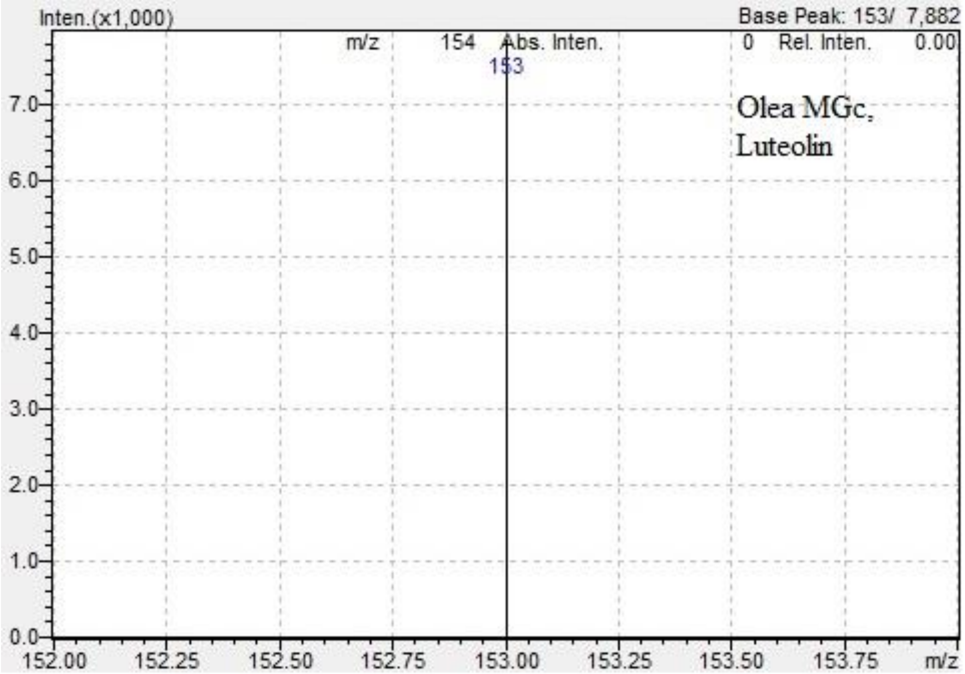

Figure S44. MS spectrum of luteolin obtained in the quantitative analysis of selected polyphenols of the O-GTE (olive)

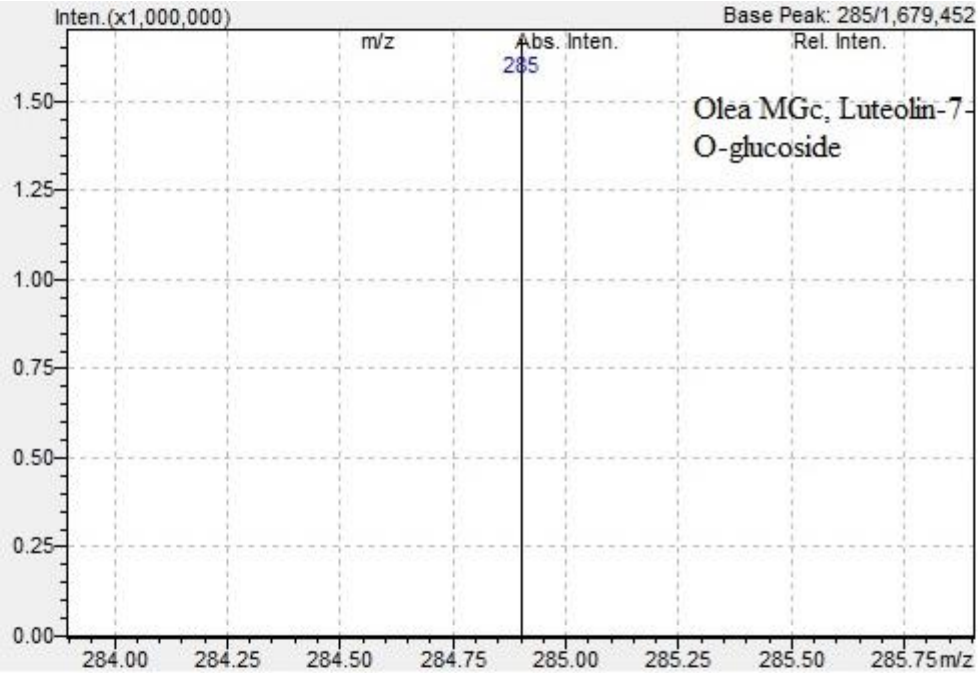

Figure S45. MS spectrum of luteolin-*7-O*-glucoside obtained in the quantitative analysis of selected polyphenols of the O-GTE (olive)

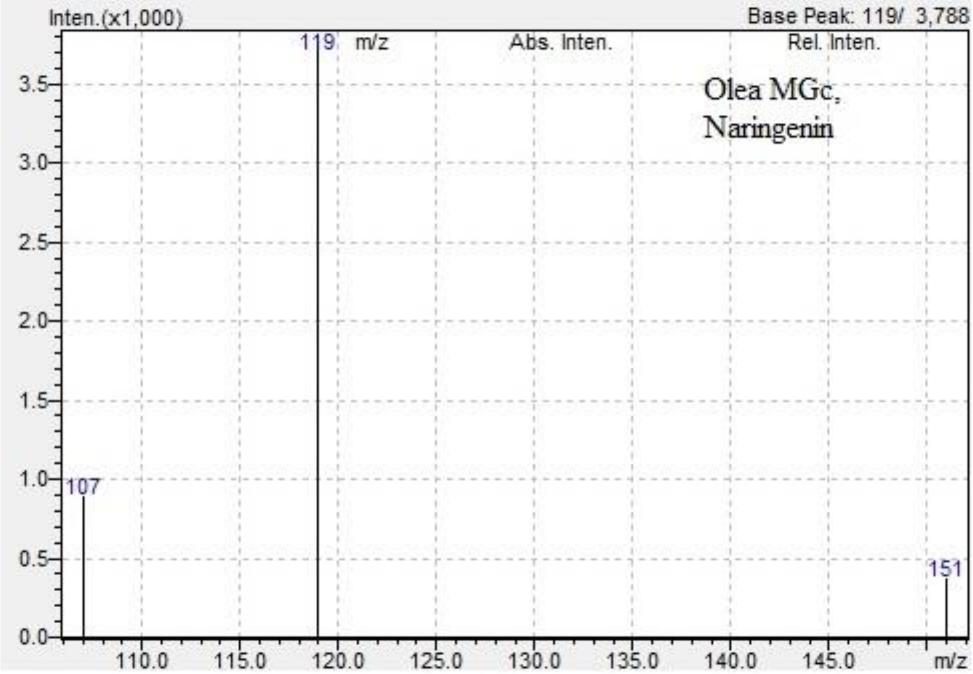

Figure S46. MS spectrum of naringenin obtained in the quantitative analysis of selected polyphenols of the O-GTE (olive)

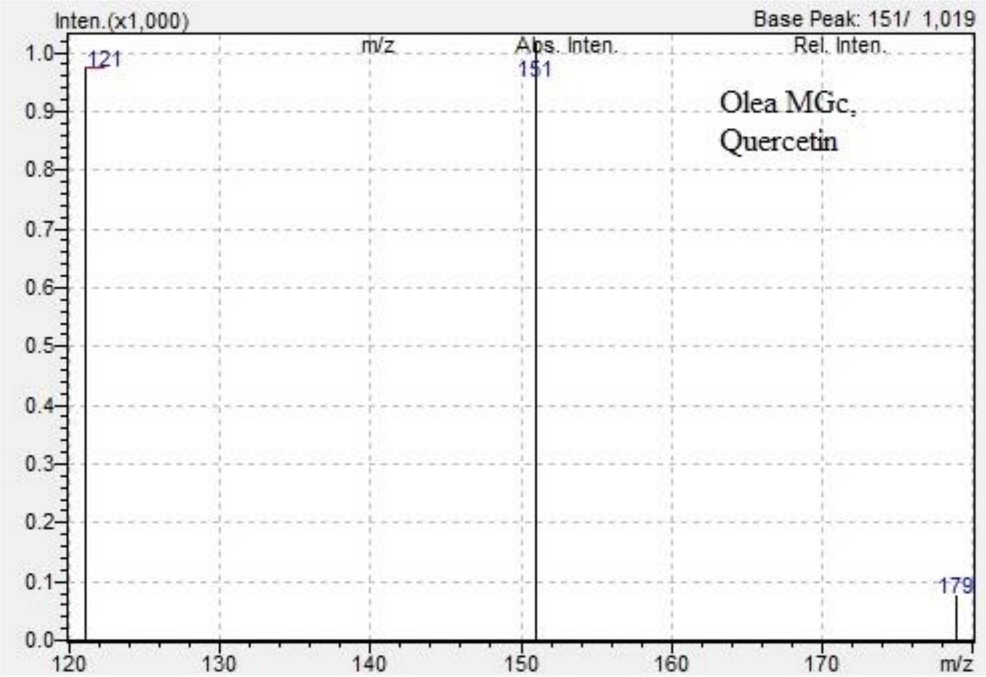

Figure S47. MS spectrum of quercetin obtained in the quantitative analysis of selected polyphenols of the O-GTE (olive)

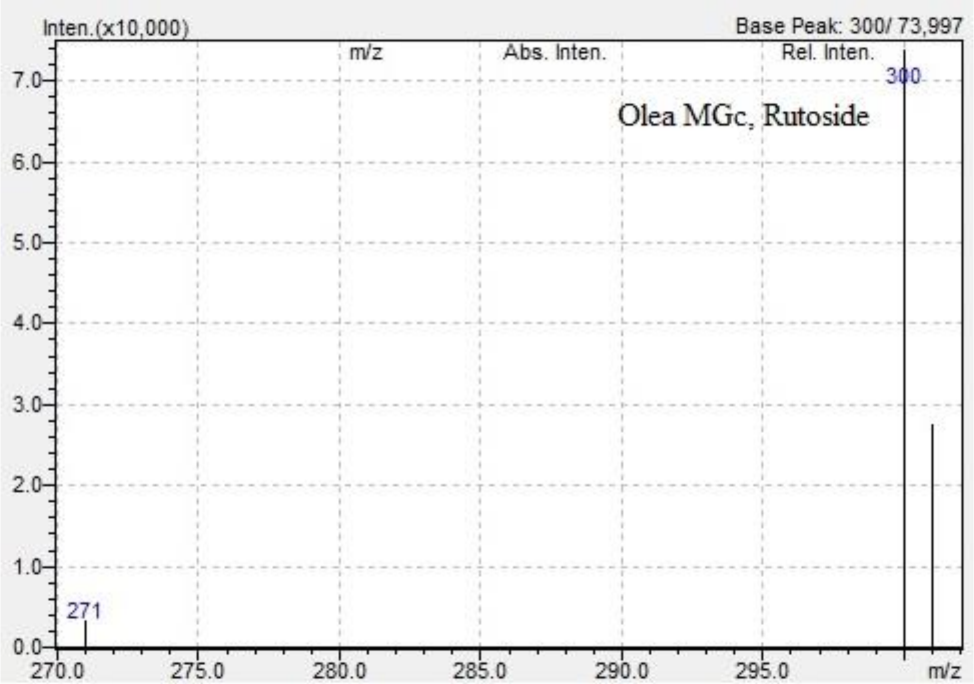

Figure S48. MS spectrum of rutoside obtained in the quantitative analysis of selected polyphenols of the O-GTE (olive)

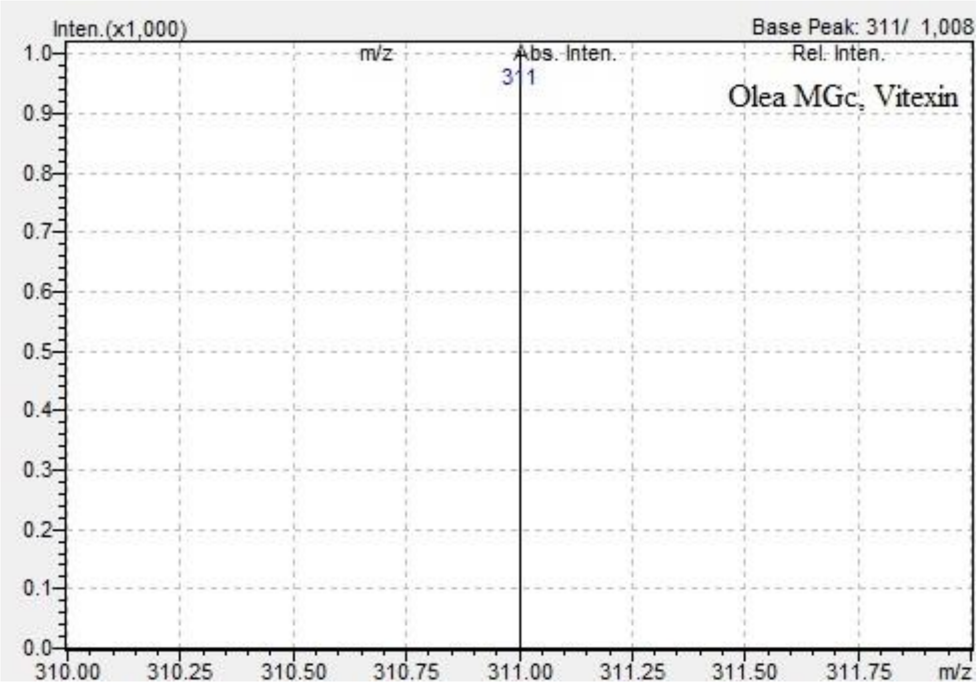

Figure S49. MS spectrum of vitexin obtained in the quantitative analysis of selected polyphenols of the O-GTE (olive)

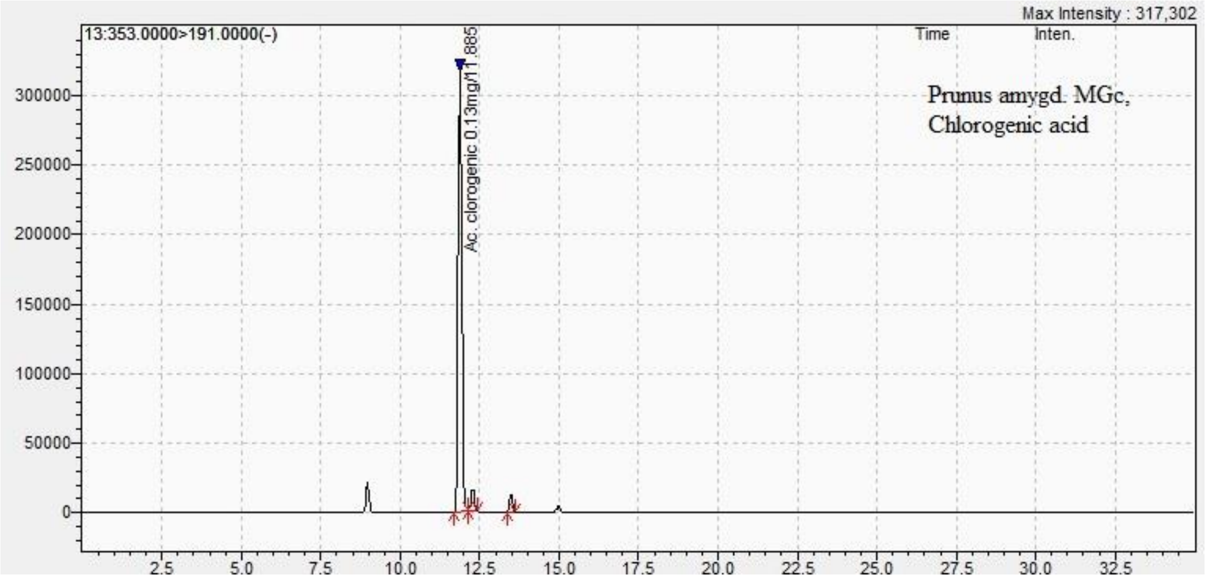

Figure S50. Chromatogram of chlorogenic acid obtained in the quantitative analysis of selected polyphenols of the SA-GTE (sweet almond)

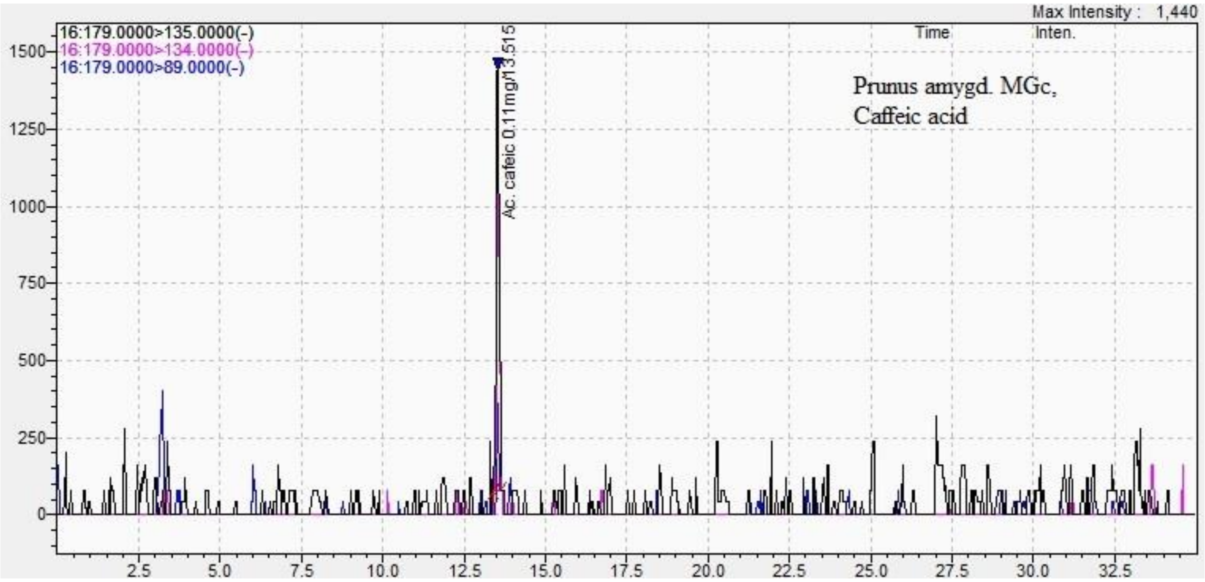

Figure S51. Chromatogram of caffeic acid obtained in the quantitative analysis of selected polyphenols of the SA-GTE (sweet almond)

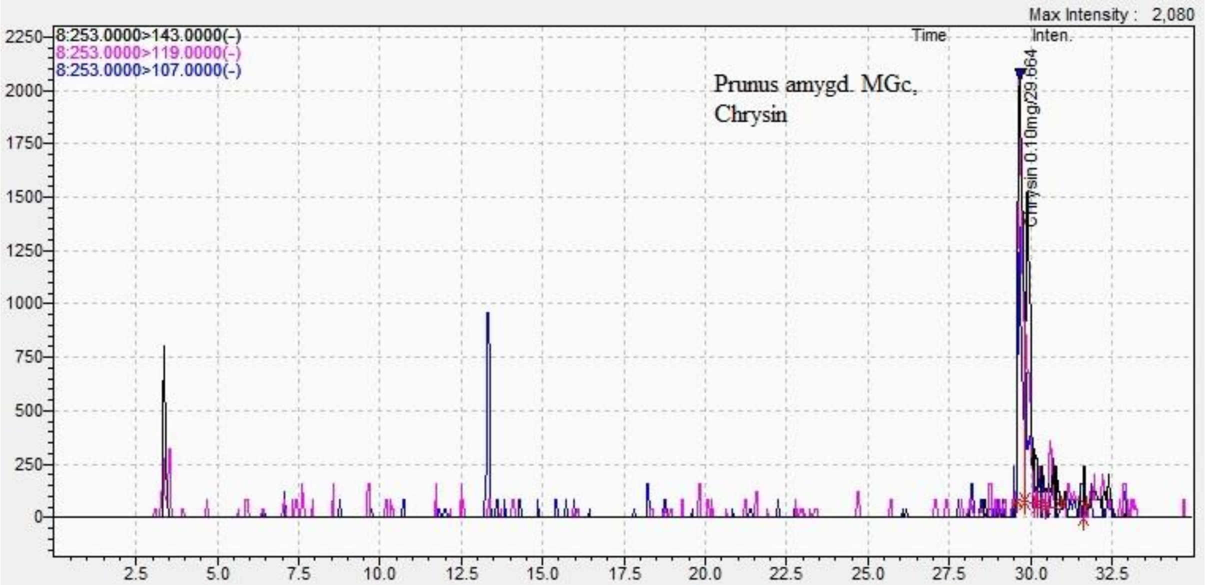

Figure S52. Chromatogram of chrysin obtained in the quantitative analysis of selected polyphenols of the SA-GTE (sweet almond)

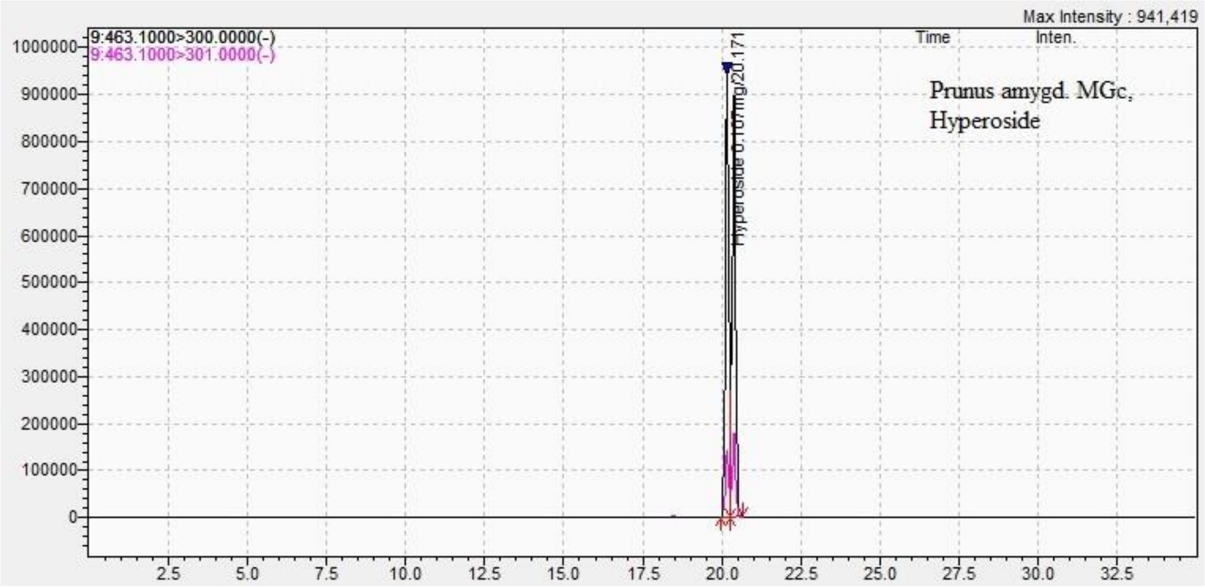

Figure S53. Chromatogram of hyperoside obtained in the quantitative analysis of selected polyphenols of the SA-GTE (sweet almond)

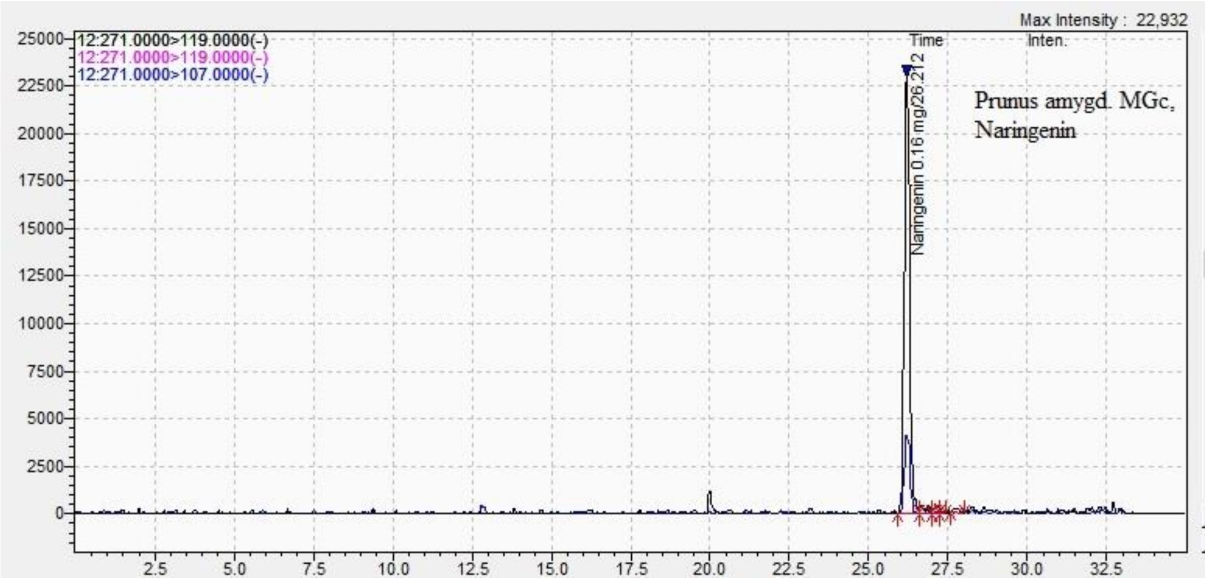

Figure S54. Chromatogram of naringenin obtained in the quantitative analysis of selected polyphenols of the SA-GTE (sweet almond)

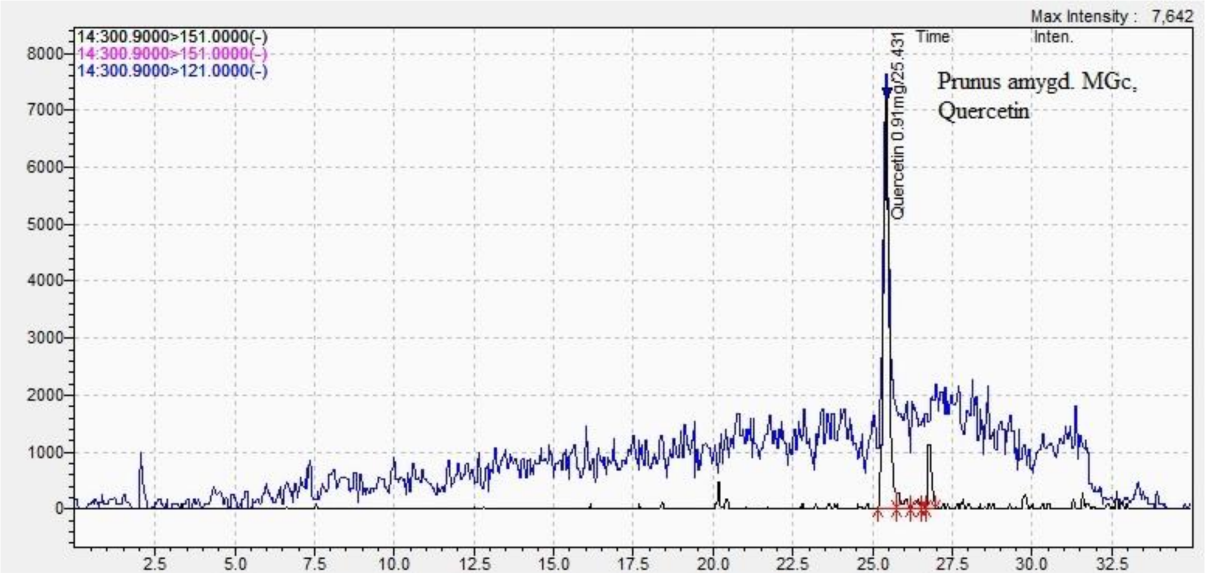

Figure S55. Chromatogram of quercetin obtained in the quantitative analysis of selected polyphenols of the SA-GTE (sweet almond)

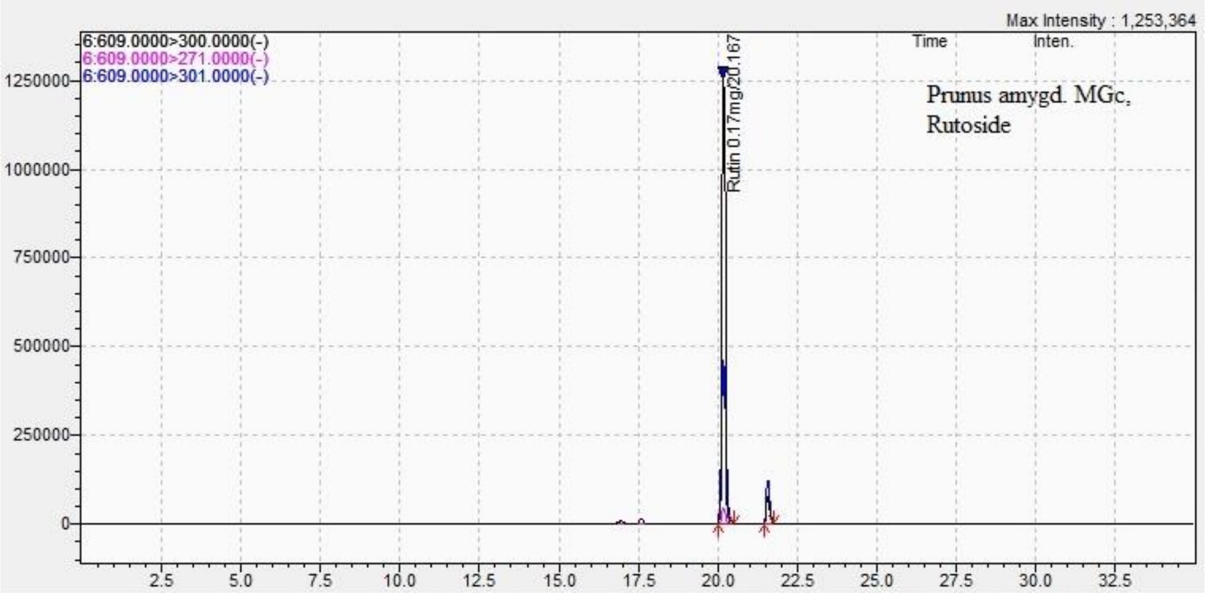

Figure S56. Chromatogram of rutoside obtained in the quantitative analysis of selected polyphenols of the SA-GTE (sweet almond)

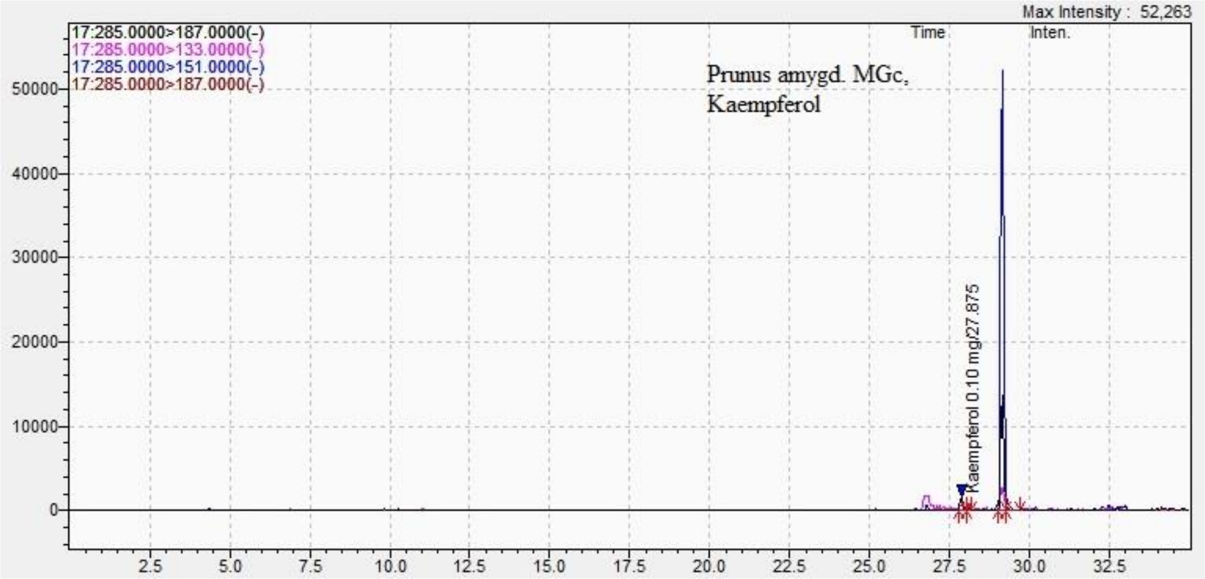

Figure S57. Chromatogram of kaempferol obtained in the quantitative analysis of selected polyphenols of the SA-GTE (sweet almond)

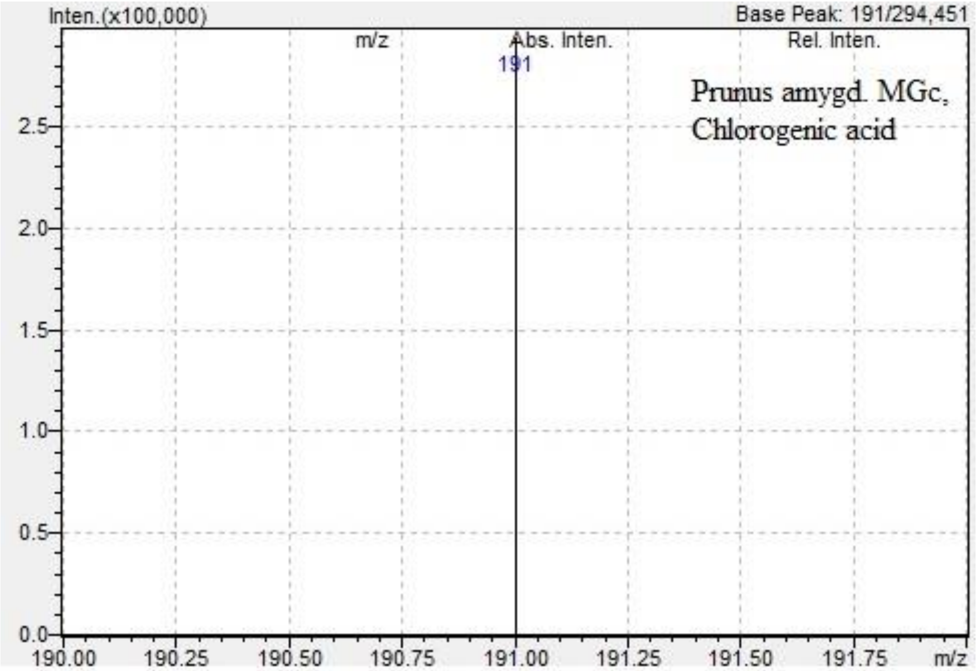

Figure S58. MS spectrum of chlorogenic acid obtained in the quantitative analysis of selected polyphenols of the SA-GTE (sweet almond)

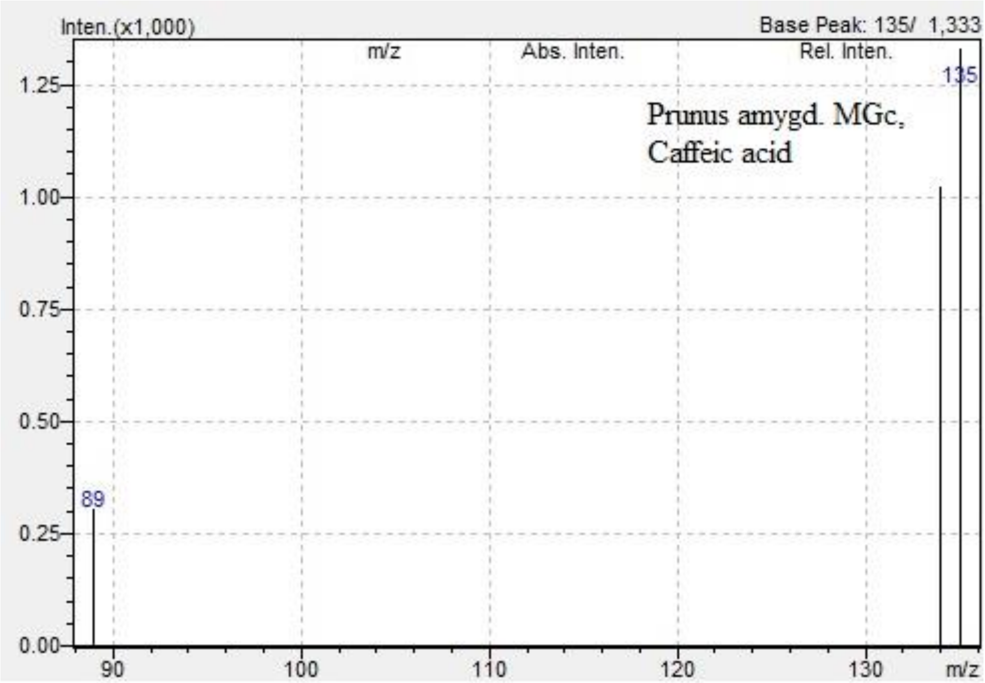

Figure S59. MS spectrum of caffeic acid obtained in the quantitative analysis of selected polyphenols of the SA-GTE (sweet almond)

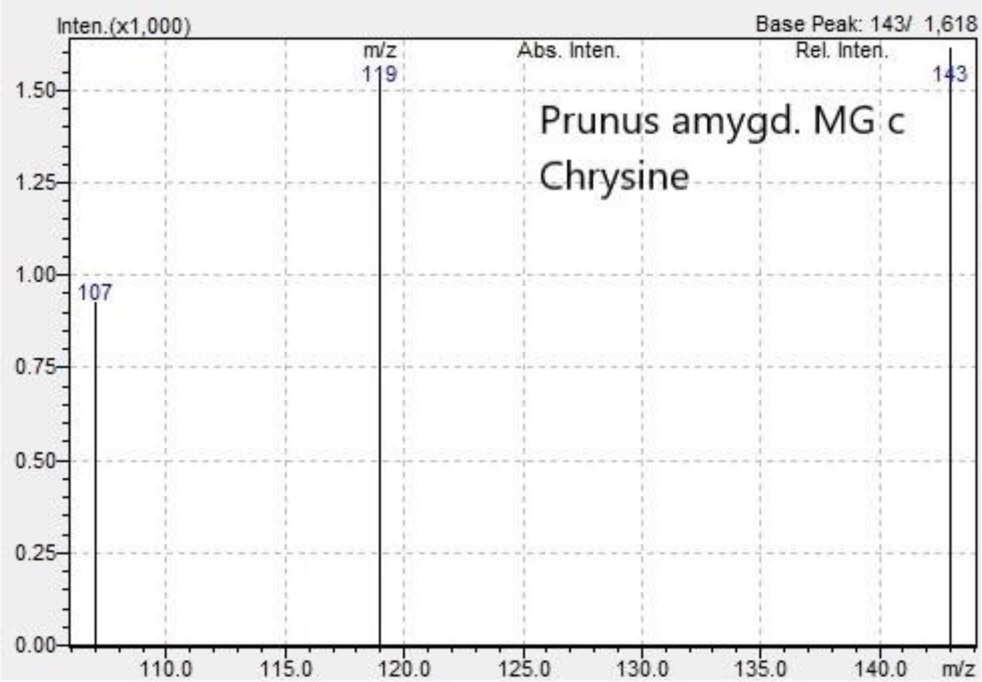

Figure S60. MS spectrum of chrysin obtained in the quantitative analysis of selected polyphenols of the SA-GTE (sweet almond)

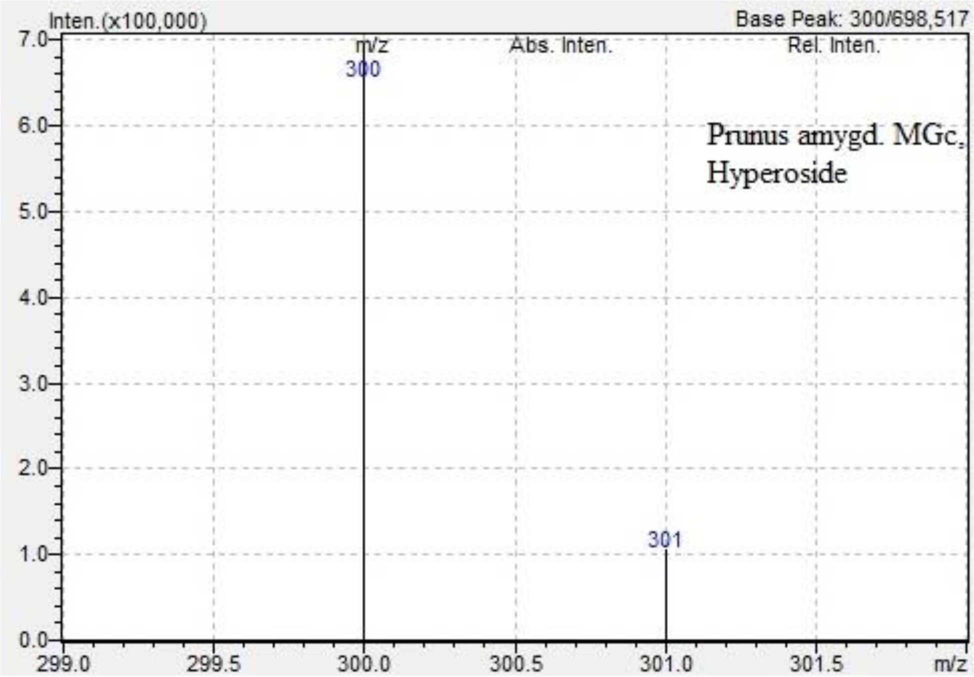

Figure S61. MS spectrum of hyperoside obtained in the quantitative analysis of selected polyphenols of the SA-GTE (sweet almond)

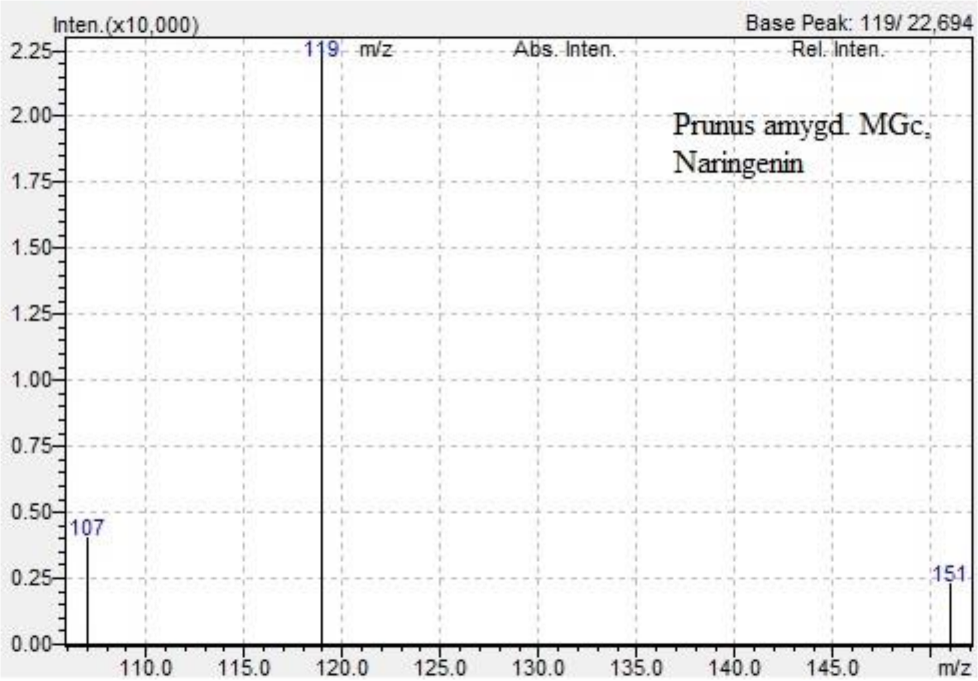

Figure S62. MS spectrum of naringenin obtained in the quantitative analysis of selected polyphenols of the SA-GTE (sweet almond)

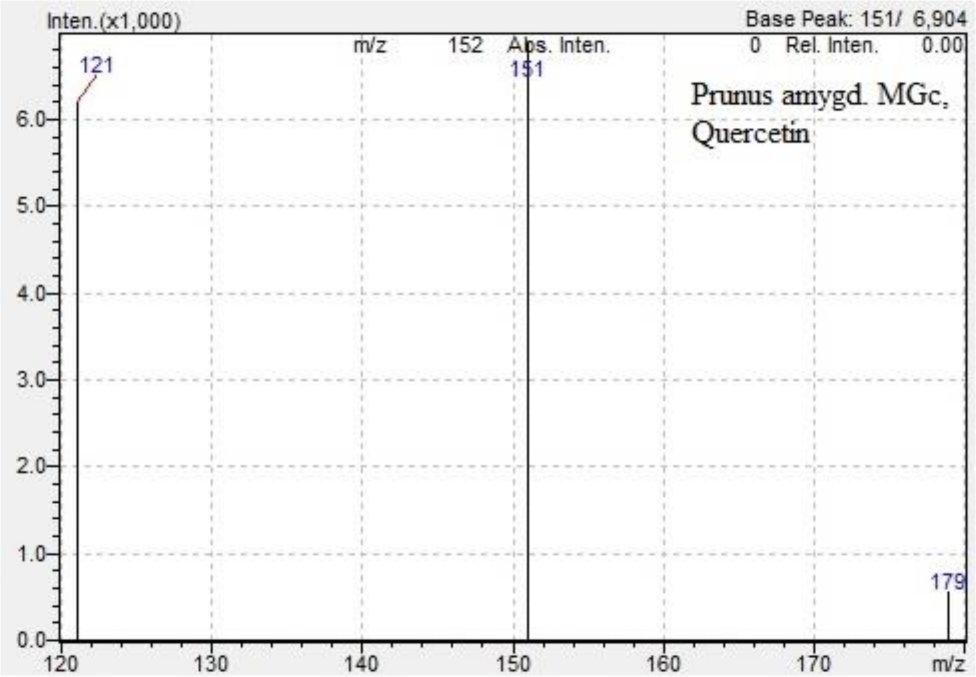

Figure S63. MS spectrum of quercetin obtained in the quantitative analysis of selected polyphenols of the SA-GTE (sweet almond)

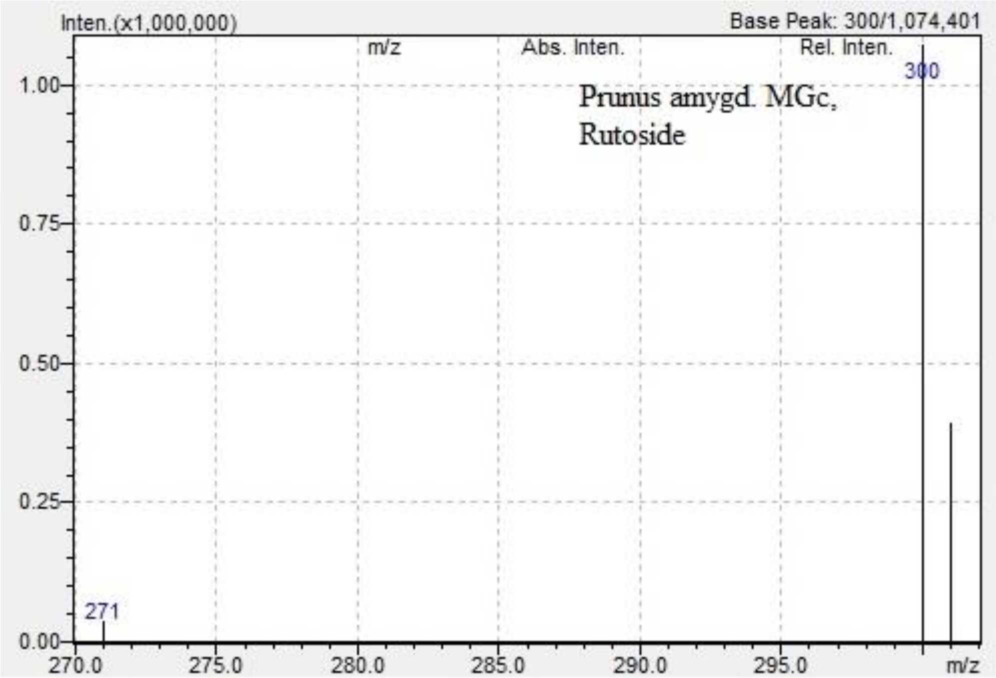

Figure S64. MS spectrum of rutoside obtained in the quantitative analysis of selected polyphenols of the SA-GTE (sweet almond)

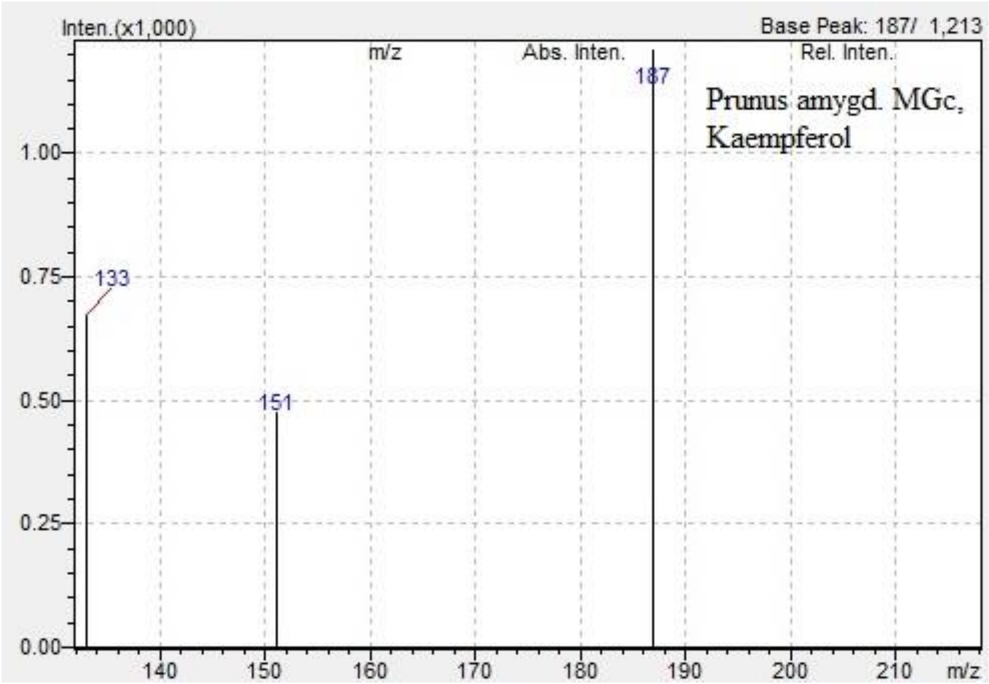

Figure S65. MS spectrum of kaempferol obtained in the quantitative analysis of selected polyphenols of the SA-GTE (sweet almond)

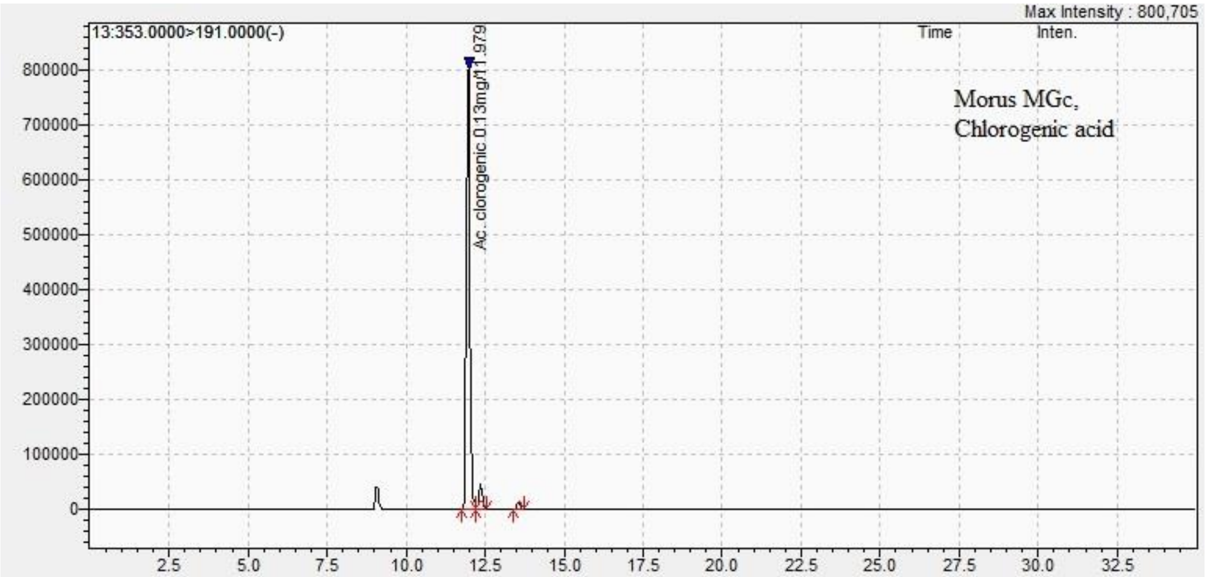

Figure S66. Chromatogram of chlorogenic acid obtained in the quantitative analysis of selected polyphenols of the BM-GTE (black mulberry)

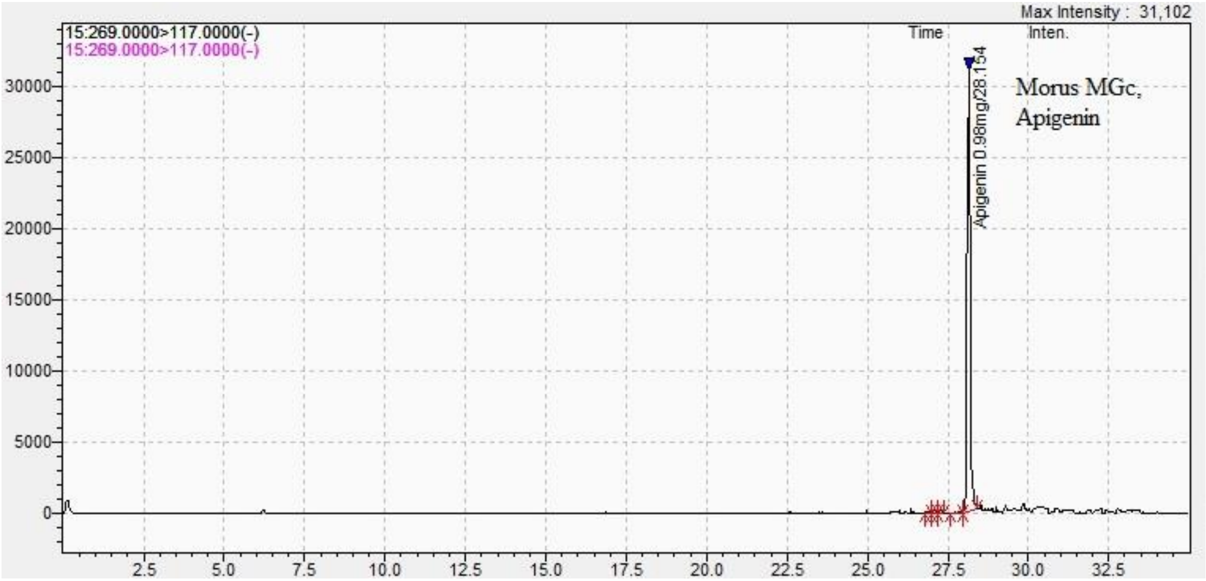

Figure S67. Chromatogram of apigenin obtained in the quantitative analysis of selected polyphenols of the BM-GTE (black mulberry)

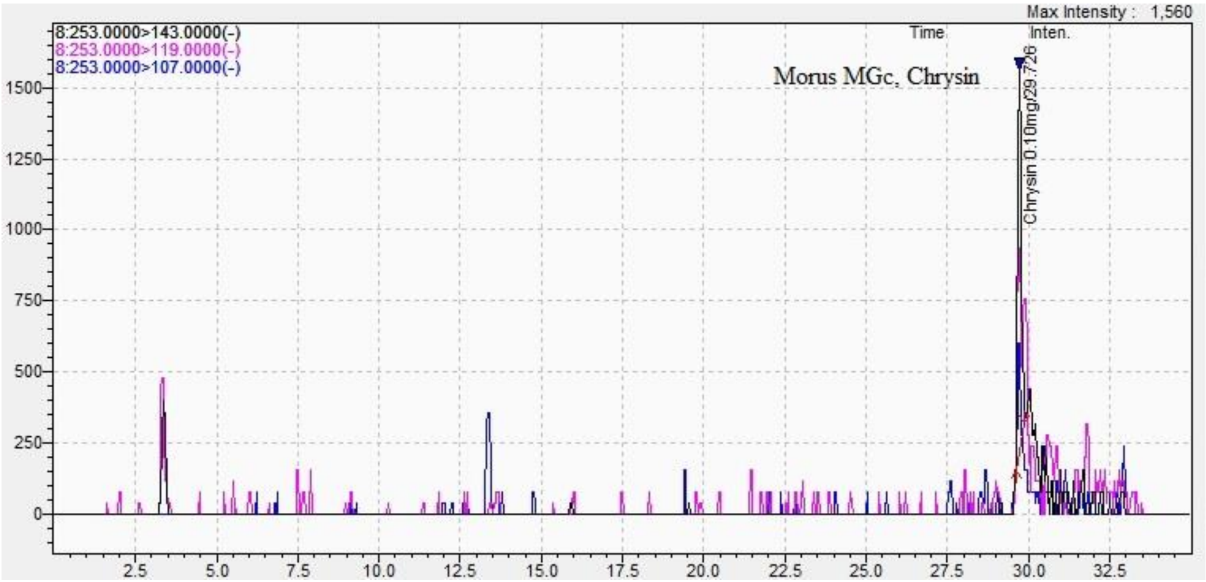

Figure S68. Chromatogram of chrysin obtained in the quantitative analysis of selected polyphenols of the BM-GTE (black mulberry)

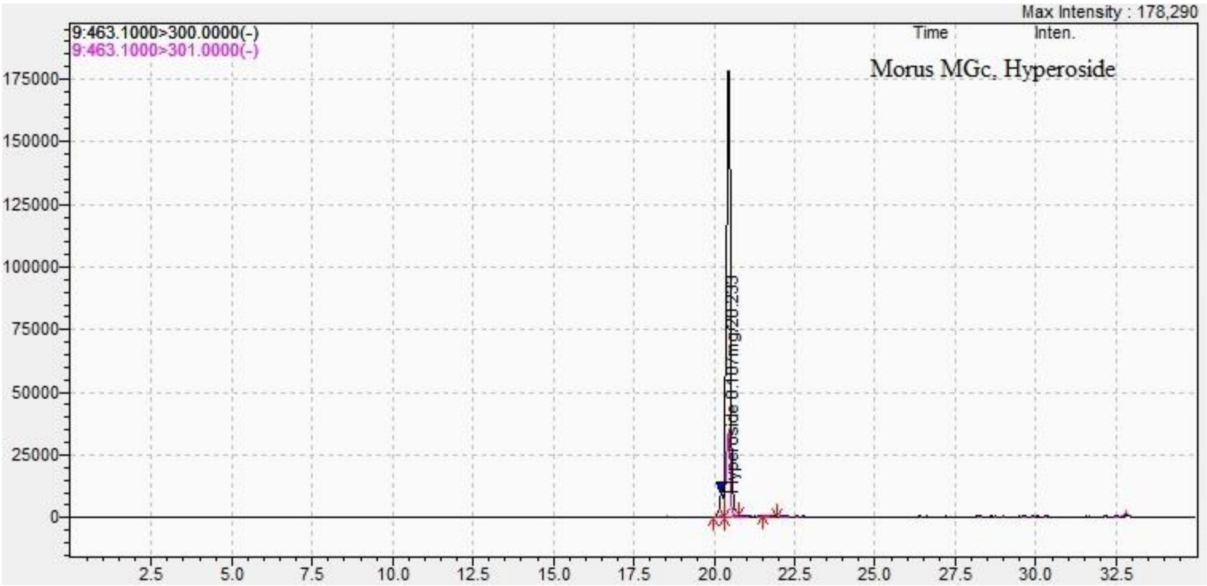

Figure S69. Chromatogram of hyperoside obtained in the quantitative analysis of selected polyphenols of the BM-GTE (black mulberry)

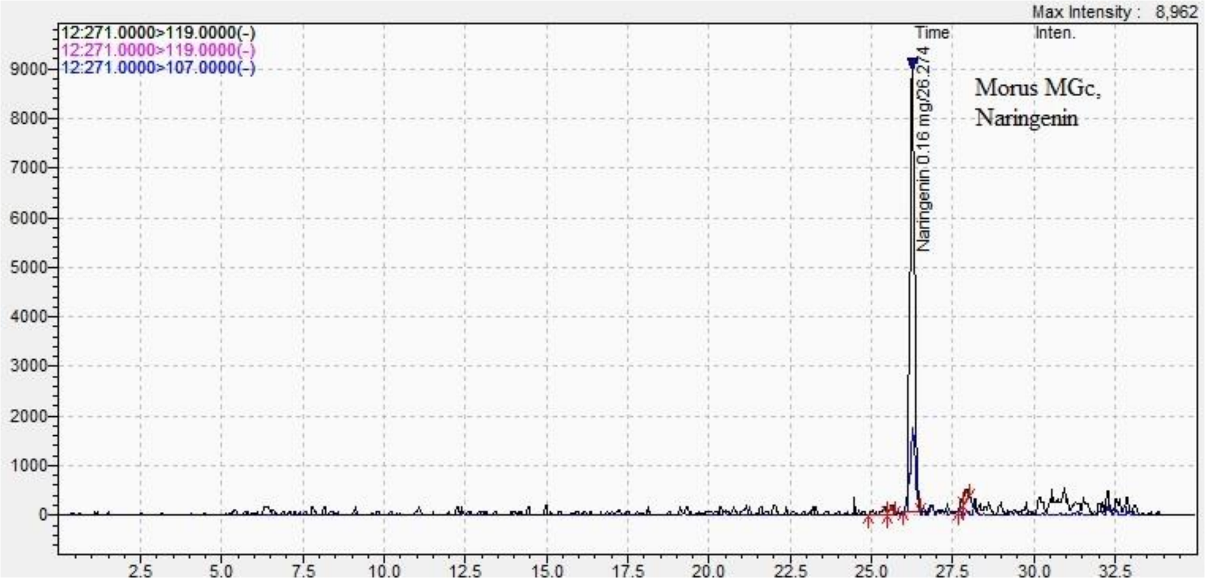

Figure S70. Chromatogram of naringenin obtained in the quantitative analysis of selected polyphenols of the BM-GTE (black mulberry)

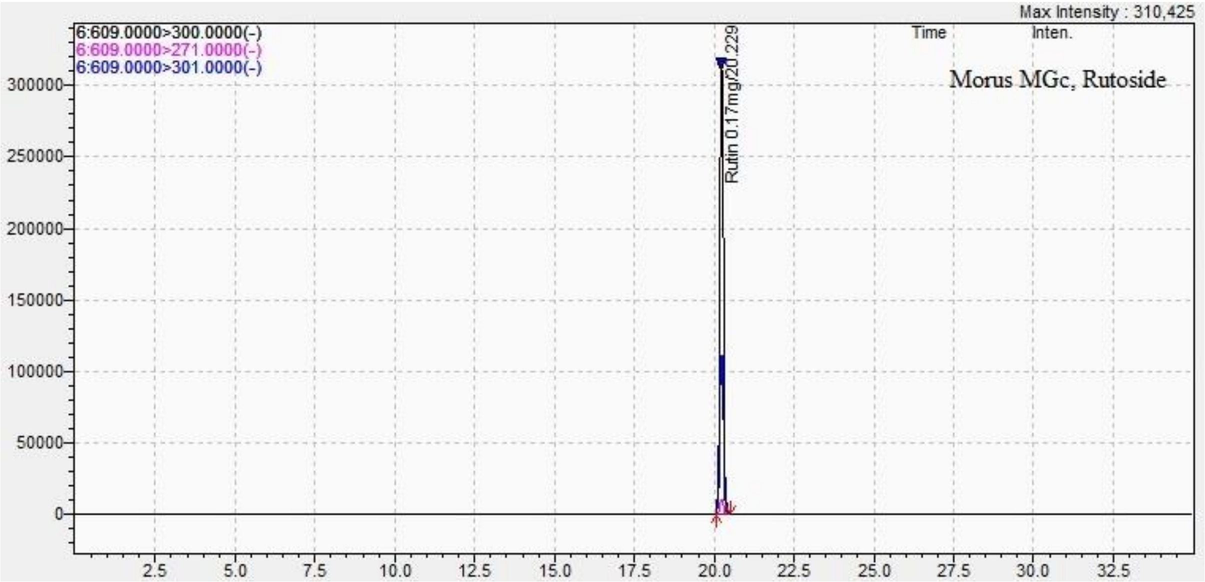

Figure S71. Chromatogram of rutoside obtained in the quantitative analysis of selected polyphenols of the BM-GTE (black mulberry)

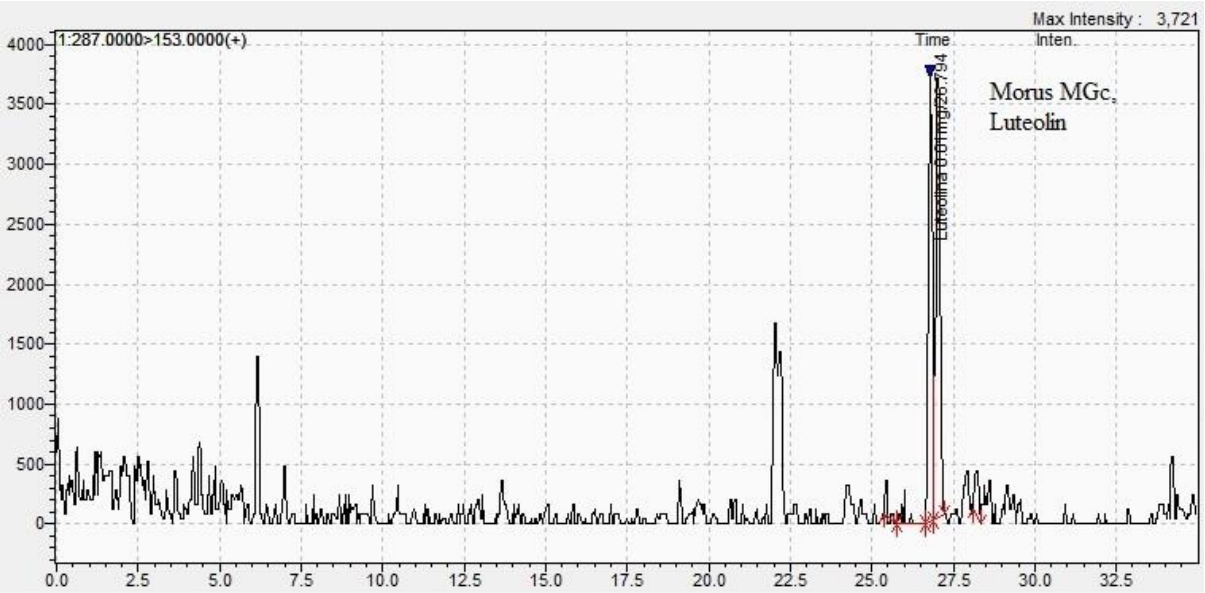

Figure S72. Chromatogram of luteolin obtained in the quantitative analysis of selected polyphenols of the BM-GTE (black mulberry)

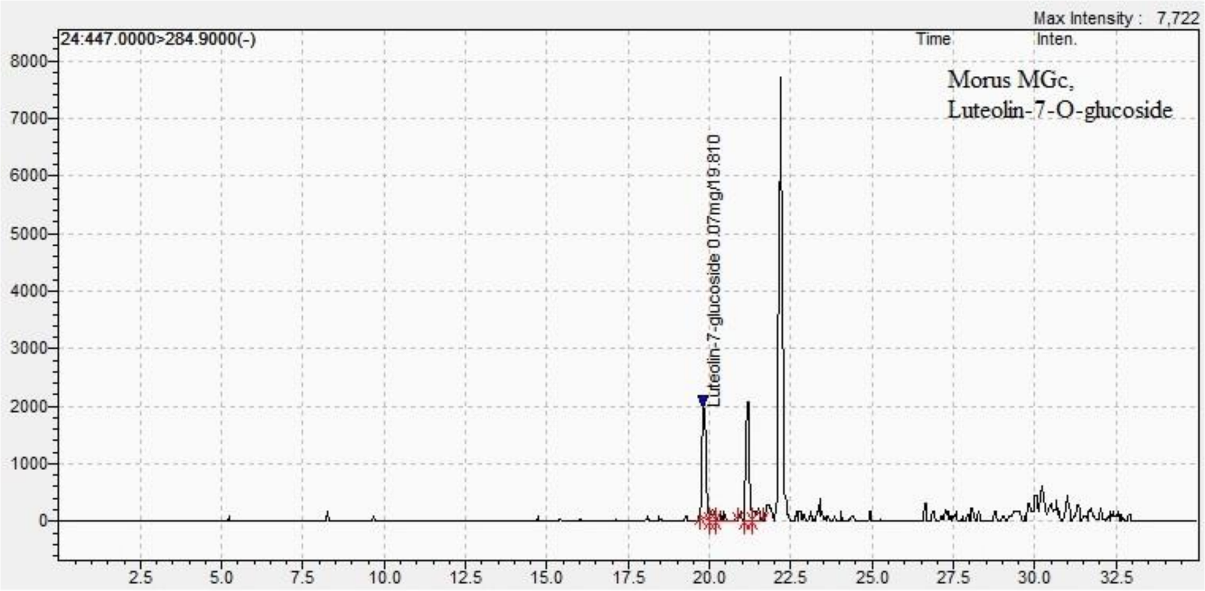

Figure S73. Chromatogram of luteolin-*7-o*-glucoside obtained in the quantitative analysis of selected polyphenols of the BM-GTE (black mulberry)

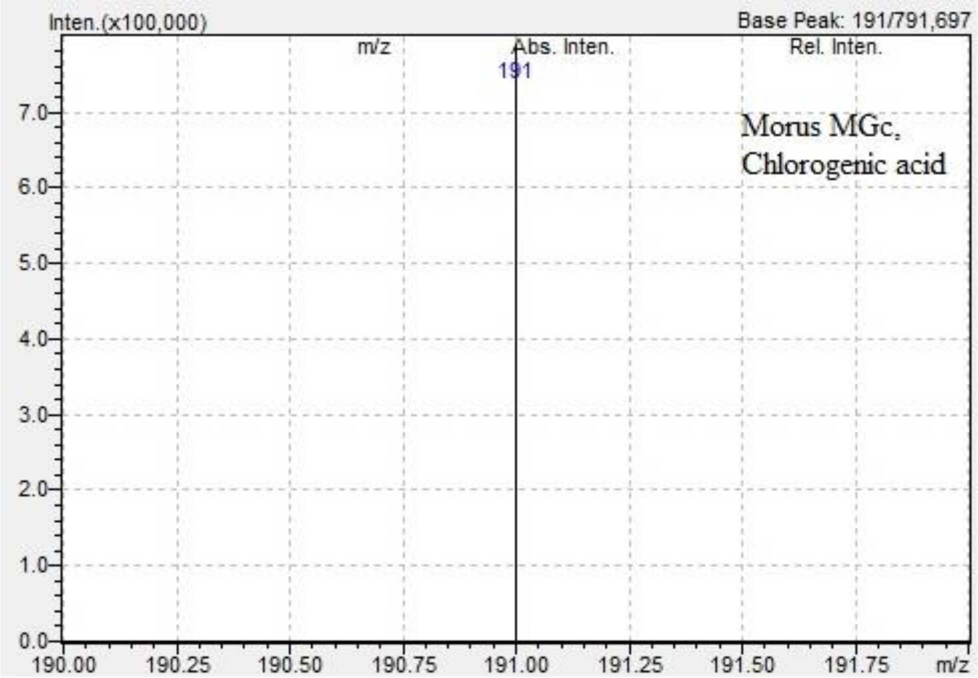

Figure S74. MS spectrum of chlorogenic acid obtained in the quantitative analysis of selected polyphenols of the BM-GTE (black mulberry)

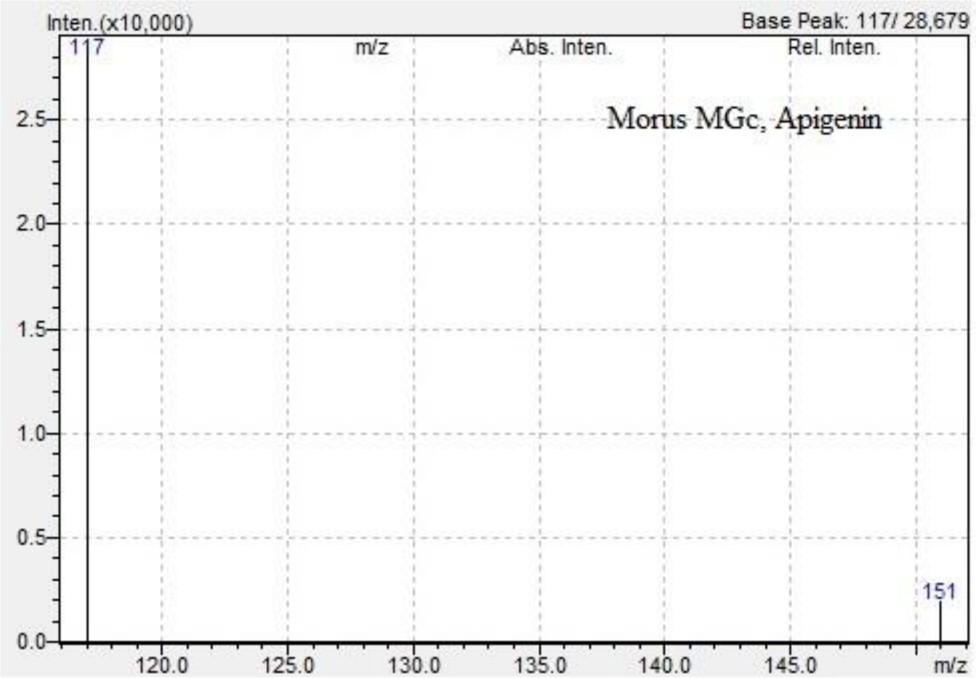

Figure S75. MS spectrum of apigenin obtained in the quantitative analysis of selected polyphenols of the BM-GTE (black mulberry)

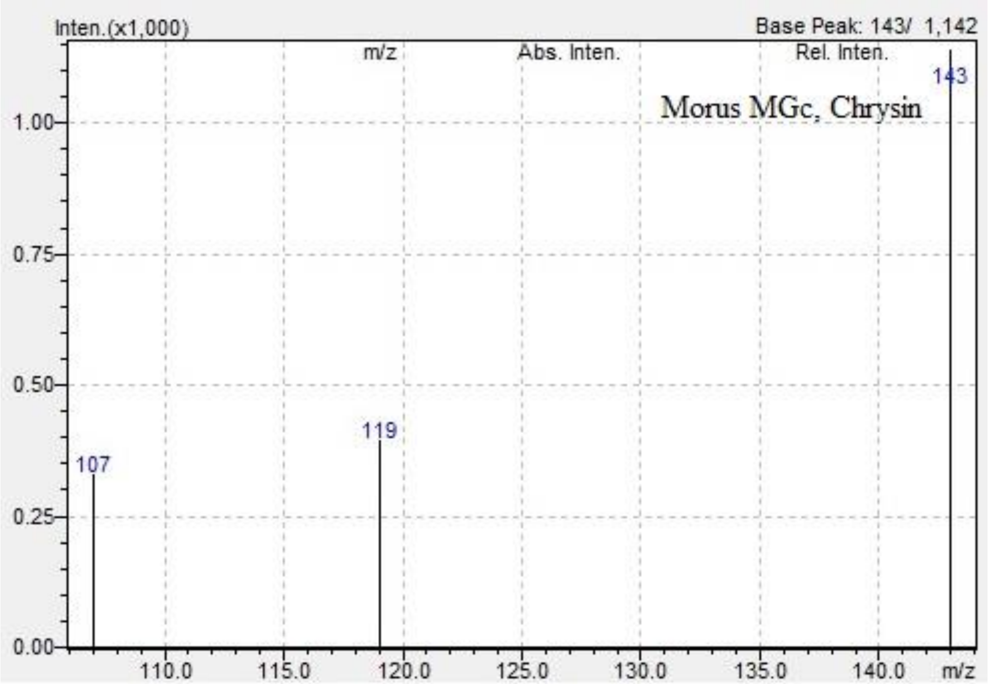

Figure S76. MS spectrum of chrysin obtained in the quantitative analysis of selected polyphenols of the BM-GTE (black mulberry)

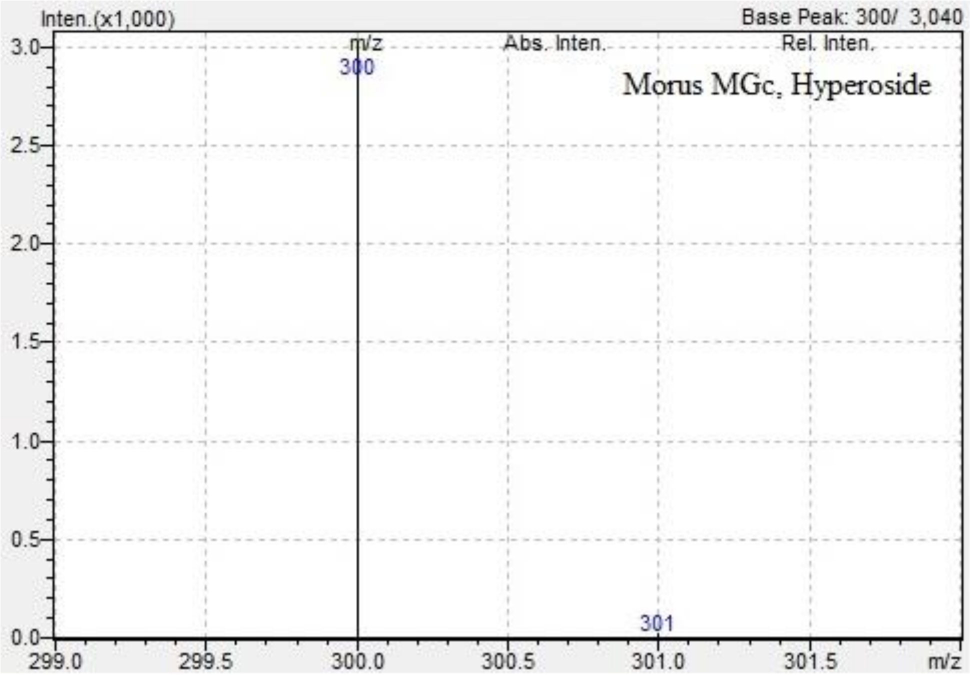

Figure S77. MS spectrum of hyperoside obtained in the quantitative analysis of selected polyphenols of the BM-GTE (black mulberry)

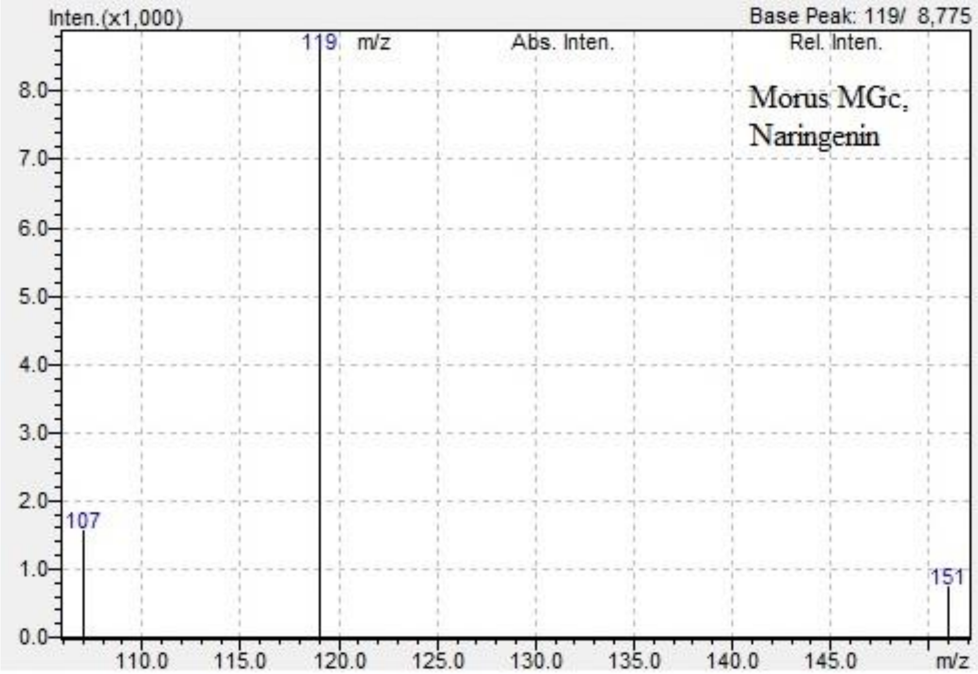

Figure S78. MS spectrum of naringenin obtained in the quantitative analysis of selected polyphenols of the BM-GTE (black mulberry)

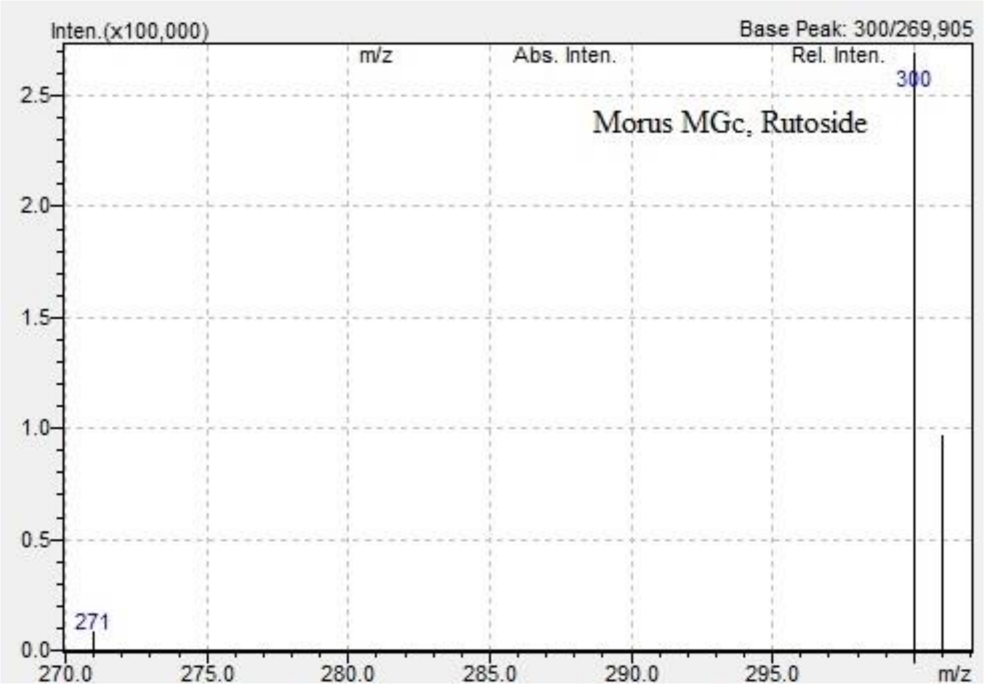

Figure S79. MS spectrum of rutoside obtained in the quantitative analysis of selected polyphenols of the BM-GTE (black mulberry)

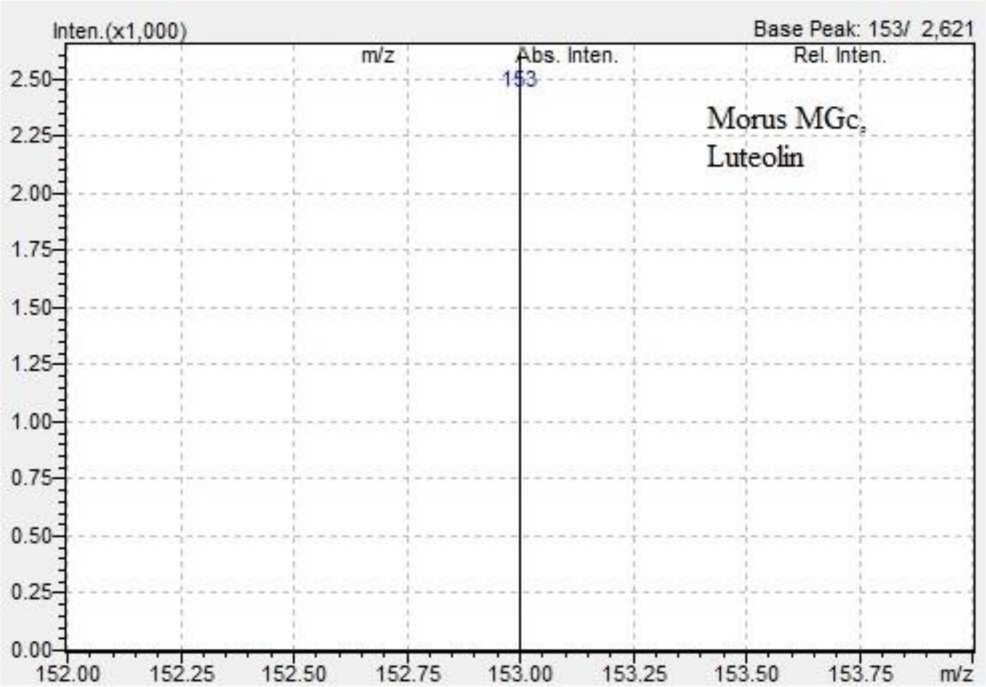

Figure S80. MS spectrum of luteolin obtained in the quantitative analysis of selected polyphenols of the BM-GTE (black mulberry)

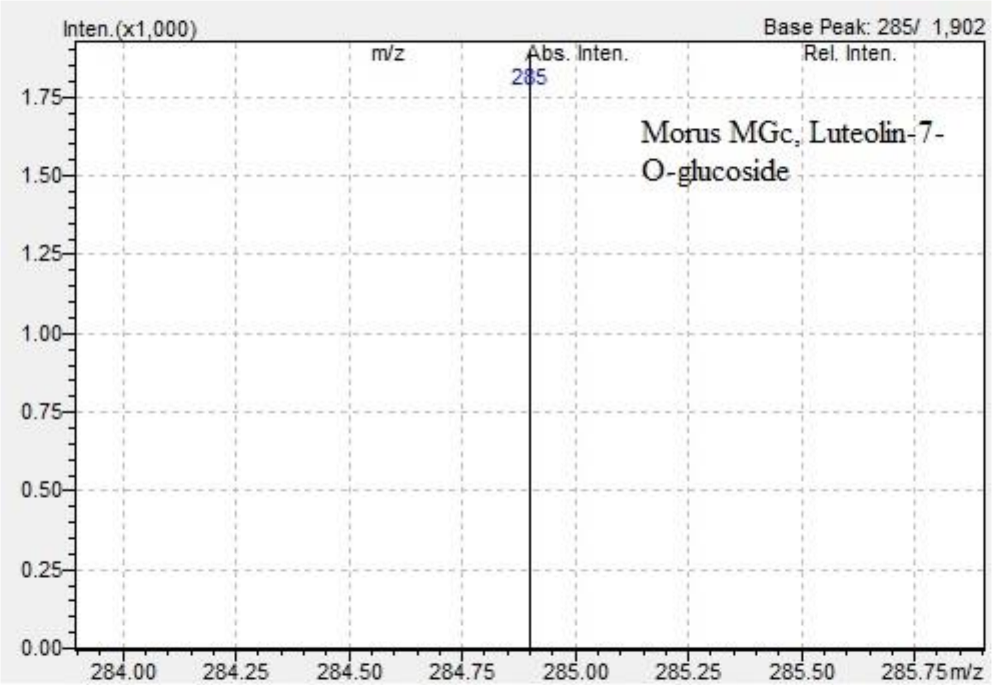

Figure S81. MS spectrum of luteolin-*7-o*-glucoside obtained in the quantitative analysis of selected polyphenols of the BM-GTE (black mulberry)

Table S1. Protocol of the quantitative analysis of GTE specific selected polyphenols by UHPLC-ESI-MS

Time, min	Methanol	Water	2 % formic acid in water
0.00	5	90	5
3.00	15	70	15
6.00	15	70	15
9.00	21	58	21
13.00	21	58	21
18.00	30	41	29
22.00	30	41	29
26.00	50	0	50
29.00	50	0	50
29.01	5	90	5
35.00	5	90	5

Table S2. The phytonutrient profile of O-GTE (olive)

No.	Compound type	Phytonutrient	RT	M [+]	M [-]
1	Vitamin	Adenine (B4)	1.29	136.06233	
2	Vitamin	Nicotinamide	1.40	123.05584	
3	Vitamin	Nicotinic acid (Niacin,B3)	1.40	124.03986	
4	Polyphenol	Hydroxytyrosol (3,4-Dihydroxyphenylethanol)	5.34		153.05517
5	Polyphenol	Dihydroxycoumarin-O-hexoside	12.24	341.08726	
6	Carboxylic acid	Kynurenic acid	13.18	190.05042	
7	Polyphenol	Esculetin (6,7-dihydroxycoumarin)	14.04		179.03444
8	Polyphenol	Chlorogenic acid (3-O-Caffeoylquinic acid)	14.28	355.10291	
9	Iridoid	Oleoside	16.88		389.10839
10	Flavonoid	Quercetin-di-O-hexoside	17.17		625.14048
11	Carboxylic acid	12-Hydroxyjasmonic acid glucoside or Tuberonic acid glucoside	17.59		387.16551
12	Carboxylic acid	12-Hydroxyjasmonic acid or Tuberonic acid	18.26		225.11269
13	Polyphenol	Scopoletin (7-Hydroxy-6-methoxycoumarin)	18.55	193.05009	
14	Flavonoid	Taxifolin (Dihydroquercetin)	19.32		303.05048
15	Iridoid	Neonuzhenide	21.54		701.22929
16	Iridoid	Nuzhenide dihydroxyphenylacetic acid isomer	21.66		715.20856
17	Flavonoid	Dihydrokaempferol (Aromadendrin, Katuranin)	21.92		287.05557
18	Polyphenol	Verbascoside	22.01		623.19760
19	Flavonoid	Luteolin-7-O-glucoside (Cynaroside)	22.36		447.09274
20	Flavonoid	Luteolin-O-rutinoside isomer	22.45		593.15065
21	Lignan Polyphenol	8-Acetoxypinoresinol-4-O-glucoside	22.75		577.19212
22	Iridoid	Oleuropein hexoside isomer 1	22.89		701.22929
23	Flavonoid	Isoquercitrin (Hirsutrin, Quercetin-3-O-glucoside)	22.93		463.08765
24	Flavonoid	Rutin (Quercetin-3-O-rutinoside)	23.00	611.16122	
25	Flavonoid	Luteolin-7-O-rutinoside (Scolymoside)	23.07		593.15065
26	Iridoid	Nuzhenide	23.07		685.23438

27	Iridoid	Oleuropein hexoside isomer 2	23.32		701.22929
28	Lignan<br>Polyphenol	8-Hydroxypinoresinol-4-O or 4'-O-glucoside	23.37		535.18156
29	Flavonoid	Cosmosiin (Apigetrin, Apigenin-7-O-glucoside)	23.98	433.11347	
30	Iridoid	Oleuropein hexoside isomer 3	24.07		701.22929
31	Flavonoid	Chrysoeriol-O-hexoside	24.25		461.10839
32	Iridoid	Oleuropein	24.57		539.17647
33	Flavonoid	Luteolin-3'-O-glucoside or Luteolin-5-O-glucoside	24.67		447.09274
34	Flavonoid	Luteolin-4'-O-glucoside	25.89		447.09274
35	Iridoid	Ligstroside (Ligustroside)	26.44		523.18156
36	Flavonoid	Quercetin	26.99	303.05048	
37	Flavonoid	Naringenin	27.21		271.06065
38	Flavonoid	Luteolin (3',4',5,7-Tetrahydroxyflavone)	27.85		285.03991
39	Flavonoid	Scutellarein-7-O-(6-O-feruloyl)glucoside	28.79		623.14009
40	Flavonoid	Apigenin	29.67		269.04450
41	Flavonoid	Isorhamnetin	29.84		315.05048
42	Flavonoid	Chrysoeriol	29.90		299.05556
43	Flavonoid	Pinocembrin (5,7-Dihydroxyflavanone)	32.25		255.06573
44	Terpenoid	Uvaol	45.11	443.38891	
45	Carboxylic acid	Ginkgoic acid	47.40		345.24298

Table S3. The phytonutrient profile of SA-GTE (sweet almond)

No.	Compound type	Phytonutrient	RT	M [+]	M [-]
1	Amino acid	Lysine	1.09	147.11336	
2	Alkaloid	Choline	1.18	104.10754	
3	Amino acid	Arginine	1.22	175.11951	
4	Carboxylic acid	Quinic acid	1.24		191.05557
5	Amino acid	γ-Aminobutyric acid	1.24	104.07116	
6	Amino acid	Aspartic acid	1.27	134.04534	
7	Amino acid	Methionine sulfoxide	1.27	166.05379	
8	Amino acid	Proline	1.27	116.07116	
9	Amino acid	Serine	1.27	106.05042	
10	Amino acid	Threonine	1.27	120.06607	
11	Amino acid	Asparagine	1.29	133.06132	
12	Amino acid	Glutamic acid	1.29	148.06099	
13	Vitamin	Adenine (B4)	1.30	136.06233	
14	Carboxylic acid	Malic acid	1.32		133.01370
15	Vitamin	Nicotinic acid (Niacin,B3)	1.40	124.03986	
16	Carboxylic acid	Citric acid	1.41		191.01918
17	Vitamin	Nicotinamide	1.41	123.05584	
18	Amino acid	Isoleucine	1.54	132.10246	
19	Amino acid	Leucine	1.90	132.10246	
20	Amino acid	Phenylalanine	3.25	166.08681	
21	Others	2-Oxindole	4.00	134.06060	
22	Carbohydrate	Benzyl-glucoside	10.39		269.10252
23	Polyphenol	Coumaroylquinic acid isomer 1	11.96		337.09235
24	Polyphenol	5-O-(4-Coumaroyl)quinic acid	12.60		337.09235
25	Polyphenol	Vanilloylglucose isomer 1	13.64		329.08726
26	Polyphenol	Vanilloylglucose isomer 2	14.08		329.08726
27	Polyphenol	Coumaroylglucose isomer 1	14.18		325.09235
28	Polyphenol	Chlorogenic acid (3-O-Caffeoylquinic acid)	14.28	355.10291	

29	Polyphenol	3-O-Feruloylquinic acid	14.57		367.10291
30	Polyphenol	trans-Melilotoside (trans-Glucosyl-2-hydroxycinnamate)	14.68		325.09235
31	Polyphenol	Coumaroylglucose isomer 2	15.11		325.09235
32	Polyphenol	Chryptochlorogenic acid (4-O-Caffeoylquinic acid)	15.61	355.10291	
33	Polyphenol	3-O-(4-Coumaroyl)quinic acid	15.67		337.09235
34	Polyphenol	Vanilloylglucose isomer 3	16.15		329.08726
35	Carbohydrate	Benzyl-primeveroside	16.56		401.14478
36	Polyphenol	Coumaroylquinic acid isomer 2	16.87		337.09235
37	Polyphenol	4-O-(4-Coumaroyl)quinic acid	17.55		337.09235
38	Flavonoid	Quercetin-3-O-rutinoside-7-O-glucoside	17.59		771.19839
39	Polyphenol	4-Coumaric acid	17.85		163.03952
40	Polyphenol	5-O-Feruloylquinic acid	18.01		367.10291
41	Polyphenol	4-O-Feruloylquinic acid	18.56		367.10291
42	Aldehid	Indole-4-carbaldehyde	19.04	146.06059	
43	Flavonoid	Kaempferol-O-(rahymnosyl)hexoside-O-hexoside	19.09		755.20347
44	Polyphenol	Coumaroylquinic acid isomer 3	19.19		337.09235
45	Flavonoid	Taxifolin (Dihydroquercetin)	19.33		303.05048
46	Polyphenol	Ferulic acid	19.35		193.05009
47	Flavonoid	Quercetin-O-(hexosyl)rutinoside	19.82		771.19839
48	Flavonoid	Quercetin-O-dihexoside isomer 1	19.96		625.14048
49	Alkaloid	Chelidonine	20.05	354.13415	
50	Flavonoid	Quercetin-O-dihexoside isomer 2	20.20		625.14048
51	Flavonoid	Tetrahydroxyflavanone-O-hexoside isomer 1	20.35		449.10839
52	Polyphenol	Isoferulic acid	20.41		193.05009
53	Flavonoid	Myricetin-O-hexoside	20.91		479.08257
54	Flavonoid	Kaempferol-O-(hexosyl)hexoside	21.47		609.14556
55	Flavonoid	Dihydrokaempferol (Aromadendrin, Katuranin)	21.94		287.05557
56	Alkaloid	Berberine	22.33	336.12358	
57	Flavonoid	Prunin (Naringenin-7-O-glucoside)	22.38		433.11347

58	Flavonoid	Tetrahydroxyflavanone-O-hexoside isomer 2	22.46		449.10839
59	Flavonoid	Hyperoside (Quercetin-3-O-galactoside, Hyperin)	22.69		463.08765
60	Flavonoid	Isoquercitrin (Hirsutrin, Quercetin-3-O-glucoside)	22.89		463.08765
61	Flavonoid	Rutin (Quercetin-3-O-rutinoside)	23.02	611.16122	
62	Flavonoid	Reinutrin (Reynoutrin, Quercetin-3-O-xyloside)	23.25		433.07709
63	Flavonoid	Avicularin (Quercetin-3-O-arabinofuranoside, Fenicularin)	23.50		433.07709
64	Flavonoid	Quercetin-3-O-(6''-malonyl)glucoside	23.68		549.08805
65	Other	Eugenol (4-Allyl-2-methoxyphenol)	23.81	165.09156	
66	Flavonoid	Tetrahydroxyflavanone-O-hexoside isomer 3	23.83		449.10839
67	Flavonoid	Kaempferol-O-hexoside	24.15		447.09274
68	Flavonoid	Guaijaverin (Quercetin-3-O-arabinoside)	24.26		433.07709
69	Flavonoid	Kaempferitrin (Kaempferol-3,7-di-O-rhamnoside)	24.31		577.15573
70	Flavonoid	Quercetin-O-(rhamnosyl)hexoside	24.31		609.14556
71	Flavonoid	Quercitrin (Quercetin-3-O-rhamnoside)	24.48	449.10839	
72	Flavonoid	Quercetin-O-(acetyl)hexoside isomer 1	24.49	507.11387	
73	Flavonoid	Astragalin (Kaempferol-3-O-glucoside)	24.69		447.09274
74	Flavonoid	Isorhamnetin-O-hexoside isomer 1	24.75		477.10330
75	Flavonoid	Kaempferol-3-O-rutinoside (Nicotiflorin)	24.86		593.15065
76	Flavonoid	Eriodictyol	24.94		287.05556
77	Flavonoid	Isorhamnetin-O-hexoside isomer 2	24.95		477.10330
78	Flavonoid	Isorhamnetin-3-O-rutinoside (Narcissin)	25.21		623.16122
79	Terpenoid	Abscisic acid	25.39		263.12834
80	Flavonoid	Kaempferol-O-(malonyl)glucoside	25.63		533.09314
81	Flavonoid	Quercetin-O-(acetyl)hexoside isomer 2	25.68	507.11387	
82	Flavonoid	Kaempferol-O-(rhamnosyl)hexoside	26.13		593.15065
83	Flavonoid	Quercetin-O-(acetyl)hexoside isomer 3	26.29	507.11387	
84	Flavonoid	2''-O-Acetylrutin	26.31		651.15613
85	Flavonoid	Quercetin-3-O-(4-coumaroyl)glucoside	26.85		609.12444
86	Polyphenol	Di-O-coumaroylglucose isomer 1	27.01		471.12913

87	Flavonoid	5,7,3',4',5'-Pentahydroxyflavone (Tricetin)	27.02		301.03483
88	Flavonoid	Naringenin	27.24		271.06065
89	Polyphenol	Di-O-coumaroylglucose isomer 2	27.38		471.12913
90	Flavonoid	Trihydroxy-methoxy(iso)flavanone	27.38		301.07122
91	Carboxylic acid	Jasmonic acid	27.77		209.11777
92	Flavonoid	Multiflorin A (Kaempferol-3-O-[(6-O-acetyl)glucosyl-(1→4)rhamnoside])	28.04		635.16121
93	Flavonoid	Trihydroxy-methoxyflavone-O-hexoside	28.37		461.10839
94	Flavonoid	Tetrahydroxyflavone	29.36		285.03991
95	Flavonoid	Isorhamnetin	29.84		315.05048
96	Flavonoid	Kaempferol-O-[(acetyl)rhamnosyl)rhamnoside]	29.96		619.16630
97	Flavonoid	Pinocembrin (5,7-Dihydroxyflavanone)	32.23		255.06573
98	Flavonoid	Dihydroxy-methoxy(iso)flavanone	32.25	287.09195	
99	Flavonoid	Dihydroxy-methoxy(iso)flavone	33.90		283.06065
100	Flavonoid	Trihydroxy-methoxy(iso)flavone	34.56		299.05557
101	Fatty acid	α-Linolenic acid	44.73		277.21676
102	Fatty acid	Linoleic acid	45.75		279.23241
103	Fatty acid	Palmitoleic acid	46.05		253.21676

Table S4. The phytonutrient profiles of BM-GTE (black mulberry)

No.	Compound type	Phytonutrient	RT	M [+]	M [-]
1	Amino acid	Lysine	1.08	147.11336	
2	Alkaloid	O-Hexosyl-1-deoxynojirimycin	1.08	326.14511	
3	Alkaloid	1,4-Dideoxy-1,4-iminoarabinitol	1.24	134.08172	
4	Amino acid	Arginine	1.24	175.11951	
5	Carboxylic acid	Quinic acid (metabolite)	1.25		191.05557
6	Alkaloid	1-Deoxynojirimycin (Moranoline)	1.29	164.09229	
7	Amino acid	Citrulline	1.30	176.10352	
8	Amino acid	Proline	1.30	116.07116	
9	Amino acid	Threonine	1.31	120.06607	
10	Vitamin	Adenine (B4)	1.32	136.06233	
11	Amino acid	Asparagine	1.32	133.06132	
12	Amino acid	Aspartic acid	1.34	134.04534	
13	Carboxylic acid	Citric acid	1.41		191.01918
14	Vitamin	Nicotinic acid (Niacin,B3)	1.41	124.03986	
15	Vitamin	Nicotinamide	1.42	123.05584	
16	Amino acid	Tyrosine	1.42	182.08172	
17	Carboxylic acid	Malic acid	1.44		133.01370
18	Amino acid	Isoleucine	1.54	132.10246	
19	Amino acid	Leucine	1.81	132.10246	
20	Amino acid	Phenylalanine	3.23	166.08681	
21	Polyphenol	Protocatechuic acid (3,4-Dihydroxybenzoic acid)	4.85		153.01879
22	Vitamin	Pantothenic acid (B5)	5.41	220.11850	
23	Amino acid	Tryptophan	8.06	205.09771	
24	Polyphenol	Neochlorogenic acid (5-O-Caffeoylquinic acid)	8.91	355.10291	
25	Polyphenol	2,4-Dihydroxybenzoic acid	12.23		153.01879
26	Polyphenol	5-O-(4-Coumaroyl)quinic acid	12.59		337.09235
27	Polyphenol	Chlorogenic acid (3-O-Caffeoylquinic acid)	14.23	355.10291	
28	Polyphenol	1-O-Caffeoylglucose	14.35		341.08726

29	Polyphenol	Caffeic acid	14.49		179.03444
30	Polyphenol	3-O-Feruloylquinic acid	14.57		367.10291
31	Polyphenol	trans-Melilotoside (trans-Glucosyl-2-hydroxycinnamate)	14.65		325.09235
32	Polyphenol	Cudranin-di-O-glucoside isomer 1	15.31		585.18195
33	Polyphenol	Chryptochlorogenic acid (4-O-Caffeoylquinic acid)	15.59	355.10291	
34	Polyphenol	Coumaroylquinic acid isomer	16.87		337.09235
35	Polyphenol	1-O-Caffeoylquinic acid	16.98		353.08726
36	Polyphenol	Cudranin-di-O-glucoside isomer 2	17.09		585.18195
37	Flavonoid	Quercetin-di-O-hexoside	17.18		625.14048
38	Ester	Ethyl gallate	17.36		197.04500
39	Polyphenol	4-O-(4-Coumaroyl)quinic acid	17.54		337.09235
40	Flavonoid	Quercetin-3-O-rutinoside-7-O-glucoside	17.57		771.19839
41	Polyphenol	12-Hydroxyjasmonic acid-12-O-glucoside or Tuberonic acid glucoside	17.59		387.16551
42	Polyphenol	Hydroxycoumarin	17.75		161.02387
43	Polyphenol	Cudranin-O-glucoside isomer 1	17.84	407.13421	
44	Polyphenol	5-O-Feruloylquinic acid	17.98		367.10291
45	Carboxylic acid	12-Hydroxyjasmonic acid	18.04		225.11269
46	Flavonoid	Quercetin-O-hexoside-O-(malonyl)hexoside	18.19		711.14087
47	Carboxylic acid	Tuberonic acid	18.27		225.11269
48	Polyphenol	Cudranin-O-glucoside isomer 2	18.33	407.13421	
49	Polyphenol	4-O-Feruloylquinic acid	18.51		367.10291
50	Polyphenol	Scopoletin	18.55	193.05009	
51	Vitamin	Riboflavin (B2)	18.60	377.14611	
52	Flavonoid	Kaempferol-O-(rhamnosyl)hexoside-O-hexoside	19.07		755.20347
53	Polyphenol	3-O-(4-Coumaroyl)quinic acid	19.17		337.09235
54	Flavonoid	Taxifolin (Dihydroquercetin)	19.34		303.05048
55	Flavonoid	Kaempferol-O-hexoside-O-(malonyl)hexoside	19.80		695.14596
56	Alkaloid	Chelidonine	20.05	354.13415	
57	Flavonoid	Quercetin-O-(dihexoside)	20.21		625.14048

58	Polyphenol	Cudranin (Oxyresveratrol, 2,3',4,5'-Tetrahydroxystilbene)	21.33	245.08139	
59	Flavonoid	Isorhamnetin-3-O-sophoroside	21.83		639.15613
60	Flavonoid	Dihydrokaempferol (Aromadendrin, Katuranin)	21.94		287.05557
61	Flavonoid	Kaempferol-3-O-rhamnoside-7-O-[rhamnosyl-(1→2)-glucoside]	22.10		739.20856
62	Polyphenol	Dicaffeoylquinic acid isomer 1	22.33		515.11896
63	Flavonoid	Quercetin-3-O-glucuronide	22.73		477.06692
64	Flavonoid	Isoquercitrin (Hirsutrin, Quercetin-3-O-glucoside)	22.91		463.08765
65	Flavonoid	Rutin (Quercetin-3-O-rutinoside)	23.02	611.16122	
66	Flavonoid	Reinutrin (Reynoutrin, Quercetin-3-O-xyloside)	23.23		433.07709
67	Flavonoid	Avicularin (Quercetin-3-O-arabinofuranoside, Fenicularin)	23.51		433.07709
68	Flavonoid	Quercetin-O-(malonyl)hexoside	23.65		549.08805
69	Other	Eugenol (4-Allyl-2-methoxyphenol)	23.83	165.09156	
70	Other	Lumichrome	23.90	243.08821	
71	Polyphenol	Dicaffeoylquinic acid isomer 2	24.18		515.11896
72	Flavonoid	Guaijaverin (rn-3-O-arabinoside)	24.24		433.07709
73	Flavonoid	Astragalin (Kaempferol-3-O-glucoside)	24.69		447.09274
74	Polyphenol	Isoliquiritigenin	24.80		255.06573
75	Flavonoid	Kaempferol-3-O-rutinoside (Nicotiflorin)	24.84		593.15065
76	Flavonoid	Isorhamnetin-3-O-glucoside	24.93	479.11895	
77	Flavonoid	Eriodictyol	24.95		287.05557
78	Flavonoid	Isorhamnetin-O-hexoside-O-rhamnoside	25.17		623.16122
79	Terpenoid	Abscisic acid	25.39		263.12834
80	Flavonoid	Tetrahydroxyflavanone isomer	25.59		287.05557
81	Flavonoid	Kaempferol-O-(malonyl)hexoside	25.61		533.09314
82	Polyphenol	Ethyl caffeate	25.95	209.08139	
83	Flavonoid	Naringenin	27.22		271.06065
84	Carboxylic acid	Jasmonic acid	27.76		209.11777
85	Flavonoid	Luteolin (3',4',5,7-Tetrahydroxyflavone)	27.86		285.03992
86	Flavonoid	Methoxy-tetrahydroxy(iso)flavone	28.24		315.05048

87	Flavonoid	Norartocarpetin (2',4',5,7-Tetrahydroxyflavone)	28.41		285.03992
88	Ester	Ethyl 4-hydroxycinnamate	28.68		191.07082
89	Flavonoid	Kaempferol (3,4',5,7-Tetrahydroxyflavone)	29.34	287.05557	
90	Flavonoid	Apigenin	29.68		269.04500
91	Flavonoid	Chrysoeriol	29.89		299.05556
92	Flavonoid	Liquiritigenin (4',7-Dihydroxyflavanone)	29.98		255.06573
93	Flavonoid	Sakuranetin (4',5-Dihydroxy-7-methoxyflavanone)	32.04	287.09195	
94	Flavonoid	Pinocembrin (5,7-Dihydroxyflavanone)	32.24		255.06573
95	Flavonoid	Kaempferol-O-(cinnamoyl)hexoside	32.27		577.13461
96	Polyphenol	Mornigrol D	33.44	395.18585	
97	Flavonoid	Acacetin (Linarigenin, 5,7-Dihydroxy-4'-methoxyflavone)	33.90	285.07630	
98	Flavonoid	Dihydroxy-dimethoxy(iso)flavone isomer 1	33.99	315.08687	
99	Flavonoid	Dihydroxy-dimethoxy(iso)flavone isomer 2	34.85	315.08687	
100	Flavonoid	Dihydroxy-trimethoxyflavone	34.92		343.08178
101	Flavonoid	Mornigrol E or Mornigrol F	35.29	439.17568	
102	Flavonoid	Kuwanon G (Albanin F, Moracenin B)	36.38		691.21794
103	Flavonoid	Kuwanon C (Mulberrin) or Albanin E or Nigrasin H	37.64	423.18077	
104	Flavonoid	Apigenin-7,4'-dimethyl ether	38.16	299.09195	
105	Flavonoid	Albafuran A or Albafuran B	38.33	379.19094	
106	Flavonoid	Albafuran A or Albafuran B	38.56	379.19094	
107	Flavonoid	Kuwanon E	40.00		423.18077
108	Flavonoid	Kuwanon A or Kuwanon B	40.87	421.16512	
109	Flavonoid	Chalcomoracin	40.96		647.22811
110	Flavonoid	Kuwanon A or Kuwanon B	41.24	421.16512	
111	Flavonoid	Cyclomorusin (Cyclomulberrochromene)	44.56		416.12599
